# Anticancer Tungstenocenes with a Diverse Set of (*O,O*–), (*O*,*S*–) and (*O*,*N*–) Chelates—A Detailed Biological Study Using an Improved Evaluation via 3D Spheroid Models

**DOI:** 10.3390/pharmaceutics15071875

**Published:** 2023-07-03

**Authors:** Klaudia Cseh, Iker Berasaluce, Valentin Fuchs, Alexandra Banc, Andreas Schweikert, Alexander Prado-Roller, Michaela Hejl, Debora Wernitznig, Gunda Koellensperger, Michael A. Jakupec, Wolfgang Kandioller, Michael S. Malarek, Bernhard K. Keppler

**Affiliations:** 1 Institute of Inorganic Chemistry, Faculty of Chemistry, University of Vienna, Waehringer Straße 42, A 1090 Vienna, Austria; klaudia.cseh@univie.ac.at (K.C.); valentin.fuchs@univie.ac.at (V.F.); andreas.schweikert@univie.ac.at (A.S.); alexander.roller@univie.ac.at (A.P.-R.); michaela.hejl@univie.ac.at (M.H.); michael.jakupec@univie.ac.at (M.A.J.); wolfgang.kandioller@univie.ac.at (W.K.); bernhard.keppler@univie.ac.at (B.K.K.); 2 Institute of Analytical Chemistry, Faculty of Chemistry, University of Vienna, Waehringer Straße 38, A 1090 Vienna, Austria; gunda.koellensperger@univie.ac.at; 3 Research Cluster “Translational Cancer Therapy Research”, University of Vienna, Waehringer Straße 42, A 1090 Vienna, Austria

**Keywords:** metal-based drugs, tungstenocenes, anticancer, 3D cell culture, multicellular tumor spheroids

## Abstract

The synthesis, characterization and biological activity of tungstenocenes with varying biologically active (*O*,*O*–), (*S*,*O*–*)* and (*N*,*O*–*)* chelates are described. Complexes were characterized by ^1^H and ^13^C NMR, elemental analysis, ESI-mass spectrometry, FT-IR spectroscopy and X-ray diffraction analysis. The aqueous stability was studied by UV/Vis spectroscopy and the W^IV^ to W^V^ process by cyclic voltammetry. The cytotoxicity was determined by the MTT assay in A549, CH1/PA-1 and SW480 cancer cells as well as in IMR-90 human fibroblasts. Extensive biological evaluation was performed in three other human cancer cell lines (HCT116, HT29 and MCF-7) in monolayer and multicellular tumor spheroid cultures to better understand the mode of action. Lead compounds showed promising in vitro anticancer activity in all cancer cell lines. Further studies yielded important insights into apoptosis induction, ROS generation, different patterns in metal distribution (detected by LA-ICP-TOF-MS), changes in KI67 (proliferation marker) expression and DNA interactions. The results based on qualitative and quantitative research designs show that complexes containing (*S*,*O*–) chelates are more active than their (*O*,*O*–) and (*N*,*O*–) counterparts. The most striking results in spheroid models are the high antiproliferative capacity and the different distribution pattern of two complexes differing only in a W–S or W–O bond.

## 1. Introduction

Over the past 30 years, the field of bioorganometallic chemistry with advances in catalytic and medicinal applications has risen in importance. A major focus currently is the search for potential cancer drugs with fewer and less severe side effects than those currently employed [1,2,3]. Our work focuses on group 6 metal complexes containing molybdenum, the only second row transition metal necessary for life, and its heavier analogue tungsten. Molybdenum is easily transported within and excreted from the human body as molybdate. Both excess molybdenum and tungsten are removed in the urine and should be generally less toxic than other metals [4,5]. The group 6 ‘bent’ metallocenes allow for additional ligands to be bound to the metal center and result in a decrease in the angle between the Cp rings. The molybdenocenes and tungstenocenes, therefore, bear a *cis*-dihalido motif like cisplatin, the initial reason for their interest in anticancer research [1]. By replacing the halides with bioactive chelating ligands, we aim to synthesize anticancer agents which are more tolerable, stable, and ideally would show synergistic effects between the metal and ligand.

Köpf and Köpf–Maier first described the antitumor activity of several metal-based organometallic complexes containing titanium, vanadium, niobium, molybdenum, tungsten and rhenium in 1979 [6,7,8,9]. Around two decades later, several research groups reported anticancer activities for neutral and charged metallocene derived compounds of these metals [10,11,12,13,14,15]. However, tungstenocenes were abandoned due to their lower activity and stability when compared to the other metallocenes. Anticancer research with tungstenocenes, therefore, is very rare; in fact, the only published work in this area is from the Köpf/Köpf-Maier and Meléndez groups [8,16].

**Figure 1:**
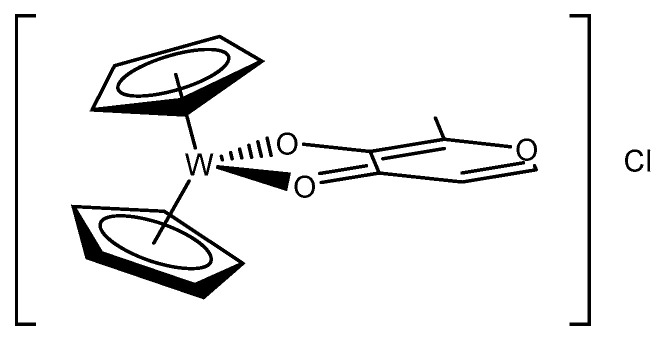
[Cp_2_W(maltolato)]Cl complex synthesized by Meléndez and coworkers [16].

While titanocene dichloride exhibited the highest cytotoxic activity in several tumor cell lines, it also showed negative side effects like cisplatin and was abandoned in clinical phase II [17,18]. Molybdenocene dichloride has a unique and potentially useful stability of the metal-cyclopentadienyl bonds under physiological conditions [19]; however, the same stability is not observed in the tungstenocenes. All metallocenes readily lose the chlorido ligands and form aqua, hydroxido or even oxo-bridged dimeric species in aqueous solutions [19,20]. The exchange of the dichlorido motif for chelates to increase stability as well as solubility has been a popular approach explored by various research groups [16,21,22]. The cytotoxic activity of Cp_2_WCl_2_ bearing 3-hydroxy-4-pyrone ligands was reported by Meléndez and coworkers in 2013 (Figure 1), showing an improved in vitro cytotoxicity of tungstenocenes bearing (*O,O*–) donor ligands, when compared to Cp_2_WCl_2_, in HT29 colon cancer and MCF-7 breast cancer cell lines [16].

Due to the increased stability of the molybdenocenes they have been studied to a greater degree than the more unstable tungsten compounds, the former showing promising in vitro anticancer activity in many different cell lines [21,22,23]. Nevertheless, with the correct choice of chelating ligands (containing softer sulfur donors) an improved stability of the tungstenocenes can be attained, making this class of compounds more interesting, especially if they exhibit activity against cancer cells.

This paper presents the synthesis, characterization and biological studies of ten tungstenocenes containing an (*O*,*O*–), (*S*,*O*–) or (*N*,*O*–) chelate. A comparison of their cytotoxic activities with previous research and preliminary mode-of-action studies are presented. The bidentate ligand scaffolds were coordinated to the already cytotoxic tungstenocene dichloride, enhancing the cytotoxic activity further as well as increasing the stability in aqueous media [16]. The impact on the aqueous stability of the W–S vs. W–O bonds and counter ion exchange from chloride to hexafluorophosphate is discussed. Previous studies from our research group reported that attempts to synthesize molybdenocene derivatives with chloride as counter ion did not yield any product; however, exchanging the chloride for PF_6_^−^ (hexafluorophosphate anion) allowed for the successful isolation of the products [21]. This anion exchange allowed for a more straightforward purification of the complexes, avoiding size-exclusion column chromatography and resulted in improved yields.

A broad range of ligand scaffolds were utilized in this work, including thiol derivatives of 3-hydroxy-4-pyrones, demonstrated to improve the stability as well as the cytotoxic activity in vitro [21,24]. We have made use of maltol (3-hydroxy-2-methyl-4-(1*H*)-pyrone), a natural product which is commonly utilized as a ligand with a broad range of biologically relevant metals because of its potentially beneficial effect on bioavailability [25,26,27,28,29,30]. Allomaltol and thioallomaltol are interesting since we have shown that this small change in the methyl group from the 3-position in maltol and thiomaltol to the 5-position has a significant effect on the biological activity of the resultant complexes [31]. Ethylmaltol, already employed by Meléndez and coworkers [16] as a ligand for tungstenocene, was also used for comparison purposes as well as its thioethylmaltol analogue. Besides pyrones, flavonoids also possess antioxidant and antiproliferative properties [32], and their thiol derivatives have also been employed as ligands in our work [31]. Picolinic acid has demonstrated many positive effects in the human body, such as immunological, neuroprotective and antiproliferative effects [33], while deferiprone has been shown to selectively kill tumor cells over normal cells [34]. We attempted to determine which chelating motif is most promising as well as to identify structure-activity relationships for the maltol-based complexes.

## 2. Materials and Methods

### 2.1. Experimental Part

All utilized solvents, purchased from commercial sources, were of HPLC grade and used without any further purification, except for methanol, which was dried over molecular sieves (3 Å) prior to use [35]. Bis(cyclopentadienyl)tungsten dichloride (99%, Abcr, Karlsruhe, Germany), sodium methoxide (~95%, Fluka), ammonium hexafluorophosphate (99%, Sigma-Aldrich, Vienna, Austria), Lawesson’s reagent (99%, Acros Organics, Geel, Belgium), PBS (sterile filtered, Sigma Life Science, Vienna, Austria), maltol (**L1**) (99%, Sigma-Aldrich), picolinic acid (**L5**) (99%, Sigma-Aldrich), deferiprone (**L6**) (98%, Sigma-Aldrich) and ethylmaltol (**L9**) (99%, Sigma-Aldrich) were purchased from the respective commercial source and used as obtained. Thiomaltol (**L2**) [36], 3-hydroxy-2-(4′-chlorophenyl)chromen-4-one (**L3**) [37] and allomaltol [26] were synthesized according to the literature procedures and 3-hydroxy-2-(4′-chlorophenyl)-chromen-4-thione (**L4**), thioallomaltol (**L8**) [38], thioethylmaltol (**L10**) [39] were synthesized using Lawesson’s reagent [36] (see Supporting Information for synthesis and characterization). The synthesis of Bis(η^5^-cyclopentadienyl)[2-methyl-3-(oxo-κO)-4-(1*H*)-pyron-4-ato-κO]tungsten(IV) chloride (**1A**) was modified compared with the one in the literature [16].

NMR spectra were recorded with a Bruker FT-NMR Avance III^TM^ 500 MHz spectrometer at 500.10 (^1^H), and 125.75 MHz (^13^C) using DMSO–d_6_. 2D-NMR measurements were recorded utilizing standard pulse programs COSY, HSQC and HMBC. The Microanalytical Laboratory of the University of Vienna performed the elemental analyses with a Eurovector EA 3000(2009) equipped with a high temperature pyrolysis furnace (HT, Hekatech, Germany, 2009). Elemental analyses’ samples were weighed (weights from 1–3 mg were used) on a Sartorius SEC 2 ultra-micro balance with ±0.1 µg resolution. Two NIST-certified reference materials were used for calibration: sulfanilamide (C_6_H_8_N_2_O_2_S) and BBOT (2,5-bis-(5-tert-butyl-2-benzoxazol-2-yl)-thiophenone, C_26_H_26_N_2_O_2_S). The limit of quantification (LOQ) was 0.05 w% for C, H, N and 0.02 w% for S. For samples without N and/or S, the content of these elements was determined and verified to be below the LOQ. X-ray diffraction analyses were carried out on a Bruker X8 APEX II CCD diffractometer at 100 K. ESI-MS were recorded on a Bruker AmaZon SL ion trap mass spectrometer (Bruker Daltonics GmbH). Data were obtained and processed with Compass 1.3 and Data Analysis 4.0 (Bruker Daltonics GmbH). Infrared spectra were attained on a Bruker Vertex 70 FT-IR-spectrometer with an ATR-unit (attenuated total reflection unit) in the range of 4000–600 cm^−1^. Intensities of the reported bands are described with w for weak, m for medium and s for strong; broad signals are additionally specified with the letter b in front of these abbreviations. Cyclic voltammograms (CVs) were measured in a three-electrode cell using 2 mm diameter glassy carbon disk working electrode, a platinum auxiliary electrode and an Ag|Ag^+^ reference electrode containing 0.1 M AgNO_3_. Measurements were carried out at room temperature using an EG&G PARC potentiostat/galvanostat 273A. The solutions were deoxygenized by purging a stream of argon for 3 min through the solution and the experiment was performed under an argon atmosphere. The potentials were measured in a freshly prepared solution of (*n* Bu_4_N)[BF_4_] (0.1 M) in acetonitrile using ferrocene (Fe(η^5^-C_5_H_5_)_2_) (E_1/2_ = +0.72 V vs. NHE) [40] as an internal standard and are quoted relative to the normal hydrogen electrode (NHE).

Stock solutions in DMSO of compounds **1**–**10** were utilized to determine the solubility in PBS by adding 1 vol% of the stock solution to a suitable amount of PBS. The solubility in aqueous media of compounds **1**–**10** was sufficient (0.01–1.20 mg/mL) for biological experiments. UV/Vis data were recorded on a Perkin Elmer Lambda 650 UV/Vis Spectrophotometer with a Peltier element for temperature control.

### 2.2. Synthesis

#### 2.2.1. General Complexation Synthesis A

Bis(cyclopentadienyl)tungsten dichloride (1 eq.), ligand (1–1.1 eq.) and sodium methoxide (1.1 eq.) were dissolved in 20–25 mL dry methanol (over molecular sieves 3Å) and stirred for 4–72 h at room temperature or reflux under argon. After any solid impurities were removed by filtration, ammonium hexafluorophosphate (1.1–4.5 eq.) was added and stirred for a further 2–4 h. The formed precipitate was filtered, washed with methanol, for some samples taken up into dichloromethane to filter off impurities and, subsequently, the solvent was removed and then dried in vacuo.

#### 2.2.2. General Complexation Synthesis B

Ligand (1.2 eq.) and sodium methoxide (1.4 eq.) were dissolved in 24 mL dry methanol (dried over molecular sieves 3Å) and stirred at room temperature under argon for 10 min. In a separate Schlenk flask, bis(cyclopentadienyl)tungsten dichloride (130 mg, 0. 33 mmol, 1 eq.) was dissolved in dry dichloromethane (8 mL), the prior solution was added via cannula and the resulting mixture was stirred at room temperature for 20 h. Sodiumhexafluorphosphate (2 eq.) was added, and the mixture was stirred for another 2 h.

##### Bis(η^5^-cyclopentadienyl)[2-methyl-3-(oxo-κO)-4-(1H)-pyran-4-ato-κO]tungsten(IV) hexafluorophosphate (**1**)

The synthesis was performed according to the general procedure A using bis(cyclopentadienyl)tungsten dichloride (135 mg, 350 µmol), 3-hydroxy-2-methyl-4*H*-pyran-4-one (48 mg, 385 µmol) and sodium methoxide (21 mg, 385 µmol) with a reaction time of 16 h. Ammonium hexafluorophosphate (63 mg, 385 µmol) was added and stirred for 2 h. The formed precipitate was filtered and washed with methanol. Yield: 119 mg (58%), brown powder. Solubility in PBS (1% DMSO): 0.23 mg/mL. ^1^H-NMR (DMSO-d_6_) *δ* = 2.35 (s, H, CH_3_) 5.90 (s, 10H, Cp), 7.03 (d, ^3^*J*(H, H) = 5.0 Hz, H, C5-H), 8.34 (d, ^3^*J*(H, H) = 5.0 Hz, H, C6-H) ppm. ^13^C-NMR (DMSO-d_6_) δ = 14.5 (CH_3_), 98.1 (Cp), 110.4 (C5), 156.6 (C6), 157.5 (C2), 161.5 (C3), 187.0 (C4=O) ppm. Elemental analysis: Calculated for C_16_H_15_F_6_O_3_PW: C, 32.90; H, 2.59; P, 5.30%. Found: C, 32.79; H, 2.63; P, 5.07%. (ESI^+^) *m*/*z*: 439.05 [Cp_2_W(maltolate)]^+^. IR: ν = 3128 w (ν_C–H_, Cp), 1604, 1547 m (ν_C=O_), 1476, 1429 m (ν_C=C_), 820 s (ν_C–H_) cm^−1^.

##### Bis(η^5^-cyclopentadienyl)[2-methyl-3-(oxo-κO)-4-(1H)-pyran-4-ato-κO]tungsten(IV) chloride (**1A**)

Bis(cyclopentadienyl)tungsten dichloride (128 mg, 330 µmol), 3-hydroxy-2-methyl-4*H*-pyran-4-one (42 mg, 330 µmol) and sodium methoxide (18 mg, 330 µmol) were dissolved in 20 mL methanol (dried over molecular sieves 3Å) and stirred for 16 h at room temperature under an argon atmosphere. The solvent was removed in vacuo and the remaining solids were dissolved in dichloromethane. Afterwards, the mixture was filtered leaving a blue insoluble precipitate behind. Dichloromethane was removed under reduced pressure and the product was washed several times with THF, followed by diethyl ether. Yield: 60 mg (42%), brown powder. Solubility in PBS (1% DMSO): 5.00 mg/mL. Elemental analysis: Calculated for C_16_H_15_ClO_3_W·0.75H_2_O: C, 39.37; H, 3.41%. Found: C, 39.48; H, 3.68%. (ESI^+^) *m*/*z*: 439.03 [Cp_2_W(maltolate)]^+^.

##### Bis(η^5^-cyclopentadienyl)[2-methyl-3-(oxo-κO)-4-(1H)-pyran-4-thionato-κS]tungsten(IV) hexafluorophosphate (**2**)

The synthesis was performed according to the general procedure A using bis(cyclopentadienyl)tungsten dichloride (135 mg, 350 µmol), 3-hydroxy-2-methyl-4*H*-pyran-4-thione (50 mg, 350 µmol) and sodium methoxide (19 mg, 350 µmol) with a reaction time of 48 h. Ammonium hexafluorophosphate (118 mg, 700 µmol) was added and stirred for 3 h. The formed precipitate was filtered and washed with methanol. Yield: 108 mg (52%), red powder. Solubility in PBS (1% DMSO): 0.25 mg/mL. ^1^H-NMR (DMSO-d_6_) *δ* = 2.34 (s, H, CH_3_) 5.70 (s, 10H, Cp), 7.83 (d, ^3^*J*(H, H) = 4.6 Hz, H, C5-H), 8.21 (d, ^3^*J*(H, H) = 4.6 Hz, H, C6-H) ppm. ^13^C-NMR (DMSO-d_6_) δ = 15.3 (CH_3_), 96.3 (Cp), 119.4 (C5), 149.7 (C6), 156.7 (C2), 171.4 (C3), 181.5 (C4=S) ppm. Elemental analysis: Calculated for C_16_H_15_F_6_O_2_PSW: C, 32.02; H, 2.52, S, 5.34%. Found: C, 32.28; H, 2.45; S, 5.01%. (ESI^+^) *m*/*z*: 455.17 [Cp_2_W(thiomaltolate)]^+^. IR: ν = 3124 m (ν_C–H_, Cp), 1583, 1505 m (ν_C=O_), 1470, 1424 m (ν_C=C_), 822 s (ν_C–H_) cm^−1^.

##### Bis(η^5^-cyclopentadienyl)[2-(4-chlorophenyl)-3-(oxo-κO)-4(H)-chromen-4-oato-κO]tungsten(IV) hexafluorophosphate (**3**)

The synthesis was performed according to the general procedure A using bis(cyclopentadienyl)tungsten dichloride (128 mg, 300 µmol), 3-hydroxy-2-(4′-chlorophenyl)chromen-4-one (90 mg, 330 µmol) and sodium methoxide (18 mg, 330 µmol) with a reaction time of 72 h at 40 °C. Ammonium hexafluorophosphate (270 mg, 1.50 mmol) was added and stirred for 2 h. After washing with MeOH the precipitate was dissolved in DCM and the remaining solids were filtered off. Yield: 69 mg (32%), green powder. Solubility in PBS (1% DMSO): 0.03 mg/mL. ^1^H-NMR (DMSO-d_6_) *δ* = 6.06 (s, 10H, Cp), 7.64 (m, 1H, C7-H), 7.69 (d, ^3^*J*(H, H) = 8.7 Hz, 2H, C3′-H’ and C5′-H), 7.97–8.02 (m, 1H, C8-H), 8.03 (d, ^3^*J*(H, H) = 8.0 Hz, 1H, C6-H), 8.08 (d, ^3^*J*(H, H) = 8.0 Hz, 1H, C9-H), 8.37 (d, ^3^*J*(H, H) = 8.7 Hz, 2H, C2′-H and C6′-H) ppm. ^13^C-NMR (DMSO-d_6_) δ = 98.5 (Cp), 118.0 (C5), 118.4 (C6-H), 123.9 (C9-H), 126.2 (C7-H), 129.0 (C3′-H and C5′-H), 129.2 (C1′), 129.3 (C2′-H and C6′-H), 135.3 (C8-H), 136.0 (C4′), 150.7 (C2), 154.2 (C10), 158.4 (C3), 188.8 (C4=O) ppm. Elemental analysis: Calculated for C_25_H_18_ClF_6_O_3_PW: C, 41.09; H, 2.48%. Found: C, 41.22; H, 2.55%. (ESI^+^) *m*/*z*: 585.05 [Cp_2_W(flavonolate)]^+^. IR: ν = 3121 w (ν_C–H_, Cp), 1587, 1536 w (ν_C=O_), 1489, 1430 s (ν_C=C_), 823 s (ν_C–H_) cm^−1^.

##### Bis(η^5^-cyclopentadienyl)[2-(4-chlorophenyl)-3-(oxo-κO)-4(H)-chromen-4-thionato-κS]tungsten(IV) hexafluorophosphate (**4**)

The synthesis was performed according to the general procedure A using bis(cyclopentadienyl)tungsten dichloride (128 mg, 300 µmol), 3-hydroxy-2-(4′-chlorophenyl)chromen-4-thione (94 mg, 330 µmol) and sodium methoxide (18 mg, 330 µmol) with a reaction time of 48 h under reflux. Ammonium hexafluorophosphate (54 mg, 330 µmol) was added and stirred for 4 h. The formed precipitate was filtered off, dissolved in dichloromethane and filtered off in order to remove insoluble materials. Yield: 94 mg (39%), brown powder. Solubility in PBS (1% DMSO): 0.01 mg/mL. ^1^H-NMR (DMSO-d_6_) *δ* = 5.85 (s, 10H, Cp), 7.69 (d, ^3^*J*(H, H) = 8.7 Hz, 2H, C3′-H and C5′-H), 7.70–7.76 (m, 1H, C7-H), 7.96–8.01 (m, 1H, C8-H), 8.14 (d, ^3^*J*(H, H) = 8.0 Hz, 1H, C9-H), 8.33 (d, ^3^*J*(H, H) = 8.0 Hz, 1H, C6-H), 8.42 (d, ^3^*J*(H, H) = 8.7 Hz, 2H, C2′-H and C6′-H) ppm. ^13^C-NMR (DMSO-d_6_) δ = 96.5 (Cp), 118.7 (C9-H), 124.7 (C5), 125.7 (C6-H), 127.6 (C7-H), 129.1 (C3′-H and C5′-H), 129.1 (C1′), 130.5 (C2′-H and C6′-H), 134.4 (C8-H), 136.7 (C4′), 148.3 (C2), 149.3 (C10), 169.4 (C3), 187.2 (C4=S) ppm. Elemental analysis: Calculated for C_25_H_18_ClF_6_O_2_SPW: C, 40.21; H, 2.43; S, 4.29%. Found: C, 39.95; H, 2.71; S, 4.26%. (ESI^+^) *m*/*z*: 601.02 [Cp_2_W(thioflavonolate)]^+^. IR: ν = 3124 w (ν_C–H_, Cp), 1580 w (ν_C=O_), 1479, 1426 m (ν_C=C_), 817 s (ν_C–H_) cm^−1^.

##### Bis(η^5^-cyclopentadienyl)[2-(carboxylato-κO)-pyridine-κN]tungsten(IV) hexafluorophosphate (**5**)

The synthesis was performed according to the general procedure B using bis(cyclopentadienyl)tungsten dichloride (130 mg, 330 µmol), picolinic acid (50 mg, 400 µmol) and sodium methoxide (26 mg, 460 µmol). Afterwards, sodium hexafluorophosphate (112 mg, 660 µmol) was added and stirred for 2 h. The resulting solids were filtered off, redissolved in water and filtered through a folded filter. The filtrate was concentrated to 5 mL and stored at 4 °C overnight. The formed precipitate was filtered off, washed with THF and n-hexane and dried in vacuo. Yield: 52 mg (27%), red powder. Solubility in PBS (1% DMSO): 0.18 mg/mL. ^1^H-NMR (DMSO-d_6_) *δ* = 6.01 (s, 10H, Cp), 7.85–7.91 (m, 1H, C5-H), 8.16 (d, ^3^*J*(H, H) = 7.7 Hz, 1H, C3-H), 8.46–8.52 (m, 1H, C4-H), 9.09 (d, ^3^*J*(H, H) = 5.3 Hz, 1H, C6-H) ppm. ^13^C-NMR (DMSO-d_6_) δ = 97.2 (Cp), 126.5 (C3-H), 129.8 (C5-H), 143.4 (C4-H), 150.4 (C6-H), 160.0 (C2), 178.8 (C7=O) ppm. Elemental analysis: Calculated for C_16_H_14_F_6_NO_2_PW·0.5H_2_O: C, 32.57; H, 2.56; N, 2.37%. Found: C, 32.54; H, 2.45; N, 2.33%. (ESI^+^) *m*/*z*: 436.05 [Cp_2_W(picolinato)]^+^. IR: ν = 3121 m (ν_C–H_, Cp), 1684, 1610 s (ν_C=O_), 1484, 1438 m (ν_C=C_), 817 s (ν_C–H_) cm^−1^.

##### Bis(η^5^-cyclopentadienyl)[1,2-dimethyl-3-(oxo-κO)-4-(1H)-pyridonato-κO]tungsten(IV) hexafluorophosphate (**6**)

The synthesis was performed according to the general procedure A using bis(cyclopentadienyl)tungsten dichloride (128 mg, 300 µmol), 3-hydroxy-1,2-dimethyl-4-(1*H*)-pyridone (46 mg, 330 µmol) and sodium methoxide (18 mg, 330 µmol) with a reaction time of 4 h. Ammonium hexafluorophosphate (54 mg, 330 µmol) was added and stirred for 2 h. The formed precipitate was filtered off and dissolved in dichloromethane. Afterwards, the insoluble materials were removed by filtration. Yield: 95 mg (54%), dark brown powder. Solubility in PBS (1% DMSO): 1.20 mg/mL. ^1^H-NMR (DMSO-d_6_) *δ* = 2.28 (s, 3H, 2-CH_3_), 3.84 (s, 3H, *N*-CH_3_), 5.78 (s, 10H, Cp), 6.68 (d, ^3^*J*(H, H) = 6.0 Hz, 1H, C5-H), 7.73 (d, ^3^*J*(H, H) = 6.0 Hz, 1H, C6-H) ppm. ^13^C-NMR (DMSO-d_6_) δ = 11.7 (2-CH_3_), 42.4 (*N*-CH_3_), 97.6 (Cp), 108.4 (C5-H), 135.7 (C6-H), 136.4 (C2), 162.6 (C3), 176.4 (C4=O) ppm. Elemental analysis: Calculated for C_17_H_18_F_6_NO_2_PW: C, 34.19; H, 3.04; N, 2.35%. Found: C, 34.08; H, 3.10; N, 2.35%. (ESI^+^) *m*/*z*: 452.08 [Cp_2_W(deferipronato)]^+^. IR: ν = 3125 w (ν_C–H_, Cp), 1610, 1555 w (ν_C=O_), 1496, 1462 m (ν_C=C_), 825 s (ν_C–H_) cm^−1^.

##### Bis(η^5^-cyclopentadienyl)[2-methyl-5-(oxo-κO)-4-(1H)-pyran-4-ato-κO] tungsten(IV) hexafluorophosphate (**7**)

The synthesis was performed according to the general procedure A using bis(cyclopentadienyl)tungsten dichloride (135 mg, 350 µmol), 5-hydroxy-2-methyl-4*H*-pyran-4-one (44 mg, 350 µmol) and sodium methoxide (21 mg, 385 µmol) with a reaction time of 72 h. Ammonium hexafluorophosphate (118 mg, 700 µmol) was added and stirred for 4 h. Yield: 78 mg (38%), brown powder. Solubility in PBS (1% DMSO): 0.35 mg/mL. ^1^H-NMR (d_6_-DMSO): *δ* = 2.48 (s, 3H, H7), 5.91 (s, 10H, H_Cp_), 6.98 (s, 1H, H3), 8.31 (s, 1H, H6). ^13^C-NMR (d_6_-DMSO): δ = 19.83 (C7), 98.53(Cp), 109.61 (C3), 144.72 (C6), 163.61 (C5), 169.95 (C2), 190.41 (C4=O). Elemental analysis: Calculated for C_16_H_15_F_6_O_3_PW: C, 32.90; H, 2.59%. Found: C, 32.72; H, 2.58%. (ESI^+^) *m*/*z*: 439.02 [Cp_2_W(allomaltolato)]^+^. IR: ν = 3118 w (ν_C–H_, Cp), 1606, 1551 m (ν_C=O_), 1472, 1433 m (ν_C=C_), 819 s (ν_C–H_) cm^−1^.

##### Bis(η^5^-cyclopentadienyl)[2-methyl-5-(oxo-κO)-pyran-4-(1H)-thionato-κS] tungsten(IV) hexafluorophosphate (**8**)

The synthesis was performed according to the general procedure A using bis(cyclopentadienyl)tungsten dichloride (135 mg, 350 µmol), 5-hydroxy-2-methyl-4*H*-pyran-4-thione (50 mg, 350 µmol) and sodium methoxide (21 mg, 385 µmol) with a reaction time of 72 h. Ammonium hexafluorophosphate (114 mg, 700 µmol) was added and stirred for 4 h. The flask was stored at 4 °C overnight. The formed precipitate was filtered off and dissolved in dichloromethane and potential salt rests were filtered off. Yield: 88 mg (42%), red powder. Solubility in PBS (1% DMSO): 0.41 mg/mL. ^1^H-NMR (d_6_-DMSO): *δ* = 2.48 (s, 3H, H7), 5.70 (s, 10H, H_Cp_), 7.86 (s, 1H, H3), 8.15 (s, 1H, H6). ^13^C-NMR (d_6_-DMSO): δ = 18.97 (C7), 96.61 (Cp), 119.36 (C3), 142.92 (C6), 162.85 (C5), 172. 97 (C2), 187.96 (C4=S). Calculated for C_16_H_15_F_6_O_2_PSW: C, 32.02; H, 2.52; S, 5.34%. Found: C, 31.68; H, 2.48; S, 5.57%. (ESI^+^) *m*/*z*: 454.98 [Cp_2_W(thioallomaltolato)]^+^. IR: ν = 3126 m (ν_C–H_, Cp), 1587, 1510 m (ν_C=S_), 1439 m (ν_C=C_), 826 s (ν_C–H_) cm^−1^.

##### Bis(η^5^-cyclopentadienyl)[2-ethyl-3-(oxo-κO)-4-(1H)-pyran-4-ato-κO] tungsten(IV) hexafluorophosphate (**9**)

The synthesis was performed according to the general procedure B using bis(cyclopentadienyl)tungsten dichloride (130 mg, 330 µmol), 2-ethyl-3-hydroxy-4H-pyran-4-one (56 mg, 400 µmol) and sodium methoxide (26 mg, 460 µmol). Sodium hexafluorophosphate (112 mg, 660 µmol) was added and stirred for 2 h. The solvents were removed, the residue was taken up into dichloromethane, washed with water (3 × 20 mL), dried over magnesium sulfate and filtered through a folded filter. The solution was concentrated to 5 mL and the product was precipitated through the addition of diethyl ether, filtered, washed with n-hexane and dried in vacuo. Yield: 73 mg (37%), brown powder. Solubility in PBS (1% DMSO): 0.55 mg/mL. ^1^H-NMR (d_6_-DMSO): *δ* = 1.14 (t, 3H, ^3^*J* (H, H) = 7.6 Hz, H8), 2.71 (q, 2H, ^3^*J* (H, H) = 7.6 Hz, H7), 5.91 (s, 10H, H_Cp_), 7.06 (d, 1H, ^3^*J* (H, H) = 5.0 Hz, H5), 8.39 (d, 1H, ^3^*J* (H, H) = 5.0 Hz, H6). ^13^C-NMR (d_6_-DMSO): δ = 11.3 (C8), 21.95 (C7), 98.57 (Cp), 110.88 (C5), 157.16 (C6), 161.3 (C2), 161.9 (C3), 187.74(C4=O). Calculated for C_17_H_17_F_6_O_3_PW: C, 34.14; H, 2.86%. Found: C, 34.06; H, 2.87%. (ESI^+^) *m*/*z*: 452.97 [Cp_2_W(ethylmaltolato)]^+^. IR: ν = 3125 w (ν_C–H_, Cp), 1597, 1522 m (ν_C=O_), 1475, 1429 m (ν_C=C_), 998, 945, s (ν_C–C_), 819 s (ν_C–H_), 725 s (ν_–CH2–_) cm^−1^.

##### Bis(η^5^-cyclopentadienyl)[2-ethyl-3-(oxo-κO)-4-(1H)-pyran-4-thionato-κS] tungsten(IV) hexafluorophosphate (**10**)

The synthesis was performed according to the general procedure B using bis(cyclopentadienyl)tungsten dichloride (130 mg, 330 µmol), 2-ethyl-3-hydroxy-4H-pyran-4-thione (62 mg, 400 µmol) and sodium methoxide (26 mg, 460 µmol). Sodium hexafluorophosphate (112 mg, 660 µmol) was added and stirred for 2 h. The solvents were removed, the residue was taken up into dichloromethane, washed with water (3 × 20 mL), dried over magnesium sulfate and filtered through a folded filter. The solution was concentrated to 5 mL and the product was precipitated through the addition of diethyl ether, filtered, washed with n-hexane and dried in vacuo. Yield: 126 mg (62%), dark green powder. Solubility in PBS (1% DMSO): 0.32 mg/mL. ^1^H-NMR (d_6_-DMSO): *δ* = 1.12 (t, 3H, ^3^*J* (H, H) = 7.6 Hz, CH_3_), 2.70 (q, 2H, ^3^*J* (H, H) = 7.5 Hz, CH_2_), 5.71 (s, 10H, H_Cp_), 7.86 (d, 1H, ^3^*J* (H, H) = 4.6 Hz, H5), 8.25 (d, 1H, ^3^*J* (H, H) = 4.6 Hz, H6). ^13^C-NMR (d_6_-DMSO): δ = 10.94 (C8), 22.57 (C7), 96.74 (Cp), 119.89 (C5), 150.25 (C6), 160.77 (C2), 171.30 (C3), 182.38 (C4=S). Calculated for C_17_H_17_F_6_O_2_PSW: C, 33.24; H, 2.79; S, 5.22%. Found: C, 33.21; H, 2.80; S, 5.23%. (ESI^+^) *m*/*z*: 468.93 [Cp_2_W(thioethylmaltolato)]^+^. IR: ν = 3126 w (ν_C–H_, Cp), 1571 m (ν_C=S_), 1499, 1408 m (ν_C=C_), 815 s (ν_C–H_) cm^−1^.

### 2.3. Cell Lines and Culture Conditions

CH1/PA-1 (ovarian teratocarcinoma), SW480 (colon carcinoma) and A549 (non-small cell lung cancer) cells were maintained as described in the reference [41]. IMR-90 human fetal lung fibroblasts (from the European Collection of Authenticated Cell Cultures, Porton Down, Salisbury, UK) were cultured in Stable Cell^TM^ DMEM/F12 supplemented with 0.01 mg/mL human recombinant insulin (both purchased from Sigma-Aldrich) and 10% fetal bovine serum (FBS, BioWest, Nuaillé, France). Human colon carcinoma cell lines HCT116 and HT29 (both kindly provided by the Institute of Cancer Research, Department of Medicine I, Medical University of Vienna, Austria) were cultured in McCoy’s 5a medium supplemented with 4 mM L-glutamine (both purchased from Sigma-Aldrich) and 10% FBS. MCF-7 human breast adenocarcinoma cells (kindly provided by M. Ogris, Department of Pharmaceutical Sciences, University of Vienna, Austria) were maintained in DMEM (high glucose) supplemented with 4 mM L-glutamine, 0.1 mg/mL human insulin (all purchased from Sigma-Aldrich) and 10% FBS. All cell lines were grown as monolayers in 75 cm^2^ culture flasks (CytoOne, Starlab, Hamburg, Germany) at 37 °C in a humidified atmosphere containing 5% CO_2_.

### 2.4. Spheroid Formation and Growth

For growing multicellular tumor spheroids, 400 HCT116, 400 HT29 or 800 MCF-7 cells per well were plated into round-bottom ultra-low attachment 96-well plates (Corning, Glendale, AZ, USA) in the media described above and incubated at 37 °C for 96 h. Spheroids were treated with 10 µM of the two most cytotoxic compounds **3** and **4** for another 96 h. The diameters of the spheroids were measured with Cell^F software under a CKX41 Olympus microscope before treatment and in constant daily intervals over the 96 h treatment period. For statistical analysis the *t*-test with Welsh’s correction was implemented in GraphPad Prism software (Version 6.01).

### 2.5. Cell Viability (MTT Assay)

Exponentially growing cells were plated into 96-well microculture plates (CytoOne, tissue culture treated, Starlab, Hamburg, Germany) in densities of 1 × 10^3^ CH1/PA-1, 2 × 10^3^ SW480, 3 × 10^3^ A549 and 7 × 10^3^ IMR-90 cells per well (each in 100 µL). After a 24 h preincubation, drugs were added in serial dilution to give a final volume of 200 µL/well and exposed for 96 h. At the end of the exposure, the drug solutions were replaced by 100 µL RPMI 1640/MTT mixture (7:1 ratio). MTT (3-(4,5-dimethylthiazol-2-yl)-2,5-diphenyl-2H-tetrazolium bromide), (Acros Organics, Geel, Belgium) solution was prepared in phosphate-buffered saline (5 mg/mL) and mixed with RPMI1640 medium supplemented with 4 mM L-glutamine (both from Sigma-Aldrich) and 10% FBS. After 4 h incubation, the formazan crystals were solubilized in 150 µL of DMSO per well. Absorbance was measured at 550 nm (and at 690 nm as a reference) with a microplate reader (ELx808, BioTek, Winoosky, VT, USA) and Gen5^TM^ 3.08 software (BioTek). The 50% inhibitory concentrations (IC_50_) relative to untreated controls were interpolated from concentration-effect curves. At least three biologically and technically independent experiments were performed.

### 2.6. Cell Viability (Resazurin Assay)

For the resazurin assay in monolayer cultures, cells were seeded into 96-well microculture plates (CytoOne, tissue culture treated, Starlab, Hamburg, Germany) in densities of 2 × 10^3^ HCT116, 2 × 10^3^ HT29 and 4 × 10^3^ MCF-7 cells per well (each in 100 µL). For tests in 3D cultures, spheroids were grown as described above. After 24 h (for monolayers) or 4 days (for spheroids) the test compounds in the appropriate medium were added in serial dilution to the plates to obtain a total volume of 200 µL/well. Wells without cells, containing medium and resazurin (Alfa Aesar, Thermo Fisher, Kandel, Germany) solution were used as blanks. After 96 h exposure 20 µL of the resazurin solution (440 µM) were added to each well, followed by a 4 h (2D) or 12 h (3D) incubation period. Resorufin fluorescence was quantified at 530 nm (and a reference wavelength of 620 nm) by using a microplate reader (Synergy HT, BioTek) with Gen5^TM^ 3.10 software (BioTek).

### 2.7. Apoptosis Studies

Apoptosis and necrosis induction upon drug treatment was analyzed by double staining with FITC-conjugated annexin V (eBioscience, San Diego, CA, USA) and propidium iodide (PI, 1.0 mg/mL, Sigma-Aldrich) via flow cytometry. In brief, HCT116 and HT29 cells (7 × 10^4^ cells/well) as well as MCF-7 cells (9 × 10^4^ cells/well) were seeded into 24-well plates in 600 µL MEM and incubated overnight for adherence. After resuming exponential growth, cells were exposed to different concentrations of the test compounds for further 24 h. Positives controls were treated with KP1998 (compound 7 in reference [42]). Following treatment, the drug-containing medium was collected, cells were washed once with PBS and harvested. After trypsinization, the cell suspension was added to the pre-collected medium and cells were pelleted by centrifugation (300× *g*, 3 min). The supernatant was removed, and cells were stained with FITC-conjugated annexin V (0.4 µg/mL) in binding buffer (10 mM HEPES/NaOH pH 7.4, 140 mM NaCl, 2.5 mM CaCl_2_) and incubated at 37 °C for 15 min in the dark. Propidium iodide (1.6 µg/mL) was added shortly before flow cytometry analysis. Samples were analyzed with a Millipore Guava easyCyteTM 8HT flow cytometer (Merck Millipore, Burlington, MA, USA) with InCyte software. Five thousand events were collected for each experiment, and at least three independent experiments were performed. The recorded results were analyzed with FlowJo software 10.6.1 (TreeStar, Ashland, OR, USA).

### 2.8. Electrophoretic dsDNA Plasmid Assay

An amount of 400 ng of the pUC19 plasmid (2686 bp, New England Biolabs, Ipswich, MA, USA) was incubated with the test compounds (50 µM). Stock solutions of the test compounds were prepared in MilliQ water. The samples were incubated for different intervals (15 min to 6 h) with continuous shaking at 37 °C. Following the incubation time, 4 µL aliquots of 6 × DNA loading dye (Thermo Fisher, Kandel, Germany) were added to the 20 µL DNA samples. Electrophoresis was performed in a 1% agarose gel in 1 × TBE buffer, started at 60 V for 5 min and continued at 120 V for 90 min. Ethidium bromide (EtBr, Serva Electrophoresis GmbH, Heidelberg, Germany) staining was conducted in 1 × TBE (0.75 µg/mL) for 20 min. Images were taken by the GelDoc Imaging System Fusion F×7 (Vilber Lourmat, Eberhardzell, Germany). For quantification ImageJ/Fiji1.46 was used.

### 2.9. Reactive Oxygen Species (ROS) Detection

Reactive oxygen species (ROS) generation was measured by means of the DCFH-DA assay. HCT116 and HT29 cells (2.5 × 10^4^ cells/well) as well as MCF-7 cells (3.5 × 10^4^ cells/well) were seeded into 96-well plates (100 µL/well) and incubated overnight for attachment. On the following day, cells were washed with 200 µL Hanks’ balanced salt solution (HBSS, Sigma-Aldrich, containing 1% FBS) and stained with 100 µL of 25 µM DCFH-DA (2′,7′-dichlorofluorescein diacetate, Sigma-Aldrich) in HBSS (containing 1% FBS) for 45 min at 37 °C in the dark. After the incubation period, cells were washed with 200 µL HBSS (1% FBS) and loaded with compounds at concentrations of 2 µM, 20 µM, 100 µM and 200 µM in 200 µL phenol-red-free MEM (1% FBS) in triplicates. As positive controls, cells were treated with 200 µM and 400 µM tert-butylhydroperoxide (Sigma-Aldrich). Wells containing only phenol-red-free medium with 1% FBS were used as blanks, and non-drug treated, cell containing wells as reference values. Fluorescence (excitation: 485/20 nm, emission: 516/20 nm) was recorded in 10 min intervals with the Synergy HT reader (BioTek) over 2 h.

To verify interactions of complex **4** with the fluorescence of DCF (2′,7′-dichlorofluorescein, Sigma-Aldrich), a fluorescence quenching assay was performed. For this purpose, DCF was dissolved in abs. ethanol (Sigma-Aldrich), diluted in phenol-red-free MEM (1% FBS) to 5 µM and complex **4** dissolved in DMF, diluted to 20 and 200 µM in the same medium. Furthermore, two combinations of 5 µM DCF with 20 and 200 µM of complex **4** were prepared. The fluorescence intensity of the substances alone and their combinations were measured (excitation: 485/20 nm, emission: 516/20 nm) in 10 min intervals with the Synergy HT reader (BioTek) over 2 h.

### 2.10. LA-ICP-TOF-MS Analysis

Totals of 400 HCT116, 400 HT29 and 800 MCF-7 cells per well were seeded into round-bottom ultra-low attachment 96-well plates (Glendale, AZ, USA) for production of spheroids within 4 days. On the fifth day, spheroids were treated with the test compounds at concentrations based on their IC_50_ values (according to the 3D resazurin assay). After 72 h spheroids were collected, embedded in TissueTek (Sakura, Tokyo, Japan) and stored at −80 °C until further processing. Samples were cut with a Cryostat CM3050S (Leica Biosystems, Nussloch, Germany) in 5 µm thin sections and placed on Superfrost slides (Thermo Fisher Scientific, Kandel, Germany). An Iridia 193 nm excimer laser ablation system (Teledyne Photon Machines, Bozeman, MT, USA) was coupled to an icpTOF 2R (TOFWERK AG, Thun, Switzerland) via an aerosol rapid introduction system (ARIS). The LA and ICP-TOF-MS settings were optimized on a daily basis using NIST SRM612 glass certified reference material (National Institute for Standards and Technology, Gaithersburg, MD, USA). Laser ablation sampling was performed in NoGas mode, in fixed dosage mode 2, at a repetition rate of 200 Hz, a fluence of 0.7 J cm^2^ and using a 5 µm × 5 µm square spot. The line scans overlapped one another by 2.5 µm.

### 2.11. Immunofluorescence Staining

Spheroids were grown and cryosectioned, as described in the section above. For immunofluorescence staining the samples were fixed with 4% PFA (in PBS) for 15 min. The slides were washed with PBS before permeabilization with 0.1% Triton-X (Sigma-Aldrich, Steinheim, Germany) for 10 min. After three times washing with PBS the samples were stained for 45 min with 10% goat serum (G9023, Sigma-Aldrich) und 5% BSA for avoiding non-specific antibody binding. After one washing step a primary antibody against Ki67 (#9129, Cell Signaling Technology) was applied and samples incubated overnight at 4 °C. On the next day, slides were washed with PBS and incubated with goat anti-rabbit Alexa Fluor 594 (#8889 Cell Signaling Technology) for 1 h at room temperature in the dark, followed by two washing steps with PBS. Slides were mounted with ProlonGold Antifade Reagent with DAPI (Invitrogen, Thermo Fisher) and analyzed with a confocal laser scanning microscope Zeiss LSM800 (Zeiss, Jena, Germany). Images were processed with Zen software (Zeiss). For fluorescence signal intensities data extraction ImageJ/Fiji1.46 was used. For quantification, the maximum signal intensity of the KI67^+^ cells was evaluated relative to the untreated control. For statistical analysis the *t*-test with Welsh’s correction was implemented in GraphPad Prism software (Version 6.01).

## 3. Results and Discussion

### 3.1. Synthesis and Characterisation

**Scheme 1:**
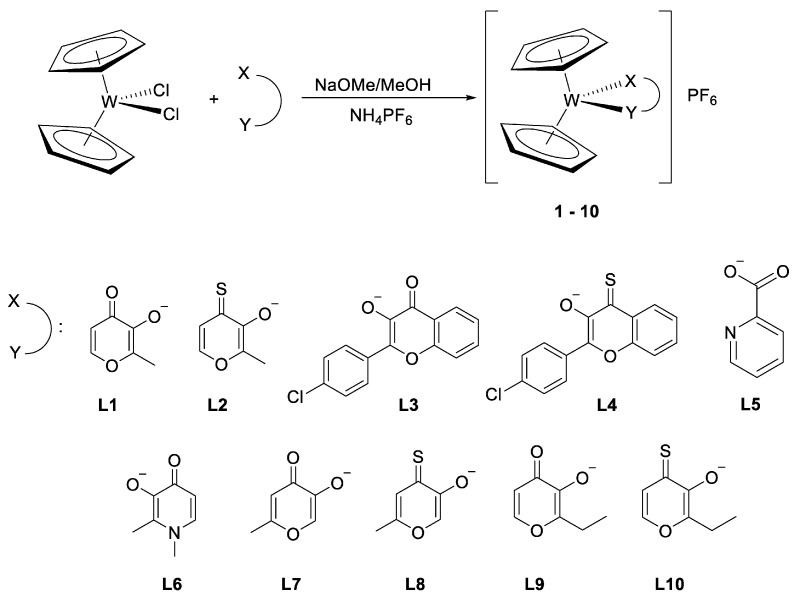
Synthesis of complexes **1**–**10**.

The syntheses of the (*S*,*O*–) chelates (**L2**, **L8**, **L10**) using Lawesson’s reagent and ^1^H-NMR spectra can be found in the supporting information (Figures S1–S11 and see Scheme 1 for structures of ligands and complexes). Complexes **1**–**10** were obtained by exchanging the chlorido ligands of the Cp_2_WCl_2_ starting material through a standard complexation method, resulting in positively charged complexes. All reactions were performed under inert conditions under an argon atmosphere. Anion exchange reactions, however, were carried out open to air. The general synthetic procedure involved weighing the starting material Cp_2_WCl_2_, the corresponding ligand **L1**–**L10** and the base sodium methoxide in a glove-box under an argon atmosphere, adding dry methanol and stirring for 4–72 h at room temperature or under reflux. Unreacted materials, if present, were removed by filtration. Afterwards, ammonium or sodium hexafluorophosphate (PF_6_^−^) was added and the solution stirred again for 2–4 h. The formed precipitate was filtered off and washed with methanol or extracted using dichloromethane, yielding **1**–**10** in low to moderate yields: 27–62%. Complex **1A** also contains the maltolato ligand but has a chloride as counterion, it did not undergo the PF_6_^−^ exchange reaction described above (for comparison purposes). All compounds are soluble in chlorinated solvents, acetone, ethyl acetate and sparingly soluble in water, methanol and ethanol. The solids are highly stable in air; however, storage for longer periods under argon is recommended.

The IR spectra of complexes **1**–**10** showed the typical C–H stretch for the Cp moieties (3130–3120 cm^−1^) as well as the characteristic ν_C=O_ and ν_C=C_ bands (1610–1505 and 1500–1420 cm^−1^, respectively) for coordinated complexes. Due to the uncoordinated C=O of compound **5**, the ν_C=O_ stretch appears at higher wavenumbers (1684 and 1610 cm^−1^), which is typical. In addition, ν_C–H_ bending is observed in the common wavenumbers (ca. 820 cm^−1^) [43]. These values are comparable with the tungstenocenes found in the literature [16].

An advantage of bent metallocenes is that the ^1^H-NMR spectra of each complex (Figures S1–S11) shows just one signal for both cyclopentadienyl rings, since the rings are symmetrically coordinated to tungsten in a η^5^ manner and the bidentate ligand is in an ancillary position, located in the plane bisecting the Cp-W-Cp. All NMR spectra were recorded in d_6_-DMSO and all peaks in the ^1^H and ^13^C have been fully assigned. The signal of the Cp ring in complexes **1**–**10** shifts downfield (δ = 6.06–5.70 ppm) in the ^1^H-NMR compared with tungstenocene dichloride (δ = 5.63 ppm). Signals for the ^1^H-NMR spectrum of compound **1** show exactly the same chemical shifts as the chloride analogue reported in the literature [16]. All (*S*,*O*–) chelates show an upfield shift in the Cp signal of 0.20 ppm compared to their (*O*,*O*–) analogues.

Compounds **1**–**10** were dissolved in 1% methanol/water and identified by ESI-MS measurements, where peaks for the [Cp_2_W(**L**)]^+^ fragment were observed in the positive mode. Characteristic tungsten isotope patterns were also found for all complexes. The theoretical and experimental *m*/*z* values are listed in Table S1.

### 3.2. X-ray Diffractometry

**Figure 2:**
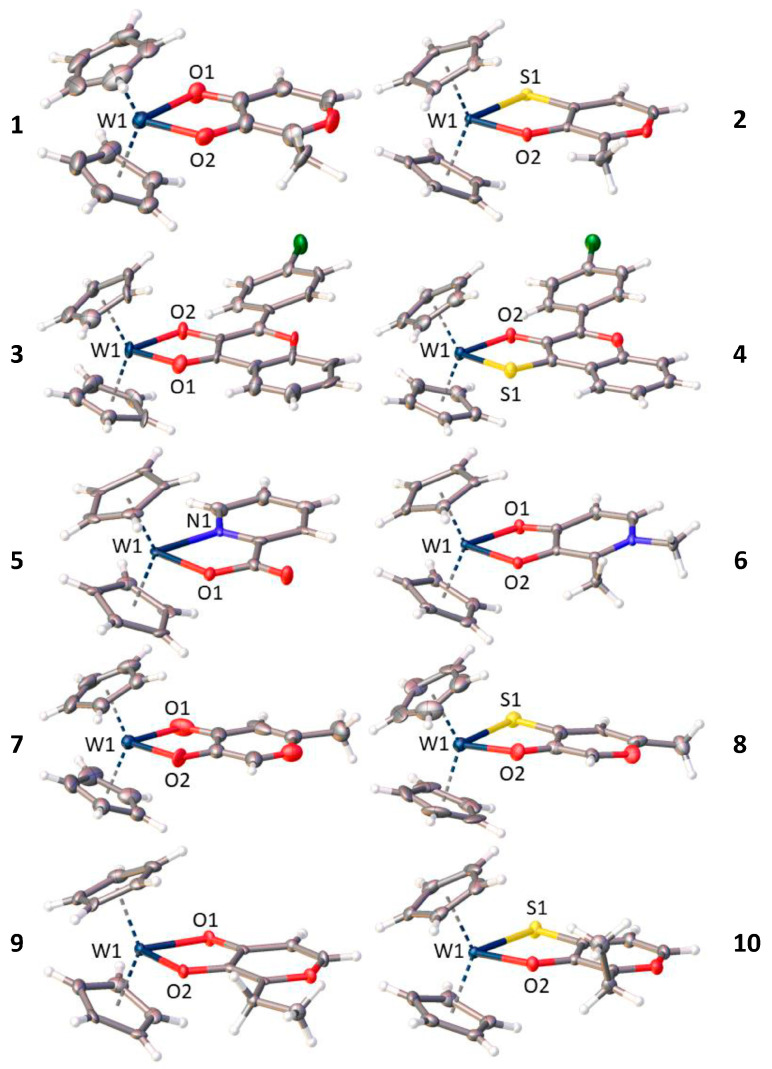
Crystal structures of compounds **1**–**10**, drawn with 50% displacement ellipsoids.

X-ray suitable crystals of all compounds were obtained by slow diffusion of *n*-hexane in acetone (compounds **1A**, **3**–**6**, and **8**–**10**) or ethanol (compounds **2** and **7**) solutions (Figure 2). Compound **1A** (with a chloride counterion instead of the hexafluorophosphate) crystallized in the orthorhombic *Cmca* space group, compounds **2** and **9** in the orthorhombic *Pnma* space group, compounds **3**, **4** in the monoclinic *P2_1_*/*n* space group and **8** in the monoclinic *P2_1_*/*c* space group, compounds **5** and **6** in the orthorhombic *Pca2_1_* and *Pna2_1_* space groups, respectively, compound **7** in the orthorhombic *Pmn2_1_* space group, and compound **10** in the *P-1* triclinic space group. Unfortunately, the structure of **1A** contains a disordered maltolato ligand (the two disordered states of the maltolato moiety rotate by 180°) and further discussions of this structure are not useful. We obtained more than one measurable crystal of **1A** but in each measurement the disorder appeared. The bond lengths of the coordinating carbonyl or thione in position 1 are, as expected, very distinctive. W–S bonds are the longest, with values in the range 2.430–2.454 Å, whereas the W–O bonds are the shortest, in the range of 2.067–2.127 Å. In between lies the W–N bond, at 2.157 Å (see Table 1 for selected bond lengths). The bond lengths between the W and the Cp-centroids are found to be the in same range, from 1.964 to 1.983 Å. The ring slippage is not insignificant (0.106–0.173 Å) and indicates a strain on the Cp moieties most likely resulting from their interactions with each other rather than interactions with the bidentate ligands since no discernable trend could be identified. The W–O2 bond lengths in complexes **2**–**10** do not differ much from each other and are found to be in a narrow range (2.067–2.102 Å).

Crystal data, data collection parameters and structure refinement details are given in Tables S2–S22.

### 3.3. Cyclic Voltammetry

The electrochemical behavior of all complexes and the precursor Cp_2_WCl_2_ was studied by cyclic voltammetry with a scan rate of 200 mV/s, from −0.5 to +1.2 or +1.5 V. Measurements were performed in triplicate. Due to low water solubility of compounds **1**–**10** acetonitrile (MeCN) was utilized in order to reach the 2 mM concentration. All complexes exhibit a reversible one-electron process, W^IV^ to W^V^.

**Figure 3:**
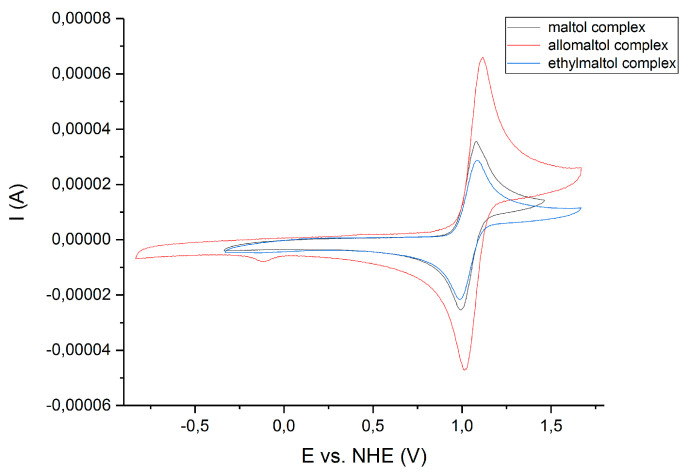
Cyclic voltammogram of the maltol derivatives ((*O*,*O*–) chelates), compounds **1**, **7** and **9**, in MeCN referenced to the NHE.

The oxidation of W^IV^ to W^V^ for complexes **1**–**10** takes place in the potential range 0.885 to 1.330 V vs. Normal Hydrogen Electrode (NHE) measured in acetonitrile, whereas for Cp_2_WCl_2_ it occurs at 0.648 V (Table 2). Hence, the coordination of the bidentate ligands increases the redox stability of the complexes, making them more difficult to oxidize. Due to solvent effects, the values obtained for complexes **1**–**10** cannot be distinctively compared with physiological significant potentials [40]. The physiological significant potential in the cell is delimited by NADP^+^ at −0.320 V (NADP^+^ + H^+^ + 2e^−^ → NADPH) and by oxygen at +0.820 V (O_2_ + 4 H^+^ + 4e^−^ → 2 H_2_O). All compounds, except for possibly compound **6** because of its significantly lower oxidation potential of 0.886 V, will most likely, but we cannot say absolutely, show redox activity outside the physiological region. The donor atoms directly influence the redox potential; compounds **1**, **7** and **9**, bearing the (*O,O*–) donors (Figure 3, for the CV of maltol, allomaltol and ethylmaltol complexes and the supporting information Figures S12–S16 for all other compounds), show lower redox potentials (1.037–1.071 V) than their (*S,O*–) counterparts (1.047–1.080 V), whereas the (*N,O*–) donor-atom derivative compound **5** exhibits the highest oxidation potential (1.330 V). Furthermore, the molybdenum counterpart of compound **5** also showed a considerably higher redox potential when compared to the precursor and to other molybdenocene derivatives [21]. The lowest oxidation potential, as already mentioned, belongs to compound **6**, the deferiprone derivative (0.885 V). Compounds **1** and **2**, bearing the maltol and thiomaltol ligands, are more easily oxidized than compounds **3** and **4**, which bear the (*O,O*–) and (*S,O*–) flavone moieties, respectively, and exhibit higher oxidation potentials. The shifting to higher redox potentials upon coordination to the bidentate ligands has also been observed in the literature; previous studies with (*O,O*–) donor ligand scaffolds reveal that upon coordination of the (*O,O*–) moiety the redox potential increases by 0.35–0.40 V [16]. As we did not observe any color changes during the CV experiments, we are confident that the MeCN does not interact with the complexes. In order to confirm this, we recorded an ^1^H NMR in MeCN–d_3_ of compound **3**, over the time span of a cyclic voltammetry experiment, and no change in the NMR spectra was observed over 30 min (Figure S17).

### 3.4. Aqueous Solubility and Stability Measurements

In order to examine the stability of the compounds in aqueous solution, UV/Vis measurements were performed every hour during 24 h at 293 K. Solutions of compounds **1**–**10** were prepared in PBS (10%) from a previously prepared DMSO stock solution of each compound. The total concentration of DMSO was 1 vol% for the final complex solutions of **1**–**10**.

All complexes were found to be stable in PBS over 24 h, as the peak maxima (λ_max_) do not shift. However, compounds **3**, **4**, **7**, **8** and **10** slowly precipitate out of the solution over time (Figures S18–S27). Precipitation, however, was not a problem when working at low concentrations in the in vitro experiments. The wavelength of the peak maxima and molar extinction coefficients are reported in the supplementary information Table S23.

Overall, compounds **1**–**10** showed sufficient aqueous solubility and stability for further biological investigations.

### 3.5. Cytotoxic Activity In Vitro (MTT Assay)

The cytotoxic activities in vitro of compounds **1**–**10** as well as the starting material Cp_2_WCl_2_ and compound **1A** (cation of compound **1** with a chloride as counter ion) were investigated by means of the colorimetric MTT assay in the human cancer cell lines A549 (non-small cell lung carcinoma), SW480 (colon carcinoma), CH1/PA-1 (ovarian teratocarcinoma) and in human lung fibroblasts IMR-90. Compounds **2**, **3**, **4**, **10** and Cp_2_WCl_2_ show a typical pattern of antiproliferative activities in cancer cell lines, with the highest activity in the cell line CH1/PA-1, which is a broadly chemosensitive cell line [26], moderate activity in the cell line SW480 and the lowest activity in the intrinsically multi-drug resistant A549 cell line (Figures S28 and S29) [45]. Furthermore, compounds **2**, **3**, **4** and **10** show lower cytotoxicity in IMR-90 fibroblasts, which suggests a capacity of these drugs to selectively eliminate malignant cells (Figures S28 and S29). Remarkably, complex **2**, containing the (*S*,*O*–) donor thiomaltol ligand, shows a moderate non-selective activity in all tested cancer cell lines, which is promising with regard to overcoming multidrug resistance. Compounds **3** and **4**, bearing a flavone and a thioflavone ligand, respectively, show the most promising IC_50_ values, reaching the low micromolar range in the cell lines CH1/PA-1 and SW480, and compound **4** even in the A549 cell line. The high in vitro anticancer activity of transition metal complexes bearing flavones and their derivatives is known in the literature [37,46,47]. The thioethylmaltol (**10**) derivative shows an enhanced potency in all three cell lines. In the case of the maltolato complexes **1**, bearing PF_6_^−^ as the anion, and **1A**, bearing Cl^−^ as the anion, no activity was observed in the SW480 and A549 cell lines.

The in vitro anticancer activity data of the analogous molybdenocene derivatives synthesized by our group have also been included in Table 3 [21]. Compound **2** shows greater activity in two cells lines (SW480 and A549) than its molybdenum counterpart; however, the IC_50_ values in the CH1/PA-1 cell line are in the same range. Compounds **3** and **4**, bearing the (thio)flavone ligands, are the most active of the series, mirroring the molybdenum (thio)flavone complexes, which show a very similar in vitro anticancer activity and were also the most active compounds in their series. The picolinic acid (**5**) and deferiprone (**6**) derivatives show negligible activity, while the allomaltol (**7**), thioallomaltol (**8**) and ethylmaltol (**9**) derivatives show similar activities as complex **1** (bearing a maltol ligand). In general, the (*S*,*O*–) chelates are more active than their (*O*,*O*–) counterparts and the tungstenocenes are more active than the molybdenocenes with some exceptions. According to Meléndez et al., the enhancement in cytotoxicity of the tungstenocene derivatives is due to their lower oxidation potentials when compared with their molybdenum analogs, resulting in higher redox activity under physiological conditions [16]. Even though the in vitro anticancer activity of Cp_2_WCl_2_ seems to be quite promising (IC_50_ values of 38 µM in SW480 and 2.4 µM in CH1/PA-1 cells), this compound is not stable enough when exposed to air and it is known to decompose in the physiological environment in less than 3 h, making the active species unidentifiable [16].

### 3.6. Drug Sensitivity in 2D and 3D Cultures, Effects on Growth and Morphology

The activity of compounds **1A**, **2**–**10** and Cp_2_WCl_2_ was compared in conventional 2D monolayer and 3D multicellular tumor spheroid models. In publications of Meléndez et al., new tungstenocenes and molybdenocenes were studied for their cytotoxic activity in 2D-cultured cell lines such as HT29 (colorectal adenocarcinoma) and MCF-7 (breast adenocarcinoma) [16,22]. In addition to these two cell lines, HCT116 (colorectal adenocarcinoma) cells were selected for analyzing drug sensitivity, effects on growth and morphology, as well as apoptosis and ROS induction by our novel tungstenocenes. To overcome the limitations of monolayer-based assays, 3D cell cultures have been chosen to improve the screening of anticancer drug candidates by increasing the dimensionality with all its implications [48,49,50,51,52]. All three cell lines are applicable for 3D cell culture, which more realistically mimics the biochemical and biophysical environment of solid tumors. For determining the activity of the new tungstenocenes in 2D and 3D cell cultures the resazurin assay was chosen, where metabolically active cells reduce the blue non-fluorescent resazurin to red, highly fluorescent resorufin, enabling the quantification of cell viability by measurement of relative fluorescent units (RFU) [53].

Compounds **3** and **4**, bearing (thio)flavone as chelating ligand, must be highlighted based on their strong antiproliferative activity. Both complexes showed markedly increased activity in the low micromolar range in both monolayers and spheroid cultures in the tested cell lines. These results correlate well with the MTT-based results, where compounds **3** and **4** were found the most cytotoxic ones in CH1/PA-1, SW480 and A549 cell lines (Table 3 and Table 4). The ratios of IC_50_ values in 3D over 2D models were calculated, where compounds **3** and **4** displayed their lowest 3D/2D ratios (2.3 and 2.5, respectively) in HCT116 cells and their highest ratios in MCF-7 cells (7.0) (Table S24). Thiomaltol complex **2** showed nearly equal but modest activity in 3D and 2D (ratio 1.6) in HCT116 cells; furthermore, an IC_50_ was reached in MCF-7 monolayers (66 ± 12 µM), but otherwise IC_50_ values were higher than the maximum tested concentration (>200 µM). Complex **10** demonstrated activity in monolayers of the two cell lines, whereas in 3D models IC_50_ values could only be reached in HCT116 spheroids, but with a remarkably low ratio between 3D and 2D (1.6) (Table S24). Concentration–effect curves for compounds **2**–**4** and **10** are depicted in Figures S30–S32. The other tested tungstenocenes showed nearly no cytotoxicity in HCT116, HT29 and MCF-7 cells. Obviously, the leaving groups play a crucial role for cytotoxic activity. Generally, compounds with enhanced cytotoxicity bear (*S,O*–) bidentates in the ancillary position, whereas complex **3** bearing a (*O,O*–) chelating flavone ligand tends to be similarly cytotoxic as complex **4** in 2D and 3D cell culture models.

**Figure 4:**
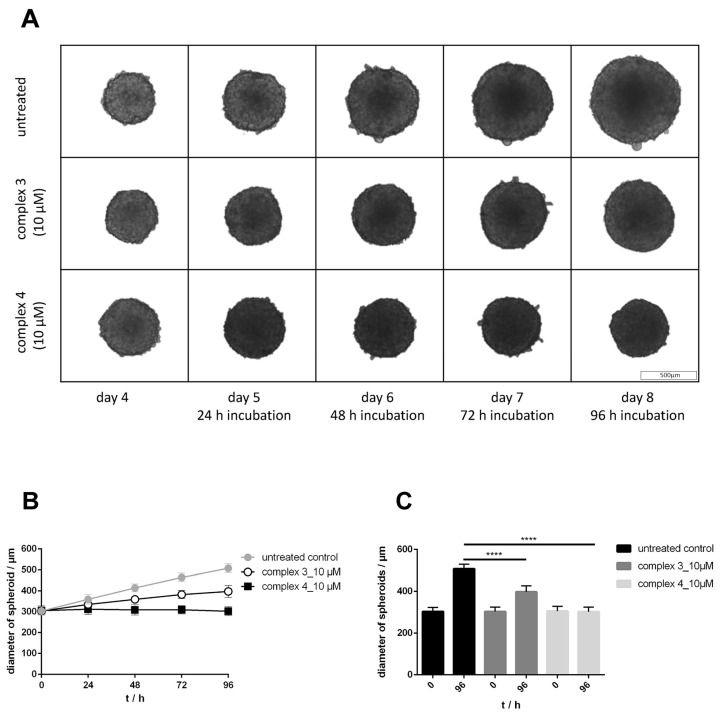
(**A**) Time-dependent growth of HCT116 spheroids upon treatment with complexes **3** and **4** (both 10 µM) and untreated control, detected for 96 h (treatment started on day 4 after seeding). Images represent one captured spheroid out of three experiments. Scale bar = 500 µm (**B**) Growth curves for spheroids of HCT116 cells treated with complexes **3** and **4** (both 10 µM) and untreated control. Measurements started on day 4 (after seeding) with spheroid diameters of 304 ± 1 µm and were continued every 24 h for additional 4 days. (**C**) Spheroids were initiated on day 0, treated with complexes **3** and **4** (both 10 µM) on day 4, diameters of spheroids were obtained at the beginning of the treatment (0 h) and at the end (96 h). Unpaired *t*-test with Welch’s correction (n = 72) was performed for statistical evaluation (**** *p* < 0.0001).

The two most cytotoxic compounds **3** and **4** bearing (thio)flavone ligands, were investigated for their impact on long-term proliferation capacity. For better comparison, a concentration of 10 µM was used for both complexes in all three cell lines. Spheroids were grown for 4 days (to average diameters of 304 ± 1 µm (HCT116), 334 ± 6 µm (HT29) and 331 ± 7 µm (MCF-7)) and then treated with the tungstenocenes for a further 4 days. Cell growth and morphology was monitored every day and the respective diameters were determined (Figure 4 and Figures S33–S36). Both compounds were capable of inhibiting spheroid growth with similar magnitude. HCT116 spheroids treated with complex **3** showed a markedly reduced growth (*p* < 0.0001) compared to the untreated control, and complex **4** completely stopped spheroid growth (*p* < 0.0001) already after 24 h incubation (Figure 4). In the other two cell lines, HT29 and MCF-7, both complexes were able to completely stop spheroid growth (in all cases *p* < 0.0001) already after 24 h treatment (Figures S33–S36). When comparing the morphologies of the three different spheroid models, the compactness and the nice spherical shape of HCT116 and HT29 could be noticed, while MCF-7 demonstrated a less even outer margin. Remarkably, the structural integrity in the proliferating zone of HT29 spheroids under treatment with complex **4** was obviously lower, already after 48 h incubation.

**Figure 5:**
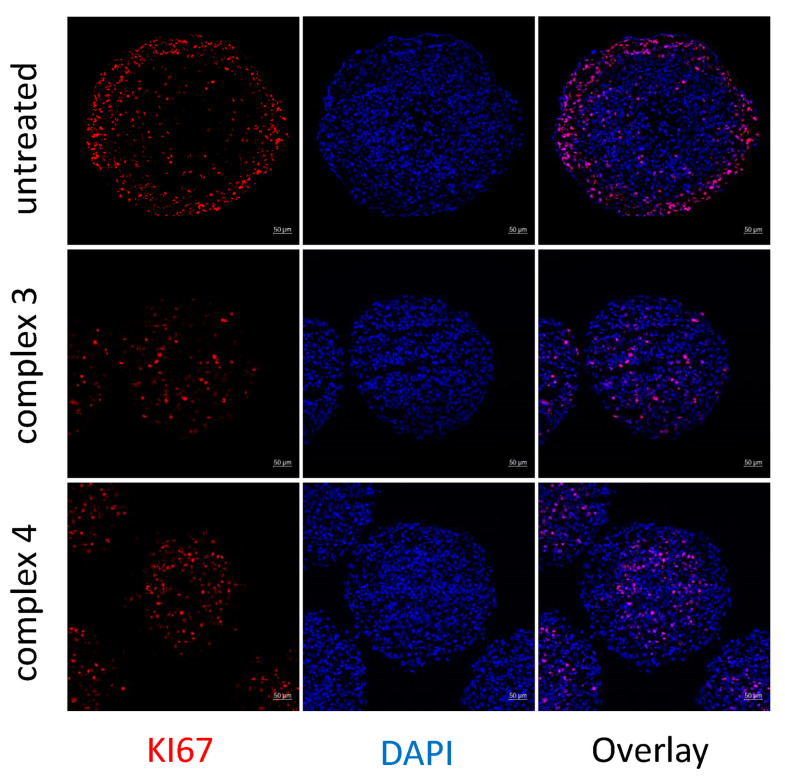
Confocal laser scanning fluorescence microscopy of paraformaldehyde (PFA)-fixed, 5 µm cryo-sectioned HCT116 spheroids, untreated or treated with complex **3** (20 µM) and complex **4** (15 µM) for 72 h. Detection of KI67 (proliferation marker, red) and DAPI-staining (nuclei, blue), and co-localized fluorescence (magenta). Scale bar = 50 µm.

Subsequently, the tungstenocene-induced inhibition of cell proliferation was investigated via the cell cycle regulated protein KI67, which is widely used as a proliferation marker [54,55,56]. HCT116 spheroids were grown for 4 days and exposed to complexes **3** and **4** (both 10 µM) for 72 h. The treated spheroids were cryo-sectioned followed by KI67 immunofluorescence labelling and confocal laser scanning microscopy. The low number of KI67^+^ cells indicated high antiproliferative activity of the treated sample. In the untreated control, the vast majority of KI67^+^ cells were detected at the periphery of the multicellular tumor spheroid (MTS) section. In total 38.1 ± 7.3% of cells in the control were found to be in proliferating state, in contrast to the exposed samples, where the numbers of proliferating cells decreased significantly (15.8 ± 2.8% for complex **3** and 19.1 ± 1.3% for complex **4**) (Figure 5, Table S25). While it is apparent that tungstenocene treatment inhibits the proliferation of cells in the outermost layers of the spheroid (Figure 5) Ki67^+^ cells could be observed in deeper regions, showing that proliferation persists in the inner parts of the MTS. A 2.4- and 2-fold reduction in number of KI67^+^ cells was quantified relative to untreated control, for complex **3** and complex **4**, respectively (in both cases *p* < 0.05 (Figure S37)). Interestingly, complex **4** was observed to efficiently inhibit the proliferation of the cells on the edge but barely in the inner of the spheroid (Figure 5). These findings might reflect a limited uptake of complex **4** into the deeper zones of HCT116 spheroids; for this purpose, W distribution was investigated in cryo-sectioned HCT116 spheroids via LA-ICP-TOF-MS (laser ablation (LA) in combination with inductively coupled plasma time-of-flight mass spectrometry (ICP-TOF-MS)).

### 3.7. W Distribution in HCT116 Tumor Spheroids

**Figure 6:**
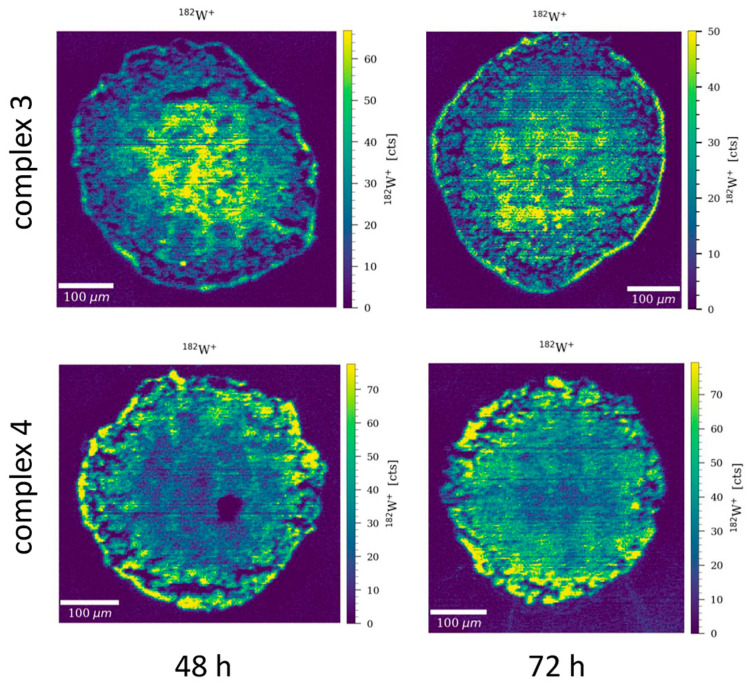
Qualitative images obtained by LA-ICP-TOF-MS for W distribution in HCT116 spheroids after treatment with complex **3** (10 µM) and complex **4** (10 µM) for 48 and 72 h. High-resolution laser ablation images were measured with a 5 µm square mask, double oversampling and a repetition rate of 200 Hz. (Scale bar = 100 µm).

Multicellular HCT116 spheroids were treated with complexes **3** and **4** at sub cytotoxic concentration (10 µM) for analyzing the distribution of W (Figure 6) and qualifying a panel of other abundant elements by using high-resolution laser ablation (LA) in combination with inductively coupled plasma time-of-flight mass spectrometry (ICP-TOF-MS). Signal intensity of elements (isotopes) of biological relevance were measured after 48 and 72 h incubation with the two selected tungstenocenes. Flavone complex **3** yielded the highest tungsten accumulation in the inner layers of multicellular tumor spheroids (indicated by yellow color in Figure 6). These findings correlate well with proliferation marker (KI67) analysis (Figure 5), where a significantly lower level of KI67 signal was detected in the outer rim of the spheroid section, illustrating a clear decline in proliferative activity. The capacity of deep penetration of complex **3** indicates an increased chance of damaging non-proliferating tumor cells in the quiescent zone and in the spheroid core. Remarkably, thioflavone complex **4** displayed a tungsten enrichment predominantly in the proliferating zone and slighter signals in the quiescent zone or in the inner core. This suggests that the actively dividing cancer cells in the periphery of MTS accumulate tungsten to a high extent. Thus, the W distribution pattern of the two most cytotoxic tungstenocenes differs extremely, although these two complexes only differ in the presence of W–S or W–O bonds. These findings also suggest that the flavone ligand is more favorable for entering the spheroids and reaching the inner core, whereas it induces less cytotoxicity in all three tested cell lines than the thioflavone counterpart. In addition to tungsten (^182^W^+^), qualitative bioimaging was evaluated of ^23^Na^+^, ^24^Mg^+^, ^31^P^+^ and ^66^Zn^+^ after 48- and 72-h incubation at different concentrations, at 10 µM and additionally roughly at IC_50_ values (15 µM and 20 µM) for each compound. A pronounced accumulation of ^23^Na^+^, ^24^Mg^+^ and ^31^P^+^ could be observed in the outer rim of the selected spheroid sections, whereas the accumulation of ^66^Zn^+^ is negligible (Figures S38–S40).

### 3.8. Oxidative Stress Studies

**Figure 7:**
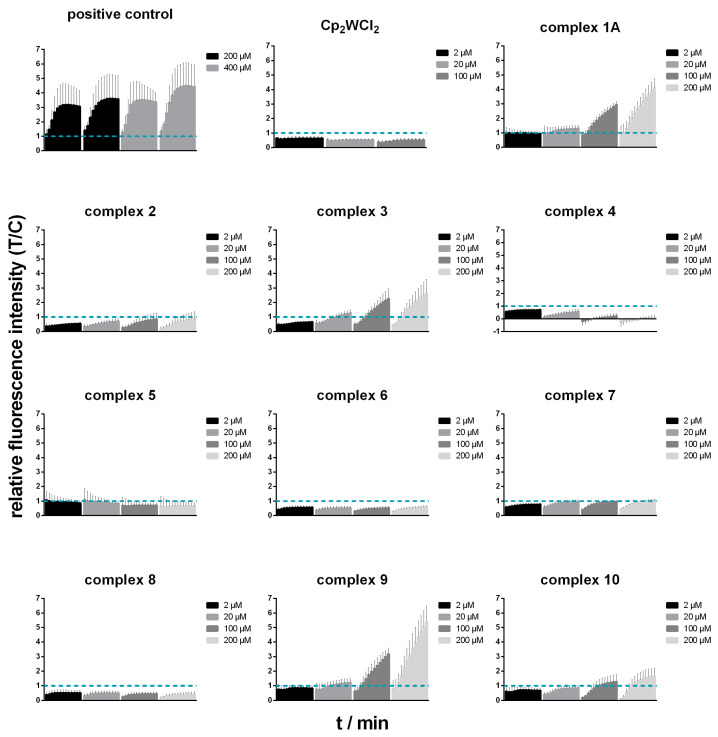
Time- and concentration-dependent intracellular reactive oxygen species (ROS) generation in HCT116 cells. Cells were treated with tungstenocenes (2, 20, 100 and 200 µM) and tert-butyl-peroxide (200 and 400 µM) as positive control. Fluorescence intensities of DCF indicating ROS levels (relative to the untreated control) were measured with a spectrophotometer. Data from three independent experiments with n = 3 wells per measurement are given as means ± standard deviations. The dashed, blue line indicates the ROS level of untreated controls to which the other values were normalized.

Intracellular reactive oxygen species (ROS) levels were quantified in HCT116, HT29 and MCF-7 cell lines by using the fluorescent dye DCFH-DA. Fluorescence induction is proportional to the number of radicals produced by cells which were exposed to metallocenes, and the development of the fluorescence signal was detected every 10 min for two hours. Due to the short incubation time, higher concentrations (2, 20, 100 and 200 µM) than the IC_50_ values determined after 96 h were applied. Pronounced elevation of ROS levels by complexes **1A**, **3** and **9**, which are (*O*,*O*–) chelate bearing tungstenocenes, was observed in all three cell lines (Figure 7, Figures S41 and S42). Complex **10** is the only tungstenocene with a (*S*,*O*–) chelate, which elicits relevant ROS generation. With these four complexes, clear concentration- and time-dependent effects could be observed. Unexpectedly, the behavior of compound **4** differed markedly, because measurements suggest concentration-dependent anti-oxidative properties. However, the intensely red colored complex **4** was found to quench the fluorescence of the product DCF (2′,7′-dichlorofluorescein) in a cell-free assay. DCF (5 µM) and complex **4** (20 and 200 µM) were applied alone as well as in combination (DCF (5 µM) + complex **4** (20 µM) and DCF (5 µM) + complex **4** (200 µM)). In the combination with lower concentration of complex **4** 36.8% and with the higher concentration 77.2% lower fluorescence intensities were detected relative to DCF (5 µM) alone (Figure S43). The other tested complexes caused only minor or negligible alterations of ROS signals in a concentration- and time-dependent manner. These findings indicate that ROS production may play a role in the mode of action for at least some of the compounds. For a better understanding of the biological activity of tungstenocenes containing (*O*,*O*–), (*S*,*O*–) and (*N*,*O*–) chelates, the relation between intracellular ROS levels and initiation of apoptosis and necrosis was examined.

### 3.9. Apoptosis Studies

Translocation of phosphatidylserines (PS) is a classic apoptotic hallmark [57,58]. In viable cells, they are located on the inner leaflet of the plasma membrane, whereas they are translocated to outer leaflet in cells undergoing apoptosis. There, they can be detected with fluorescent-labeled annexin V, which specifically binds to PS. Besides annexin V, the DNA-intercalating agent propidium iodide (PI) is used to stain the nuclei of necrotic and late apoptotic cells. This annexin V-FITC/PI double staining enables a differentiation between viable cells, which are negative for both dyes (AV−/PI−), early apoptotic cells, which are annexin V positive and PI negative (AV+/PI−), late apoptotic cells, which are positive for both annexin V and PI (AV+/PI+), and necrotic cells, which are positive for PI only (AV−/PI+) [59,60].

**Figure 8:**
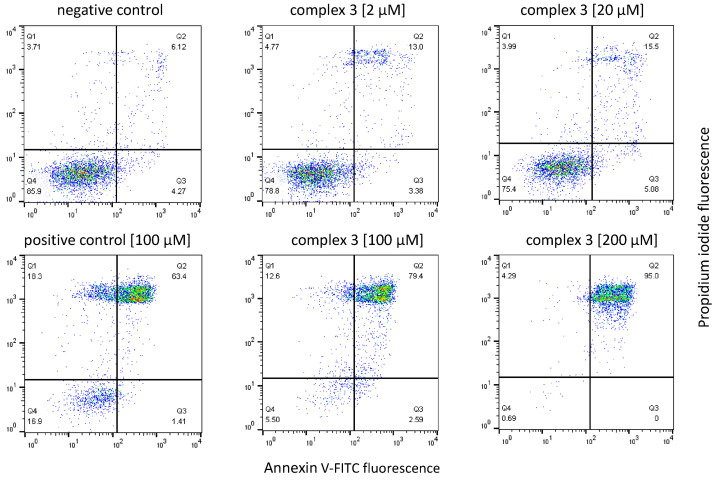
Flow-cytometric analysis (annexin V/PI assay) of apoptosis induction by complex **3** after 24 h in HCT116 cells. Scatter plots represent necrotic (Q1: AV−/PI+), late apoptotic (Q2: AV+/PI+), early apoptotic (Q3: AV+/PI−) and viable (Q4: AV−/PI−) cell fractions (percentages indicated correspondingly) of a representative experiment.

In accordance with ROS and cytotoxicity studies (resazurin assay), 200 µM were applied of almost all tested tungstenocenes for 24 h; only complexes **3** and **4**, with the most promising cytotoxicity, were examined with a wider concentration range. Flavone complex **3** strongly induced apoptosis at the highest tested concentrations with up to 94% in HCT116 (200 µM), 83% in HT29 (200 µM) and 17.5% in MCF-7 (50 µM) cells (Table S26). Furthermore, complex **3** caused a tremendous concentration-dependent increase in early and late apoptotic phases in all three cell lines (Figure S44), exemplarily illustrated for HCT116 cells in Figure 8. The other potent compound was thioflavone complex **4**, which induced apoptosis even at 20 µM in up to 33% (HCT116), 36% (HT29) and 13% (MCF-7) of cells (Table S26 and Figure S45). In MCF-7 cells a distinct concentration dependency was shown for the detection of total apoptotic events induced by compound **4**; however, also the necrotic population increased. This specific phenomenon was obtained only in MCF-7 cells, whereas in the other two cell lines the necrotic fractions were rather minor (Figure S45). Surprisingly, thiomaltol complex **2** (200 µM), demonstrated strong apoptotic effect in HCT116 cells with up to 34% and in MCF-7 cells with up to 30%. Thioethylmaltol complex **10** (200 µM) also induced apoptosis in up to 52%, but only in MCF-7 cells (Table S26).

To sum up the results of annexin V/PI assay, the tungstenocenes bearing a (*S*,*O*–) chelate revealed a higher ability to induce early or late apoptosis than the complexes with (*O*,*O*–) or (*N*,*O*–) chelates.

### 3.10. Cell-Free DNA Interaction Assay

In order to elucidate if tungstenocenes interact with DNA, a cell-free dsDNA plasmid assay was performed. The intact dsDNA plasmid is mostly present in negatively supercoiled (sc) form, and upon drug interaction it may gradually convert to an open circular (oc) form, linear or interhelically cross-linked DNA, which results in altered electrophoretic mobility of the plasmid. This DNA damage may result from various forms of interaction such as cross-linking, intercalation or strand breakage. Platinum complexes such as cisplatin are capable of altering the secondary structure of the DNA mainly by formation of cross-links [61,62]. To investigate a possible platinum-like behaviour of tungstenocenes, 50 µM of the complexes were incubated with pUC19 plasmid up to 6 h at 37 °C. A considerable interaction was observed by complexes **2**–**4**, **8** and **10**, suggesting single-strand breaks rather than cross-linking (Figure S46) in contrast to cisplatin [63]. Complex **8** showed the highest ability to induce single-strand breaks (15 ± 2%) untwisting the sc form to the oc form, even though this complex did not show pronounced cytotoxicity (Figure S47). From a comparison of the five most dsDNA damaging complexes (**2**–**4**, **8** and **10**), it becomes obvious that all of the (*O*,*S*–) chelate bearing tungstenocenes interact with DNA. The only complex with an (*O*,*O*–) chelate showing the ability to induce DNA nicks is complex **3**. The other tested tungstenocenes did not induce any or only negligible changes in the electrophoretic pattern. The minor effects on dsDNA observed and their poor correlation with cytotoxic potency argue against a crucial involvement of DNA interactions in the mode of action of the tested compounds.

## 4. Conclusions

Severe side effects, acquired tumor resistance and a limited number of responsive tumors are the limiting factors for platinum-based anticancer drug therapy in the clinic. Among the diverse metal compounds that might overcome these obstacles by potentially different modes of action and more selective anticancer activity, tungsten complexes have hardly received any attention thus far. Hence, we synthesized and characterized ten tungstenocene derivatives bearing different ligand scaffolds with various coordination motifs. Stability studies over 24 h in aqueous solution (1% DMSO in 10% PBS) via UV/Vis spectroscopy showed that some compounds precipitated over time; however, no hydrolysis or decomposition is indicated. The electrochemistry of all compounds was investigated by cyclic voltammetry. Although a direct comparison to the physiological system is not possible, due to measurements in MeCN, all complexes except for **6** show reversible W^IV^ to W^V^ peaks at such high potentials that decomposition due to redox processes in the physiological range should not be problematic.

Very auspicious in vitro anticancer activity was found in six human cell lines (A549, CH1/PA-1, HCT116, HT29, MCF-7, SW480), especially for complexes **3** and **4**, exhibiting IC_50_ values mostly in the low micromolar range in monolayer cultures. An impact of the bidentate ligand donor system on the cytotoxicity was observed, with (*S,O*–) donors demonstrating improved IC_50_ values when compared to the (*O,O*–) donors. Furthermore, a certain selectivity of compounds **2**, **3**, **4** and **10** for malignant cells over non-malignant fibroblasts was observed. The cytotoxicity of complexes **3** and **4** is hardly affected in 3D cell culture (IC_50_ ratios 3D/2D: 2.3 and 2.5, respectively). Growth curves of HCT116, HT29 and MCF-7 multicellular tumor spheroids under treatment with complexes **3** and **4** (both 10 µM) indicate that both complexes were able to inhibit or completely stop growth and induce changes in the morphology of spheroids within the first 24 h of a 96-h treatment. To support these results, proliferating cells in cryo-sectioned HCT116 spheroids were detected via immunofluorescence imaging of KI67. Notable decreases of KI67^+^ cells were observed upon treatment with complexes **3** and **4** after 72 h incubation. A 2.4-fold reduction in number of KI67^+^ cells by complex **3** (20 µM) and 2-fold reduction by complex **4** (15 µM) was observed relative to the untreated control, (*p* < 0.05, respectively). Both compounds inhibited the proliferation of cells in the periphery of the spheroid with differing distribution patterns of KI67^+^ cells. This prompted us to investigate the W distribution in cryo-sectioned HCT116 spheroids via LA-ICP-TOF-MS. The signal intensity maps of W differed fundamentally, suggesting that the flavone-bearing complex **3** is more capable of entering deeper parts of the spheroids, whereas the thio-analogue **4** showed a higher accumulation in the outer rim of the spheroids.

To dig deeper into the mode of action of the novel tungstenocenes, the potential to form reactive oxygen species and apoptosis induction were studied in monolayer cultures of three cell lines (HCT116, HT29, MCF-7). The measured intracellular ROS levels spanned a wide range from slightly antioxidant up to highly oxidant effects, depending on the cell line as well. In all three cell lines an increase of ROS upon treatment with complexes **1A**, **3**, **9** and **10** (except complex **3** in MCF-7 cells) was detected. Complex **10** is the only tungstenocene with a (*S*,*O*–) chelate which was able to induce ROS development. Both complexes **3** and **4** induced apoptosis (and necrosis) in a concentration-dependent manner in all three cell lines. Furthermore, the capacity of altering DNA secondary structure of the ten tungstenocenes was investigated in a cell-free assay. Complexes **2**–**4**, **8** and **10** interacted with plasmid dsDNA by causing single-strand breaks to a certain extent, but no other forms of DNA damage were indicated. Over all assays, the (*S*,*O*–) chelate bearing complexes tend to be more active than their (*O*,*O*–) and (*N*,*O*–) counterparts, but the current study also suggests a somewhat different mode of action of these complexes, although they only differ by the presence of a W–S or W–O bond.

## Figures and Tables

**Table 1:** Relevant bond distances for compounds **2**–**10**. Due to the disorder in the structure of **1** it has been omitted. Centroid distances calculated by Platon [44].

	Bond Lengths [Å]				
Compound	W–O2	W–O/S/N	W–Cp_centroid_	Ring Slippage	C1=O/S1
**2**	2.067 (2)	2.441 (1)	1.9821 (8)	0.151	1.710 (2)
**3**	2.072 (7)	2.127 (8)	1.965 (6)–1.979 (6)	0.106–0.147	1.309 (12)
**4**	2.0812 (15)	2.4298 (5)	1.9776 (11)–1.9824 (12)	0.130–0.173	1.699 (2)
**5**	2.097 (9)	2.157 (11)	1.966 (3)–1.972 (3)	0.107–0.128	1.35 (2)
**6**	2.0940 (19)	2.097 (2)	1.9690 (14)–1.9745 (14)	0.143–0.161	1.312 (3)
**7**	2.102 (8)	2.114 (13)	1.964 (5)	0.143	1.32 (2)
**8**	2.091 (11)	2.454 (4)	1.974 (10)–1.975 (9)	0.117–0.153	1.709 (15)
**9**	2.074 (5)	2.118 (4)	1.970 (2)	0.156	1.287 (8)
**10**	2.082 (1)	2.454 (1)	1.9822 (11)–1.9832 (12)	0.125–0.145	1.706 (2)

**Table 2:** Cyclic voltammetry data for compounds **1**–**10** and Cp_2_WCl_2_ in MeCN. The redox potentials are reported vs. NHE.

Compound	E_1/2_ (V)
**1**	1.037
**2**	1.047
**3**	1.059
**4**	1.082
**5**	1.330
**6**	0.885
**7**	1.071
**8**	1.080
**9**	1.038
**10**	1.064
Cp_2_WCl_2_	0.648

**Table 3:** In vitro cytotoxicity (IC_50_ values) in monolayer cultures of three human cancer cell lines (A549, SW480 and CH1/PA-1) and IMR-90 fibroblasts; 50% inhibitory concentrations (mean ± SD), obtained by the MTT assay (exposure time: 96 h); n. d., not determined * [21] ** [41].

	IC_50_ Values [µM]			
Compound	A549	SW480	CH1/PA-1	IMR-90
[Cp_2_W(L1)]PF_6_ (**1**)	>200	>200	85 ± 41	n. d.
[Cp_2_W(L1)]Cl (**1A**)	>200	>200	147 ± 18	n. d.
[Cp_2_Mo(L1)]PF_6_ *	>200	>200	>200	n. d.
[Cp_2_W(L2)]PF_6_ (**2**)	36 ± 6	36 ± 7	24 ± 3	107 ± 30
[Cp_2_Mo(L2)]PF_6_ *	106 ± 15	55 ± 9	29 ± 10	n. d.
[Cp_2_W(L3)]PF_6_ (**3**)	19 ± 3	5.4 ± 1.1	2.7 ± 0.4	38.2 ± 0.4
[Cp_2_Mo(L3)]PF_6_ *	24 ± 4	5.6 ± 0.2	1.7 ± 0.4	n. d.
[Cp_2_W(L4)]PF_6_ (**4**)	6.3 ± 0.5	2.4 ± 0.5	1.6 ± 0.5	11 ± 2
[Cp_2_Mo(L4)]PF_6_ *	5.7 ± 1.0	1.6 ± 0.2	0.72 ± 0.13	n. d.
[Cp_2_W(L5)]PF_6_ (**5**)	>200	147 ± 21	>200	n. d.
[Cp_2_Mo(L5)]PF_6_ *	>200	>200	>200	n. d.
**6**	141 ± 36	154 ± 29	>200	n. d.
**7**	>200	>200	152 ± 16	n. d.
**8**	>200	129 ± 14	93 ± 15	n. d.
**9**	>200	101 ± 15	>200	n. d.
**10**	53 ± 8	13 ± 1	21 ± 1	114 ± 33
Cp_2_WCl_2_	>200	38 ± 21	2.4 ± 0.5	n. d.
Cp_2_MoCl_2_	>200 *	>200 *	>200 *	n. d.
Cisplatin	3.8 ± 1.0 **	2.3 ± 0.2 **	0.073 ± 0.001 **	14 ± 1

**Table 4:** Inhibition of cancer cell growth (IC_50_) in 2D and 3D cultures of the human cancer cell lines HCT116, HT29 and MCF-7; 50% inhibitory concentrations (mean ± SD) of Cp_2_WCl_2_ and ten tungstenocenes, obtained by the resazurin assay (exposure time: 96 h).

	IC_50_ Values [µM]					
Compound	HCT116		HT29		MCF-7	
	**2D**	**3D**	**2D**	**3D**	**2D**	**3D**
**1A**	>200	>200	>200	>200	>200	>200
**2**	98 ± 12	158 ± 28	53 ± 9	>200	66 ± 12	>200
**3**	9.2 ± 1.4	21 ± 4	17 ± 4	86 ± 2	6.5 ± 1.8	44 ± 1
**4**	6.0 ± 0.9	15 ± 4	5.5 ± 1.0	31 ± 6	2.7 ± 0.7	19 ± 2
**5**	>200	>200	>200	>200	>200	>200
**6**	>200	>200	>200	>200	172 ± 45	>200
**7**	>200	>200	>200	>200	>200	>200
**8**	>200	178 ± 14	>200	>200	163 ± 32	>200
**9**	>200	>200	>200	>200	147 ± 15	>200
**10**	42 ± 3	69 ± 12	54 ± 2	>200	29 ± 4	>200
Cp_2_WCl_2_	>100	>100	>100	>100	>100	>100

## Data Availability

CCDC 2074974–2074983 contain the supplementary crystallographic data for this paper. These data can be obtained free of charge via www.ccdc.com.ac.uk/data_request/cif, or by emailing data_request@ccdc.cam.ac.uk, or by contacting the Cambridge Crystallographic Data Centre, 12 Union Road, Cambridge CB2 1EZ, UK; Fax: +44-1223-336033.

## Supplementary Materials

The following supporting information can be downloaded at: https://www.mdpi.com/article/10.3390/pharmaceutics15071875/s1, Synthesis of ligands, materials, NMR-spectroscopy (Figures S1–S11), mass spectrometry data (Table S1), experimental parameters for X-ray analysis (Table S2), sample and crystal data of complexes, data collection and structure refinement of compound (Tables S3–S22), cyclic voltammetry data (Figures S12–S17), UV/Vis spectroscopy data (Figures S18–S27), wavelength of the peak maxima and molar extinction coefficients (Table S23), concentration-effect curves of MTT assay (Figures S28 and S29), resazurin assay (Table S24, Figures S30–S32), time-dependent growth of spheroids and growth curves (Figures S33–S36), statistical data of immunofluorescence staining (Table S25, Figure S37), laser ablation (Figures S38–S40), reactive oxygen species detection (Figures S41 and S42), fluorescence-quenching assay (Figure S43), apoptosis induction (Figures S44 and S45, Table S26), dsDNA plasmid assay (Figures S46 and S47). Reference [42] is cited in the Supporting Information.

## References

[1] Gasser G., Ott I., Metzler-Nolte N. (2011). Organometallic anticancer compounds. J. Med. Chem..

[2] Jaouen G., Top S., Vessières A. (2005). Organometallics Targeted to Specific Biological Sites: The Development of New Therapies. Bioorganometallics: Biomolecules, Labeling, Medicine.

[3] Schirrmacher V. (2017). Quo Vadis Cancer Therapy?.

[4] Keith L.S., Moffett D.B., Rosemond Z.A., Wohlers D.W. (2007). ATSDR evaluation of health effects of tungsten and relevance to public health. Toxicol. Ind. Health.

[5] Vyskočil A., Viau C. (1999). Assessment of molybdenum toxicity in humans. J. Appl. Toxicol..

[6] Köpf H., Köpf-Maier P. (1979). Titanocene Dichloride—The First Metallocene with Cancerostatic Activity. Angew. Chem. Int. Ed. Engl..

[7] Köpf-Maier P., Leitner M., Voigtländer R., Köpf H. (1979). Molybdocene Dichloride as an Antitumor Agent. Z. fur Nat. CA J. Biosci..

[8] Köpf-Maier P., Leitner M., Köpf H. (1980). Tumor Inhibition by Metallocenes: Antitumor Activity of Niobocene and Tungstocene Dichlorides. J. Inorg. Nucl. Chem..

[9] Köpf-Maier P., Köpf H. (1979). Vanadocen-dichlorid—Ein weiteres Antitumor-Agens aus der Metallocenreihe/Vanadocene Dichloride—Another Antitumor Agent from the Metallocene Series. Z. Nat. B.

[10] Ghosh P., D’Cruz O.J., Narla R.K., Uckun F.M. (2000). Apoptosis-Inducing Vanadocene Compounds against Human Testicular Cancer. Clin. Cancer Res..

[11] Harding M.M., Mokdsi G. (2000). Antitumour Metallocenes: Structure-Activity Studies and Interactions with Biomolecules. Curr. Med. Chem..

[12] Köpf-Maier P. (1994). Complexes of Metals Other than Platinum as Antitumour Agents. Eur. J. Clin. Pharmacol..

[13] Strohfeldt K., Tacke M. (2008). Bioorganometallic fulvene-derived titanocene anti-cancer drugs. Chem. Soc. Rev..

[14] Waern J.B., Harding M.M. (2004). Bioorganometallic chemistry of molybdocene dichloride. J. Organomet. Chem..

[15] Gao L.M., Vera J.L., Matta J., Meléndez E. (2010). Synthesis and cytotoxicity studies of steroid-functionalized titanocenes as potential anticancer drugs: Sex steroids as potential vectors for titanocenes. J. Biol. Inorg. Chem..

[16] Dominguez-Garcia M., Ortega-Zuniga C., Meléndez E. (2013). New tungstenocenes containing 3-hydroxy-4-pyrone ligands: Antiproliferative activity on HT-29 and MCF-7 cell lines and binding to human serum albumin studied by fluorescence spectroscopy and molecular modeling methods. J. Biol. Inorg. Chem..

[17] Gomez-Ruiz S., Maksimovic-Ivanic D., Mijatovic S., Kaluderovic G.N. (2012). On the discovery, biological effects, and use of Cisplatin and metallocenes in anticancer chemotherapy. Bioinorg. Chem. Appl..

[18] Meléndez E. (2012). Metallocenes as Target Specific Drugs for Cancer Treatment. Inorg. Chim. Acta.

[19] Kuo L.Y., Kanatzidis M.G., Sabat M., Tipton A.L., Marks T.J. (1991). Metallocene Antitumor Agents. Solution and Solid-State Molybdenocene Coordination Chemistry of DNA Constituents. J. Am. Chem. Soc..

[20] Balzarek C., Weakley T.J.R., Kuo L.Y., Tyler D.R. (2000). Investigation of the Monomer−Dimer Equilibria of Molybdocenes in Water. Organometallics.

[21] Kandioller W., Reikersdorfer M., Theiner S., Roller A., Hejl M., Jakupec M.A., Malarek M.S., Keppler B.K. (2018). The Impact of Leaving Group Variation on the Anticancer Activity of Molybdenocenes. Organometallics.

[22] Feliciano I., Matta J., Meléndez E. (2009). Water-soluble molybdenocene complexes with both proliferative and antiproliferative effects on cancer cell lines and their binding interactions with human serum albumin. J. Biol. Inorg. Chem..

[23] Meléndez E. (2012). Bioorganometallic Chemistry of Molybdenocene Dichloride and Its Derivatives. J. Organomet. Chem..

[24] Fuchs V., Cseh K., Hejl M., Vician P., Neuditschko B., Meier-Menches S.M., Janker L., Bileck A., Gajic N., Kronberger J. (2022). Highly Cytotoxic Molybdenocenes with Strong Metabolic Effects Inhibit Tumour Growth in Mice. Chem. Eur. J..

[25] Kandioller W., Kurzwernhart A., Hanif M., Meier S.M., Henke H., Keppler B.K., Hartinger C.G. (2011). Pyrone derivatives and metals: From natural products to metal-based drugs. J. Organomet. Chem..

[26] Kandioller W., Hartinger C.G., Nazarov A.A., Bartel C., Skocic M., Jakupec M.A., Arion V.B., Keppler B.K. (2009). Maltol-derived ruthenium-cymene complexes with tumor inhibiting properties: The impact of ligand-metal bond stability on anticancer activity in vitro. Chemistry.

[27] Kandioller W., Hartinger C.G., Nazarov A.A., Kuznetsov M.L., John R.O., Bartel C., Jakupec M.A., Arion V.B., Keppler B.K. (2009). From Pyrone to Thiopyrone Ligands−Rendering Maltol-Derived Ruthenium(II)−Arene Complexes That Are Anticancer Active in Vitro. Organometallics.

[28] Reddy V.D., Dayal D., Szalda D.J., Cosenza S.C., Ramana Reddy M.V. (2012). Syntheses, structures, and anticancer activity of novel organometallic ruthenium–maltol complexes. J. Organomet. Chem..

[29] Bhattacharya S., Seth N., Gupta V.D., Nöth H., Polborn K., Thomann M., Schwenk H. (1994). Synthesis and Structure of Organotin(IV) Complexes of Maltol. Chem. Ber..

[30] Lamboy J.L., Pasquale A., Rheingold A.L., Meléndez E. (2007). Synthesis, Solution and Solid State Structure of Titanium-Maltol Complex. Inorg. Chim. Acta.

[31] Berasaluce I., Cseh K., Roller A., Hejl M., Heffeter P., Berger W., Jakupec M.A., Kandioller W., Malarek M.S., Keppler B.K. (2020). The First Anticancer Tris(pyrazolyl)borate Molybdenum(IV) Complexes: Tested In Vitro and In Vivo-A Comparison of O,O-, S,O-, and N,N-Chelate Effects. Chem. Eur. J..

[32] Middleton E., Kandaswami C., Theoharides T.C. (2000). The Effects of Plant Flavonoids on Mammalian Cells: Implications for Inflammation, Heart Disease, and Cancer. Pharmacol. Rev..

[33] Grant R.S., Coggan S.E., Smythe G.A. (2009). The Physiological Action of Picolinic Acid in the Human Brain. Int. J. Tryptophan Res. IJTR.

[34] Yasumoto E., Nakano K., Nakayachi T., Morshed S.R.M., Hashimoto K., Kikuchi H., Nishikawa H., Kawase M., Sakagami H. (2004). Cytotoxic Activity of Deferiprone, Maltol and Related Hydroxyketones against Human Tumor Cell Lines. Anticancer Res..

[35] Williams D.B., Lawton M. (2010). Drying of organic solvents: Quantitative evaluation of the efficiency of several desiccants. J. Org. Chem..

[36] Chaves S., Gil M., Canario S., Jelic R., Romao M.J., Trincao J., Herdtweck E., Sousa J., Diniz C., Fresco P. (2008). Biologically relevant O,S-donor compounds. Synthesis, molybdenum complexation and xanthine oxidase inhibition. Dalton Trans..

[37] Kurzwernhart A., Kandioller W., Bachler S., Bartel C., Martic S., Buczkowska M., Mühlgassner G., Jakupec M.A., Kraatz H.-B., Bednarski P.J. (2012). Structure−Activity Relationships of Targeted Ru^II^(*η^6^-p*-Cymene) Anticancer Complexes with Flavonol-Derived Ligands. J. Med. Chem..

[38] Enyedy E.A., Sija E., Jakusch T., Hartinger C.G., Kandioller W., Keppler B.K., Kiss T. (2013). Solution equilibria of anticancer ruthenium(II)-(eta(6)-p-cymene)-hydroxy(thio)pyr(id)one complexes: Impact of sulfur vs. oxygen donor systems on the speciation and bioactivity. J. Inorg. Biochem..

[39] Agrawal A., Johnson S.L., Jacobsen J.A., Miller M.T., Chen L.H., Pellecchia M., Cohen S.M. (2010). Chelator fragment libraries for targeting metalloproteinases. ChemMedChem.

[40] Pavlishchuk V.V., Addison A.W. (2000). Conversion Constants for Redox Potentials Measured versus Different Reference Electrodes in Acetonitrile Solutions at 25 °C. Inorg. Chim. Acta.

[41] Cseh K., Geisler H., Stanojkovska K., Westermayr J., Brunmayr P., Wenisch D., Gajic N., Hejl M., Schaier M., Koellensperger G. (2022). Arene Variation of Highly Cytotoxic Tridentate Naphthoquinone-Based Ruthenium(II) Complexes and In-Depth In Vitro Studies. Pharmaceutics.

[42] Scaffidi-Domianello Y.Y., Legin A., Jakupec M.A., Arion V.B., Kukushkin V.Y., Galanski M.S., Keppler B.K. (2011). Synthesis, characterization, and cytotoxic activity of novel potentially pH-sensitive nonclassical platinum(II) complexes featuring 1,3-dihydroxyacetone oxime ligands. Inorg. Chem..

[43] Socrates G. (2002). Infrared and Raman Characteristic Group Frequencies: Tables and Charts.

[44] Spek A.L. (2009). Structure Validation in Chemical Crystallography. Acta Crystallogr. D.

[45] Berger W., Elbling L., Micksche M. (2000). Expression of the major vault protein LRP in human non-small-cell lung cancer cells: Activation by short-term exposure to antineoplastic drugs. Int. J. Cancer.

[46] Kubanik M., Tu J.K.Y., Söhnel T., Hejl M., Jakupec M.A., Kandioller W., Keppler B.K., Hartinger C.G. (2015). Expanding on the Structural Diversity of Flavone- Derived RutheniumII(ƞ6-arene) Anticancer Agents. Metallodrugs.

[47] Khater M., Ravishankar D., Greco F., Osborn H.M. (2019). Metal complexes of flavonoids: Their synthesis, characterization and enhanced antioxidant and anticancer activities. Future Med. Chem.

[48] Duval K., Grover H., Han L., Mou Y., Pegoraro A.F., Fredberg J., Chen Z. (2017). Modeling Physiological Events in 2D vs. 3D Cell Culture. Physiology.

[49] Fontoura J.C., Viezzer C., Dos Santos F.G., Ligabue R.A., Weinlich R., Puga R.D., Antonow D., Severino P., Bonorino C. (2020). Comparison of 2D and 3D cell culture models for cell growth, gene expression and drug resistance. Mater. Sci. Eng. C.

[50] Nirmalanandhan V.S., Duren A., Hendricks P., Vielhauer G., Sittampalam G.S. (2010). Activity of anticancer agents in a three-dimensional cell culture model. Drug Dev. Technol..

[51] Ravi M., Paramesh V., Kaviya S.R., Anuradha E., Paul Solomon F.D. (2014). 3D Cell Culture Systems Advantages and Applications. J. Cell. Physiol..

[52] Wang H., Brown P.C., Chow E.C.Y., Ewart L.S., Ferguson S.S., Fitzpatrick S., Freedman B.S., Guo G.L., Hedrich W., Heyward S. (2021). 3D cell culture models: Drug pharmacokinetics, safety assessment, and regulatory consideration. Clin. Transl. Sci..

[53] Bonnier F., Keating M.E., Wróbel T.P., Majzner K., Baranska M., Garcia-Munoz A., Blanco A., Byrne H.J. (2015). Cell viability assessment using the Alamar blue assay: A comparison of 2D and 3D cell culture models. Toxicol. In Vitro.

[54] Uxa S., Castillo-Binder P., Kohler R., Stangner K., Müller G.A., Engeland K. (2021). Ki-67 gene expression. Cell Death Differ..

[55] Sun X., Kaufman P.D. (2018). Ki-67: More than a proliferation marker. Chromosoma.

[56] Li L.T., Jiang G., Chen Q., Zheng J.N. (2015). Ki67 is a promising molecular target in the diagnosis of cancer (Review). Mol. Med. Rep.

[57] Sharma B., Kanwar S.S. (2018). Phosphatidylserine: A cancer cell targeting biomarker. Semin. Cancer Biol..

[58] Nagata S. (2018). Apoptosis and Clearance of Apoptotic Cells. Annu. Rev. Immunol..

[59] Kupcho K., Shultz J., Hurst R., Hartnett J., Zhou W., Machleidt T., Grailer J., Worzella T., Riss T., Lazar D. (2019). A real-time, bioluminescent annexin V assay for the assessment of apoptosis. Apoptosis.

[60] Eray M., Mättö M., Kaartinen M., Andersson L.C., Pelkonen J. (2001). Flow Cytometric Analysis of Apoptotic Subpopulations with a Combination of Annexin V-FITC, Propidium Iodide, and SYTO 17. Cytometry.

[61] Göschl S., Schreiber-Brynzak E., Pichler V., Cseh K., Heffeter P., Jungwirth U., Jakupec M.A., Berger W., Keppler B.K. (2017). Comparative studies of oxaliplatin-based platinum(IV) complexes in different in vitro and in vivo tumor models. Metallomics.

[62] Valiahdi S.M., Heffeter P., Jakupec M.A., Marculescu R., Berger W., Rappersberger K., Keppler B.K. (2009). The gallium complex KP46 exerts strong activity against primary explanted melanoma cells and induces apoptosis in melanoma cell lines. Melanoma Res..

[63] Geisler H., Westermayr J., Cseh K., Wenisch D., Fuchs V., Harringer S., Plutzar S., Gajic N., Hejl M., Jakupec M.A. (2021). Tridentate 3-Substituted Naphthoquinone Ruthenium Arene Complexes: Synthesis, Characterization, Aqueous Behavior, and Theoretical and Biological Studies. Inorg. Chem..

