# Supplementary material for: Anticancer Tungstenocenes with a Diverse Set of (O,O–), (O,S–) and (O,N–) Chelates—A Detailed Biological Study Using an Improved Evaluation via 3D Spheroid Models
Source: Pharmaceutics. 2023 Jul 3;15(7):1875. doi: 10.3390/pharmaceutics15071875 (PMC10384408; doi:10.3390/pharmaceutics15071875)
Supplement: Supplementary file 1 [file pharmaceutics-15-01875-s001.zip › pharmaceutics-2344313-supplementary.pdf]

SUPPORTING INFORMATION

Anticancer tungstenocenes with a diverse set of (*O,O*–) (*O,S*–) and (*O,N*–) chelates – a detailed biological study using an improved evaluation *via* 3D spheroid models

Klaudia Cseh [1], Iker Berasaluce [1], Valentin Fuchs [1], Alexandra Banc [1], Andreas Schweikert [1,2], Alexander Prado-Roller [1], Michaela Hejl [1], Debora Wernitznig [1], Gunda Koellensperger [2], Michael A. Jakupec [1,3], Wolfgang Kandioller [1,3], Michael S. Malarek [1*] and Bernhard K. Keppler[1,3]

1 Institute of Inorganic Chemistry, Faculty of Chemistry, University of Vienna, Waehringer Straße 42, A 1090 Vienna, Austria

2 Institute of Analytical Chemistry, Faculty of Chemistry, University of Vienna, Waehringer Straße 38, A 1090 Vienna, Austria

3 Research Cluster "Translational Cancer Therapy Research", University of Vienna, Waehringer Straße 42, A 1090 Vienna, Austria

* Correspondence: michael.malarek@univie.ac.at ; Tel.: +43-1-4277-52670

TABLE OF CONTENTS

Synthesis of Ligands 3

NMR-spectroscopy 6

Mass Spectrometry 12

X-ray diffraction analysis 13

Cyclic Voltammetry 24

UV/Vis spectroscopy 28

MTT assay 34

Resazurin assay 36

Growth curve 39

Immunofluorescence staining 41

Laser ablation 42

Reactive oxygen species (ROS) detection 44

Apoptosis studies 47

Electrophoretic dsDNA plasmid assay 51

References 52

3-Hydroxy-2-methyl-4H-pyran-4-thione (L2)

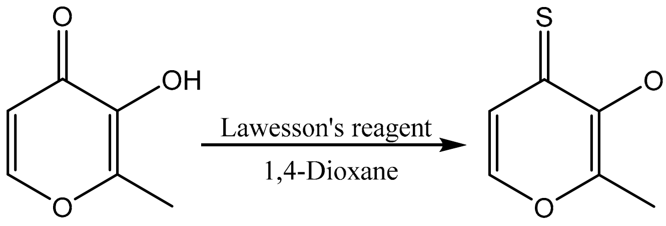

$C_6H_6O_3$
M= 126,11 g/mol

$C_6H_6O_2S$
M= 142,18 g/mol

Maltol (1.00 g, 8.2 mmol) and Lawesson's reagent (1.70 g, 4.2 mmol) were dissolved in 1,4-dioxane (20 mL) and refluxed for 4 hours. The solvent was removed and after column chromatography (n-hexane/ethyl acetate = 10:1) the yellow crystalline product was obtained and dried *in vacuo*.

Yield: 0.368 mg (32%), yellow crystals

NMR-Spectroscopy: ^{1}H-NMR (d_6-DMSO): δ = 2.40 (s, 3H, CH_3), 7.35 (d, 1H, ^3J(H, H)= 5 Hz, H5), 8.0 (d, 1H, ^3J(H, H) = 5 Hz, H6), 8.28 (s, 1H, OH).

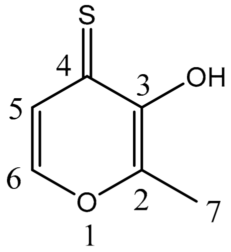

5-Hydroxy-2-methyl-4H-pyran-4-thione (L8)

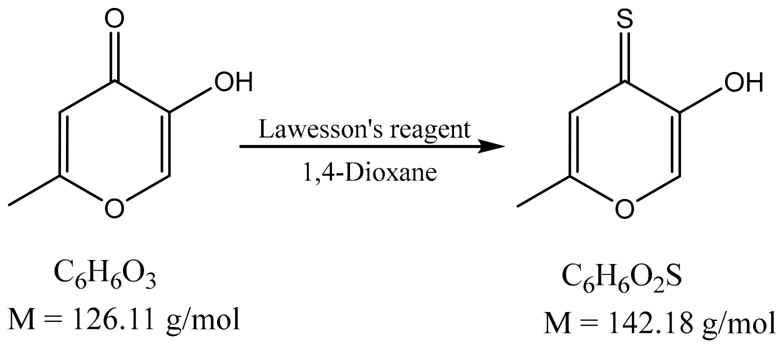

Allomaltol (1.00 g, 8.2 mmol) and Lawesson's reagent (1.70 g, 4.2 mmol) were dissolved in 1,4-dioxane (20 mL) and refluxed for 4 hours. The solvent was removed and after column chromatography (n-hexane/ethyl acetate = 10:1) the yellow crystalline product was obtained and dried *in vacuo*.

Yield: 0.210 mg (17%), yellow crystals

NMR-Spectroscopy: ^{1}H-NMR (d_6-DMSO): δ = 2.33 (s, 3H, CH_3), 7.35 (s, 1H, H3), 8.27 (s, 1H, H6), 8.42 (s, 1H, OH).

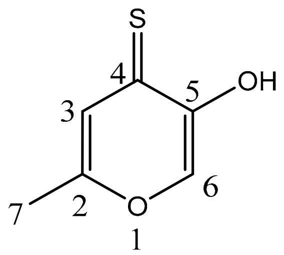

2-Ethyl-3-hydroxy-4H-pyran-4-thione (L10)

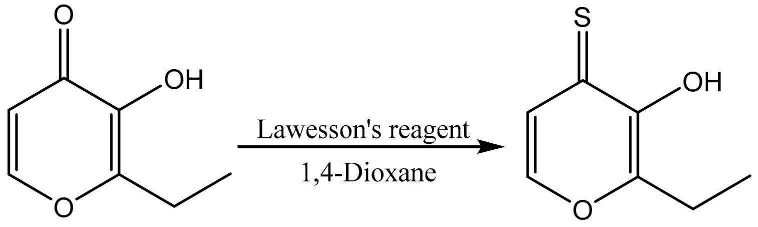

$C_7H_8O_3$ M = 140.14 g/mol

$C_7H_8O_2S$ M = 156.2 g/mol

Ethylmaltol (2.00 g, 14.3 mmol) and Lawesson’s reagent (1.92 g, 4.8 mmol) were dissolved in 1,4-dioxane (40 mL) and refluxed for 4 hours. The solvent was removed and after column chromatography (n-hexane/ethyl acetate = 10:1) the yellow product was obtained and dried *in vacuo*.

Yield: 1.304g (59%), yellow-orange oil

NMR-Spectroscopy: ^{1}H-NMR (d$_6$-DMSO): δ = 1.22 (t, 3H, ^3J(H, H)= 7,6 Hz, CH_3), 2.78 (q, 2H, ^3J(H, H)= 7,6 Hz, CH_2), 7.36 (d, 1H, ^3J(H, H)= 5 Hz, H5), 8.13 (d, 1H, ^3J(H, H)= 4,9 Hz, H6), 8.29 (s, 1H, OH).

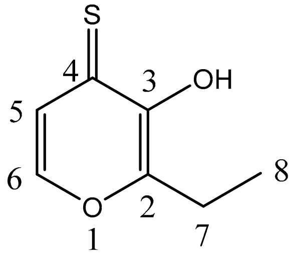

NMR-spectroscopy

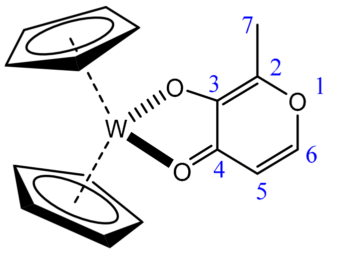

1

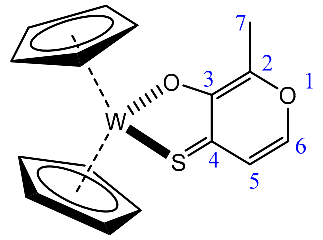

2

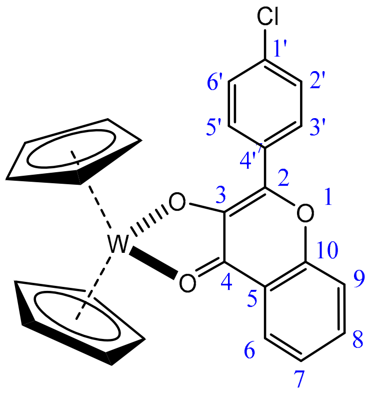

3

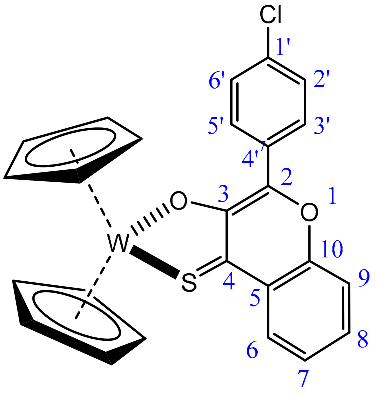

4

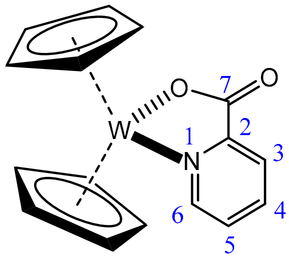

5

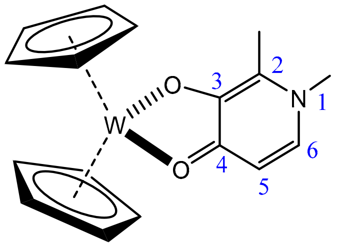

6

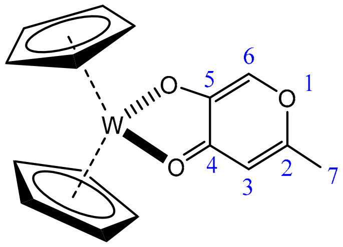

7

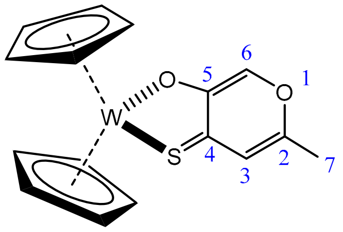

8

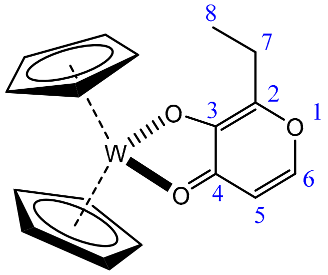

9

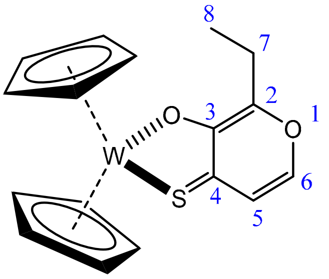

10

Figure S1: Complex atom numbering for NMR peak assignments of compounds **1–10**.

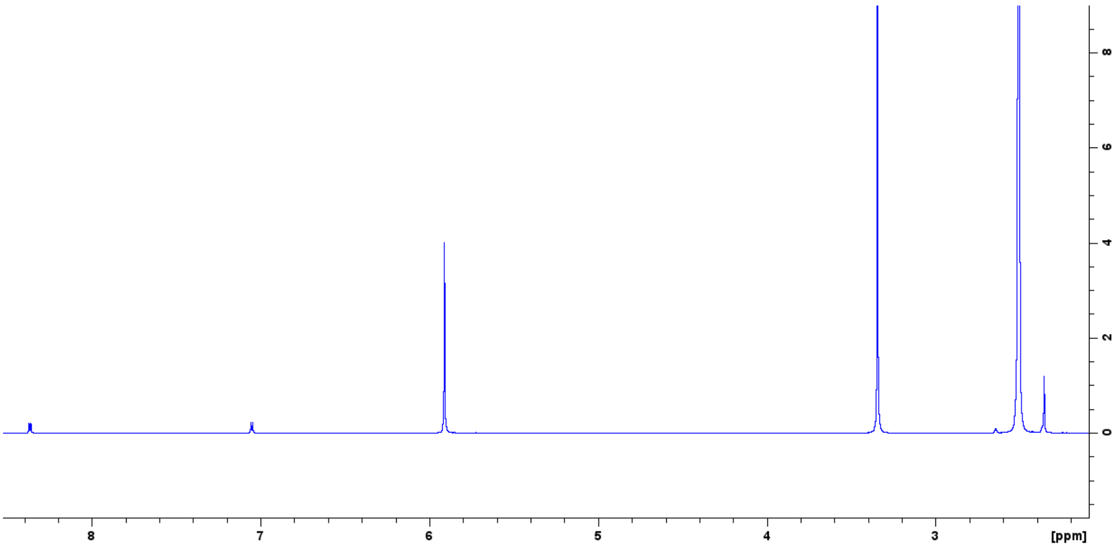

Figure S2: ^{1}H NMR of compound **1** measured in DMSO-d_6.

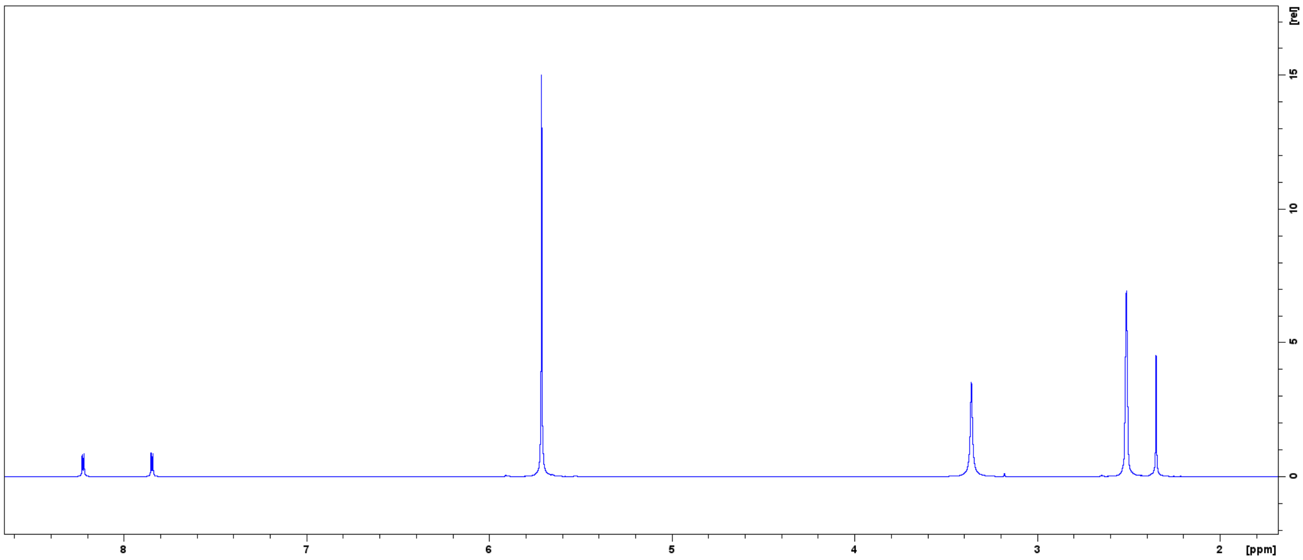

Figure S3: ^{1}H NMR of compound **2** measured in DMSO-d6.

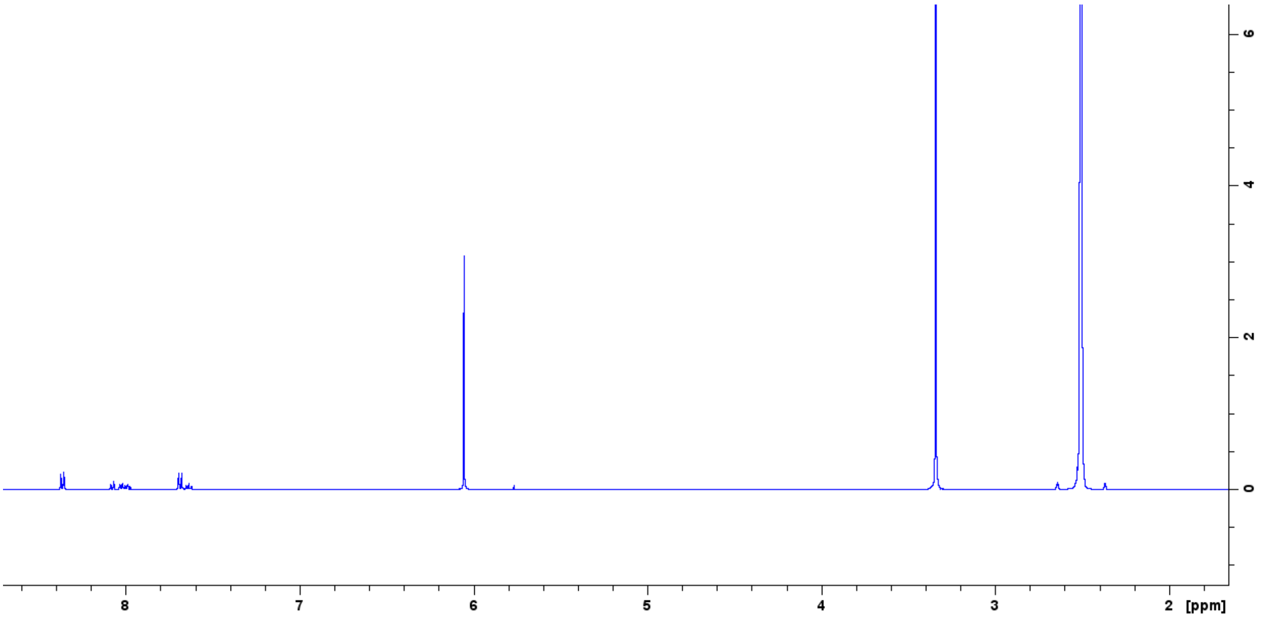

Figure S4: ^{1}H NMR of compound **3** measured in DMSO-d6.

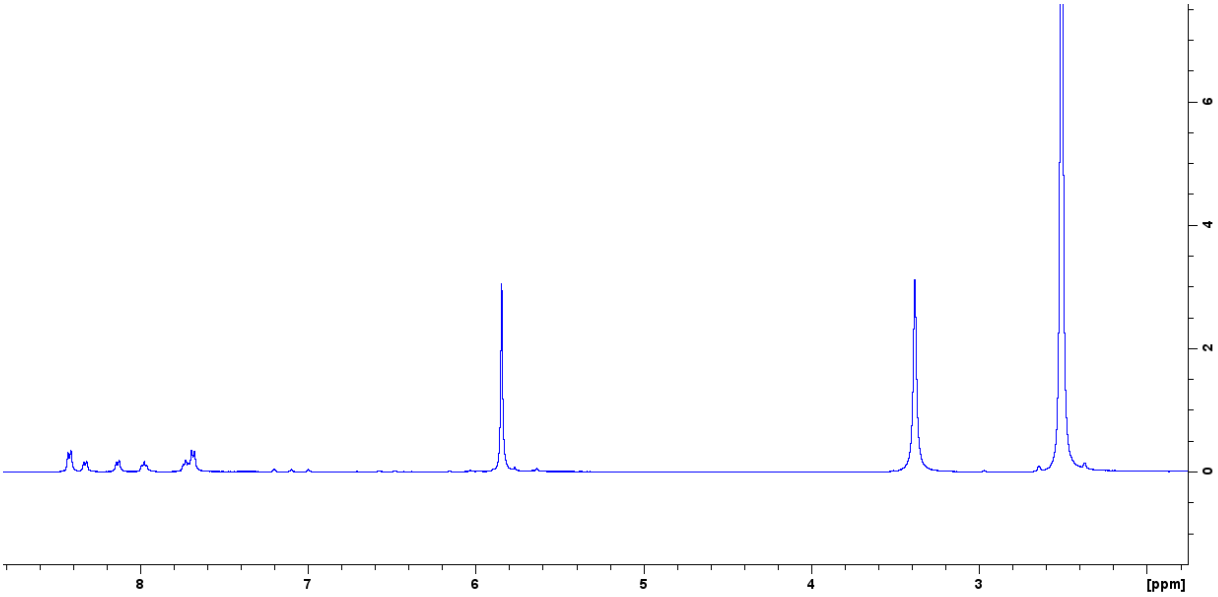

Figure S5: ^{1}H NMR of compound **4** measured in DMSO-d_6.

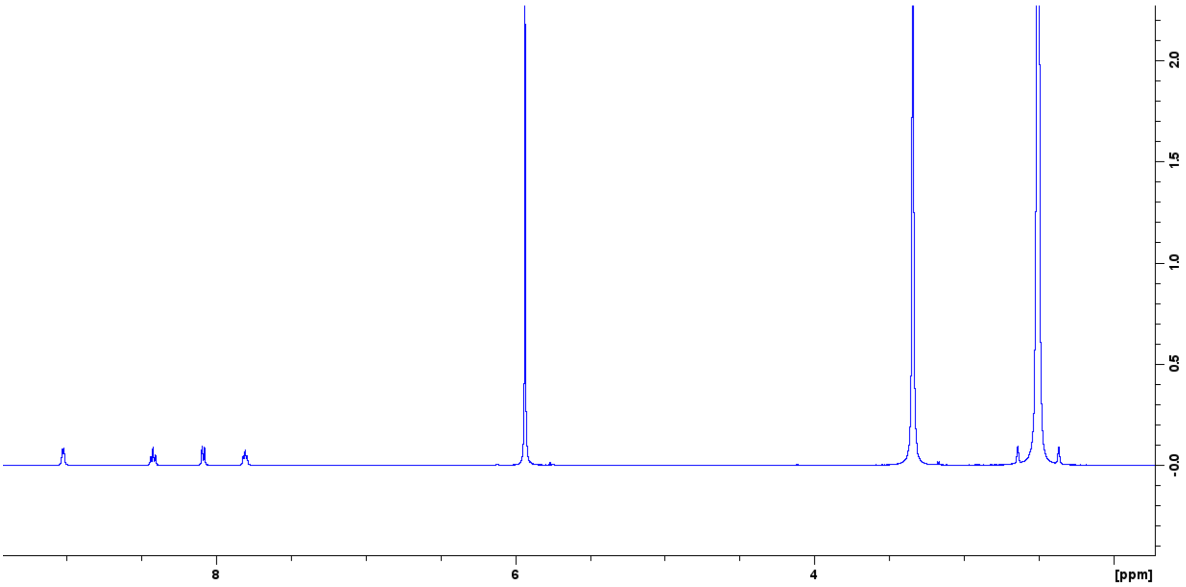

Figure S6: ^{1}H NMR of compound **5** measured in DMSO-d_6.

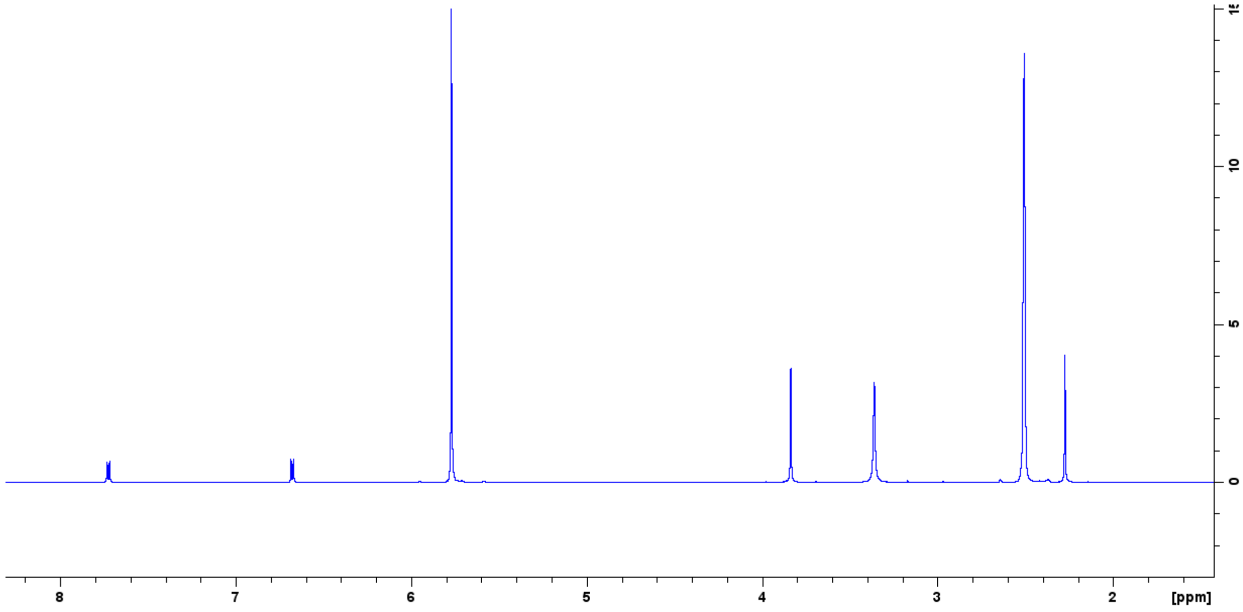

Figure S7: ^{1}H NMR of compound **6** measured in DMSO-d$_6$.

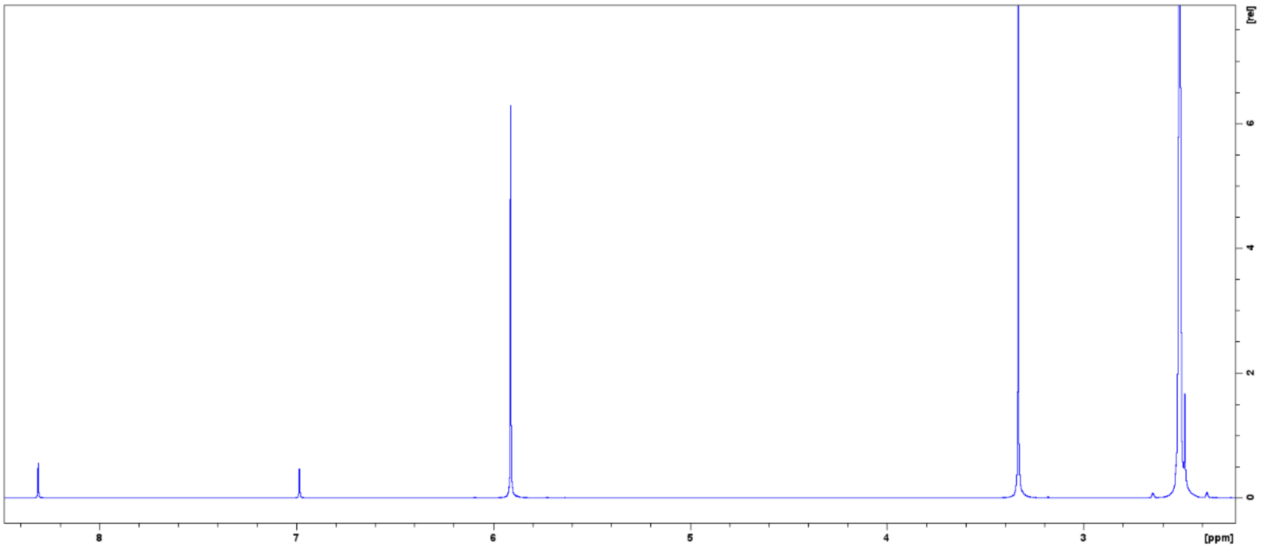

Figure S8: ^{1}H NMR of compound **7** measured in DMSO-d$_6$.

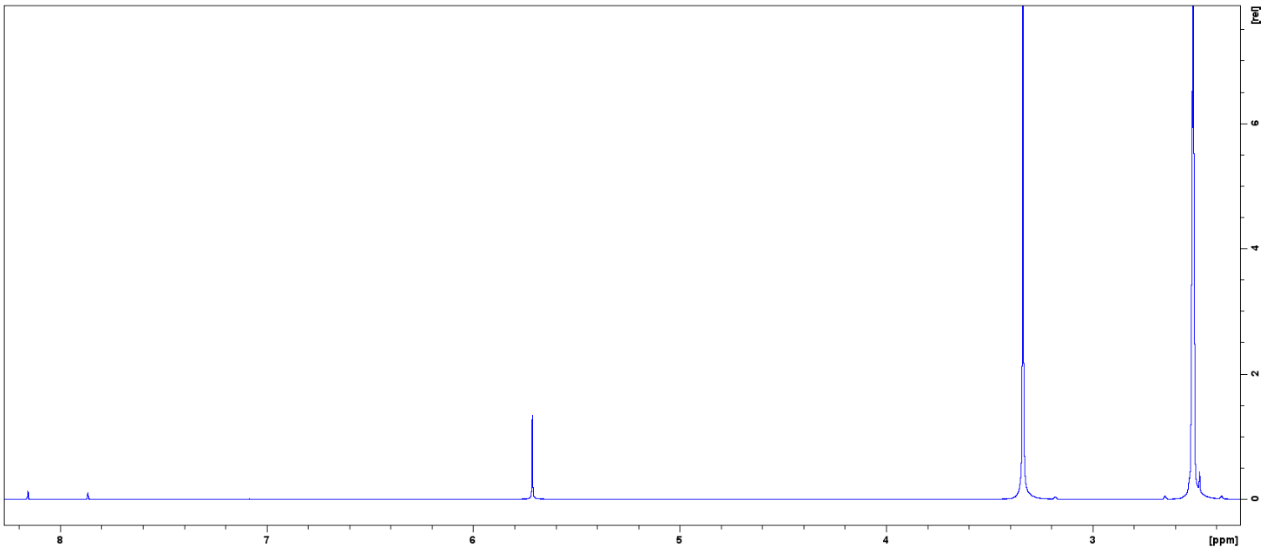

Figure S9: ^{1}H NMR of compound **8** measured in DMSO-d_6.

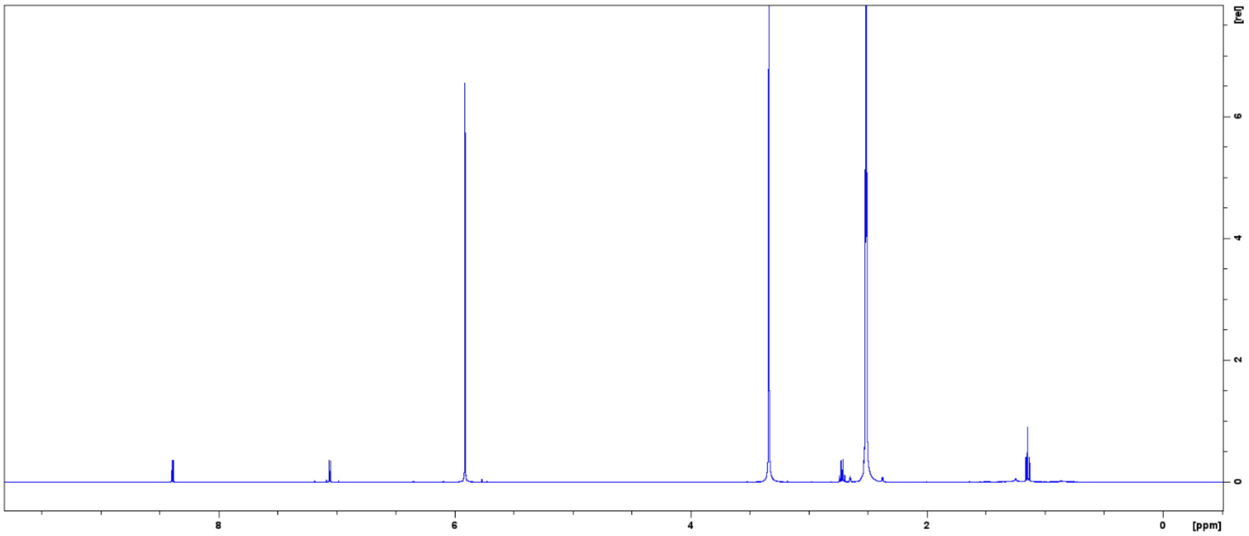

Figure S10: ^{1}H NMR of compound **9** measured in DMSO-d_6.

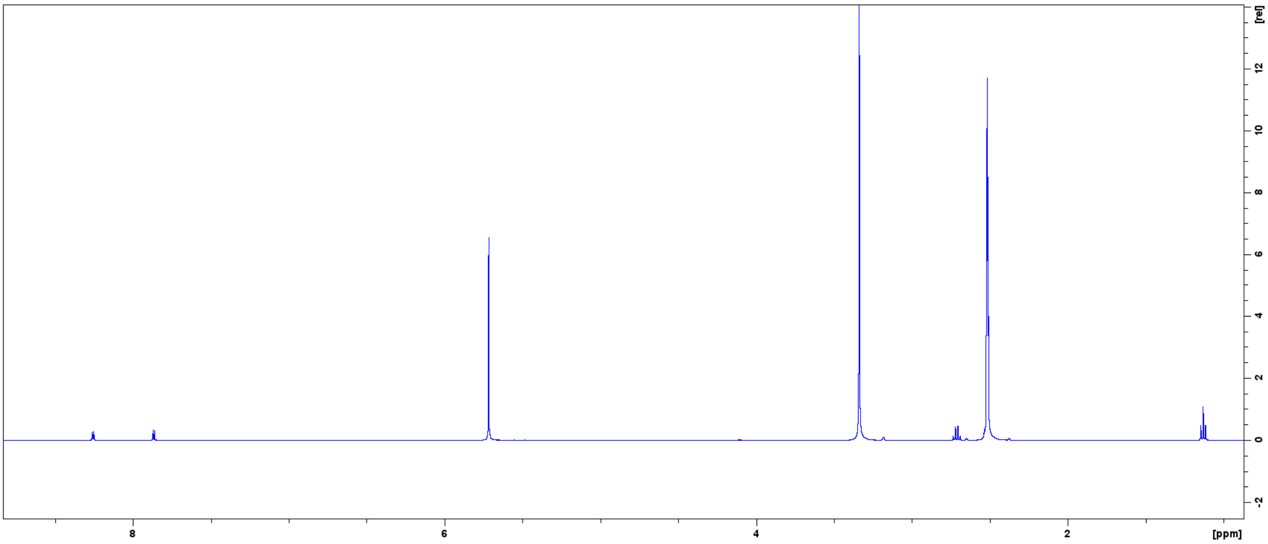

Figure S11: ^{1}H NMR of compound **10** measured in DMSO-d_6.

Mass Spectrometry

Table S1: ESI-MS experimental and theoretical m/z values of compounds 1–10.

Detected ion	m/z	$[Cp_2W(L)]^+_{calc}$
$[Cp_2W($**L1**$)]^+$	439.05	439.05
$[Cp_2W($**L2**$)]^+$	455.17	455.03
$[Cp_2W($**L3**$)]^+$	585.05	585.05
$[Cp_2W($**L4**$)]^+$	601.02	601.10
$[Cp_2W($**L5**$)]^+$	436.05	436.05
$[Cp_2W($**L6**$)]^+$	452.08	452.07
$[Cp_2W($**L7**$)]^+$	439.02	439.05
$[Cp_2W($**L8**$)]^+$	454.98	455.03
$[Cp_2W($**L9**$)]^+$	452.97	453.07
$[Cp_2W($**L10**$)]^+$	468.93	469.05

X-ray diffraction analysis

Table S2: Experimental parameters.

Sample	Machine	Source	Temp.	Detector Distance	Time/ Frame	#Frames	Frame width	CCDC
	Bruker		[K]	[mm]	[s]		[°]	
**1 KP2576**	D8	Mo	100.0	40	6	180	1.000	2074976
**2 Alba112**	D8	Mo	100.0	34	10	473	0.500	2074974
**3 Ikbe318a**	X8	Mo	100.0	35	60	1100	0.500	2074975
**4 Ikbe313a**	X8	Mo	100.0	35	60	1100	0.500	2074977
**5 Ikbe335a**	D8	Cu	100.0	34	10	5316	0.500	2074979
**6 Ikbe334a**	X8	Mo	100.0	35	10	5615	0.500	2074980
**7 Alba119**	D8	Mo	100.0	35	6	758	0.500	2074978
**8 Alba115**	D8	Mo	100.0	35	50	2168	0.500	2074981
**9 Alba139**	D8	Mo	100.0	35	60	239	0.500	2074982
**10 Alba147**	D8	Mo	100.0	35	7	5150	0.500	2074983

Bis(η[5]-cyclopentadienyl)[2-methyl-3-(oxo-κO)-4-(1H)-pyran-4-ato-κO]tungsten(IV) chloride, **1A**

Table S3: Sample and crystal data of compound **1A**.

**Chemical formula**	$C_{16}H_{15}ClO_3W$	**Crystal system**	orthorhombic	
**Formula weight [g/mol]**	474.58	**Space group**	*Cmca*	
**Temperature [K]**	100	**Z**	8	
**Measurement method**	\f and \w scans	**Volume [Å^3]**	3638.3(7)	
**Radiation (Wavelength [Å])**	MoKα (λ = 0.71073)	**Unit cell dimensions [Å] and [°]**	12.8287(10)	90
**Crystal size / [mm^3]**	0.2 × 0.1 × 0.09		24.661(3)	90
**Crystal habit**	clear red needle		11.5002(12)	90
**Density (calculated) / [g/cm^3]**	1.733	**Absorption coefficient / [mm^{-1}]**	6.502	
**Abs. correction Tmin**	0.6395	**Abs. correction Tmax**	0.7452	
**Abs. correction type**	multiscan	**F(000) [e$^-$]**	1808	

Table S4: Data collection and structure refinement of compound **1A**.

**Index ranges**	-15 ≤ h ≤ 14, -28 ≤ k ≤ 29, -13 ≤ l ≤ 13	**Theta range for data collection [°]**	4.844 to 50.724	
**Reflections number**	11264	**Data / restraints / parameters**	1750/24/127	
**Refinement method**	Least squares	**Final R indices**	all data	R1 = 0.0331, wR2 = 0.0497
**Function minimized**	Σ w(F_o^2 - F_c^2)^2		I>2σ(I)	R1 = 0.0245, wR2 = 0.0468
**Goodness-of-fit on F^2**	1.062	**Weighting scheme**	w=1/[σ2(Fo^2)+(0.0116P)^2+4.3718P]	
**Largest diff. peak and hole [e Å^{-3}]**	0.57/-0.66		where P=(F_o^2+2F_c^2)/3	

Bis(η5-cyclopentadienyl)[2-methyl-3-(oxo-κO)-4-(1H)-pyran-4-thionato-κS]tungsten(IV) hexafluorophosphate, **2.**

Table S5: Sample and crystal data of compound **2**.

**Chemical formula**	$C_{16}H_{15}O_2SWPF_6$	**Crystal system**	orthorhombic	
**Formula weight [g/mol]**	600.16	**Space group**	Pnma	
**Temperature [K]**	100	**Z**	4	
**Measurement method**	\Φ and \ω scans	**Volume [Å^3]**	1745.34(7)	
**Radiation (Wavelength [Å])**	MoKα (λ = 0.71073)	**Uni cell dimensions [Å] and [°]**	16.7909(4)	90
**Crystal size / [mm^3]**	0.1 × 0.07 × 0.03		8.3122(2)	90
**Crystal habit**	clear red block		12.5052(3)	90
**Density calculated / [g/cm^3]**	2.284	**Absorption coefficient / [mm^{-1}]**	6.901	
**Abs. correction type**	multiscan	**F(000) [e$^-$]**	1144.0	

Table S6: Data collection and structure refinement of compound **2**.

**Index ranges**	-23 ≤ h ≤ 23, -10 ≤ k ≤ 11, -17 ≤ l ≤ 17	**Theta range for data collection [°]**	4.852 to 60.138	
**Reflections number**	14654	**Data / restraints / parameters**	2718/0/143	
**Refinement method**	Least squares	**Final R indices**	[all data]	R_1 = 0.0185, wR_2 = 0.0355
**Function minimized**	Σ w(F_o^2 - Fc^2)^2		I>=2σ (I)	R1 = 0.0156, wR2 = 0.0347
**Goodness-of-fit on F^2**	1.065	**Weighting scheme**	w=1/[σ2(Fo^2)+(0.0182P)^2+ 0.0507P]	
**Largest diff. peak and hole [e Å^{-3}]**	0.46/-1.22		where P=(F_o^2+2F_c^2)/3	

Bis(η^5-cyclopentadienyl)[2-(4-chlorophenyl)-3-(oxo-κO)-4(H)-chromen-4-oato-κO]tungsten(IV) hexafluorophosphate, **3**.

Table S7: Sample and crystal data of compound 3.

**Chemical formula**	C28H24ClF6O4PW	**Crystal system**	monoclinic	
**Formula weight [g/mol]**	788.74	**Space group**	$P2_1/n$	
**Temperature [K]**	100.0	**Z**	4	
**Measurement method**	\Φ and \ω scans	**Volume [Å^3]**	2687.6(7)	
**Radiation (Wavelength [Å])**	MoKα (λ = 0.71073)	**Unit cell dimensions [Å] and [°]**	11.5665(17))	90
**Crystal size / [mm^3]**	0.1 × 0.09 × 0.02		15.593(2)	99.365(7)
**Crystal habit**	clear brown plate		15.103(2)	90
**Density (calculated) / [g/cm^3]**	1.949	**Absorption coefficient / [mm^{-1}]**	4.534	
**Abs. correction Tmin**	0.5993	**Abs. correction Tmax**	0.7452	
**Abs. correction type**	multiscan	**F(000) [e$^-$]**	1536.0	

Table S8: Data collection and structure refinement of compound 3.

**Index ranges**	-13 ≤ h ≤ 13, -18 ≤ k ≤ 18, -17 ≤ l ≤ 18	**Theta range for data collection [°]**	4.422 to 50.792	
**Reflections number**	33217	**Data / restraints / parameters**	4860/24/372	
**Refinement method**	Least squares	**Final R indices**	all data	R1 = 0.1453, wR2 = 0.1280
**Function minimized**	$\Sigma w(F_o^2 - F_c^2)^2$		I>2σ(I)	R1 = 0.0592, wR2 = 0.1023
**Goodness-of-fit on F^2**	0.966	**Weighting scheme**	w=1/[σ2(Fo^2)+(0.0533P)^2]	
**Largest diff. peak and hole [e Å^{-3}]**	1.35/-1.80		where $P=(F_o^2+2F_c^2)/3$	

Bis(η[5]-cyclopentadienyl)[2-(4-chlorophenyl)-3-(oxo-κO)-4(H)-chromen-4-thioato-κS]tungsten(IV) hexafluorophosphate, **4**.

Table S9: Sample and crystal data of compound 4.

**Chemical formula**	C28H24ClF6O3PSW	**Crystal system**	monoclinic	
**Formula weight [g/mol]**	804.80	**Space group**	$P2_1/n$	
**Temperature [K]**	100.0	**Z**	4	
**Measurement method**	\Φ and \ω scans	**Volume [Å^3]**	2749.9(2)	
**Radiation (Wavelength [Å])**	MoKα (λ = 0.71073)	**Unit cell dimensions [Å] and [°]**	11.4548(5)	90
**Crystal size / [mm^3]**	0.2 × 0.1 × 0.08		15.8196(7)	98.874(2)
**Crystal habit**	clear brown block		15.3591(7)	90
**Density (calculated) / [g/cm^3]**	1.944	**Absorption coefficient / [mm^{-1}]**	4.504	
**Abs. correction Tmin**	0.5497	**Abs. correction Tmax**	0.7460	
**Abs. correction type**	multiscan	**F(000) [e$^-$]**	1568.0	

Table S10: Data collection and structure refinement of compound 4.

**Index ranges**	-16 ≤ h ≤ 16, -22 ≤ k ≤ 22, -21 ≤ l ≤ 21	**Theta range for data collection [°]**	4.426 to 60.558	
**Reflections number**	191709	**Data / restraints / parameters**	8120/1/391	
**Refinement method**	Least squares	**Final R indices**	all data	R1 = 0.0251, wR2 = 0.0499
**Function minimized**	Σ w(F_o^2 - F_c^2)^2		I>2σ(I)	R1 = 0.0200, wR2 = 0.0464
**Goodness-of-fit on F^2**	1.070	**Weighting scheme**	w=1/[σ2(Fo^2)+(0.0186P)^2+ 3.9570P]	
**Largest diff. peak and hole [e Å^{-3}]**	1.15/-0.78		where P=(F_o^2+2F_c^2)/3	

Bis(η^5-cyclopentadienyl)[2-(carboxylato-κO)-pyridine-κS]tungsten(IV) hexafluorophosphate, **5**.

Table S11: Sample and crystal data of compound 5.

**Chemical formula**	C16H14F6NO2PW	**Crystal system**	orthorhombic	
**Formula weight [g/mol]**	581.10	**Space group**	*Pca2₁*	
**Temperature [K]**	100.0	**Z**	4	
**Measurement method**	\Φ and \ω scans	**Volume [Å³]**	1685.40(15)	
**Radiation (Wavelength [Å])**	MoKα (λ = 0.71073)	**Unit cell dimensions [Å] and [°]**	12.3684(6)	90
**Crystal size / [mm³]**	0.388 × 0.267 × 0.026		9.9666(5)	90
**Crystal habit**	clear brown plate		13.6723(7)	90
**Density (calculated) / [g/cm³]**	2.290	**Absorption coefficient / [mm⁻¹]**	14.334	
**Abs. correction Tmin**	0.3492	**Abs. correction Tmax**	0.7536	
**Abs. correction type**	multiscan	**F(000) [e⁻]**	1104.0	

Table S12: Data collection and structure refinement of compound 5.

**Index ranges**	-14 ≤ h ≤ 14, -11 ≤ k ≤ 11, -16 ≤ l ≤ 14	**Theta range for data collection [°]**	6.464 to 130.124	
**Reflections number**	33349	**Data / restraints / parameters**	2841/1/245	
**Refinement method**	Least squares	**Final R indices**	all data	R1 = 0.0381, wR2 = 0.1051
**Function minimized**	Σ w(F_o^2 - F_c^2)^2		I>2σ(I)	R1 = 0.0371, wR2 = 0.0997
**Goodness-of-fit on F^2**	1.107	**Weighting scheme**	w=1/[σ2(Fo^2)+(0.0748P)^2+ 1.5724P]	
**Largest diff. peak and hole [e Å⁻³]**	3.51/-1.43		where P=(F_o^2+2F_c^2)/3	

Bis(η^5-cyclopentadienyl)[1,2-dimethyl-3-(oxo-κO)-4-(1H)-pyridonato-κO]tungsten(IV) hexafluorophosphate, **6**.

Table S13: Sample and crystal data of compound 6.

**Chemical formula**	C17H18F6NO2PW	**Crystal system**	orthorhombic	
**Formula weight [g/mol]**	597.14	**Space group**	$Pna2_1$	
**Temperature [K]**	100.0	**Z**	4	
**Measurement method**	\Φ and \ω scans	**Volume [Å^3]**	1782.06(14)	
**Radiation (Wavelength [Å])**	MoKα (λ = 0.71073)	**Unit cell dimensions [Å] and [°]**	20.8991(10)	90
**Crystal size / [mm^3]**	0.15 × 0.13 × 0.04		7.9947(4)	90
**Crystal habit**	clear brown plate		10.6658(4)	90
**Density (calculated) / [g/cm^3]**	2.226	**Absorption coefficient / [mm^{-1}]**	6.646	
**Abs. correction Tmin**	0.5250	**Abs. correction Tmax**	0.7460	
**Abs. correction type**	multiscan	**F(000) [e$^-$]**	1144.0	

Table S14: Data collection and structure refinement of compound 6.

**Index ranges**	-29 ≤ h ≤ 29, -11 ≤ k ≤ 11, -15 ≤ l ≤ 14	**Theta range for data collection [°]**	1.948 to 60.322	
**Reflections number**	115981	**Data / restraints / parameters**	5108/1/280	
**Refinement method**	Least squares	**Final R indices**	all data	R1 = 0.0139, wR2 = 0.0311
**Function minimized**	Σ w(F_o^2 - F_c^2)^2		I>2σ(I)	R1 = 0.0131, wR2 = 0.0307
**Goodness-of-fit on F^2**	1.045	**Weighting scheme**	w=1/[σ^2(F_o^2)+(0.0142P)^2+ 0.4852P]	
**Largest diff. peak and hole [e Å^{-3}]**	1.36/-0.69		where P=(F_o^2+2F_c^2)/3	

Bis(η^5-cyclopentadienyl)[2-methyl-5-(oxo-κO)-4-(1H)-pyran-4-ato-κO] tungsten(IV) hexafluorophosphate, **7**.

Table S15: Sample and crystal data of compound **7**.

**Chemical formula**	$C_{16}H_{15}F_6O_3PW$	**Crystal system**	orthorhombic	
**Formula weight [g/mol]**	584.10	**Space group**	$Pmn2_1$	
**Temperature [K]**	100	**Z**	2	
**Measurement method**	\Φ and \ω scans	**Volume [Å³]**	854.39(7)	
**Radiation (Wavelength [Å])**	MoKα (λ = 0.71073)	**Unit cell dimensions [Å] and [°]**	8.5280(4)	90
**Crystal size / [mm³]**	0.14 × 0.14 × 0.04		6.8174(3)	90
**Crystal habit**	Clear brown block		14.6957(6)	90
**Density calculated / [g/cm³]**	2.270	**Absorption coefficient / [mm⁻¹]**	6.931	
**Abs. correction type**	multiscan	**F(000) [e⁻]**	556.0	

Table S16: Data collection and structure refinement of compound **7**.

**Index ranges**	-10 ≤ h ≤ 10, -8 ≤ k ≤ 8, -17 ≤ l ≤ 17	**Theta range for data collection [°]**	5.522 to 50.692	
**Reflections number**	6904	**Data / restraints / parameters**	1666/1/143	
**Refinement method**	Least squares	**Final R indices**	[all data]	R_1 = 0.0283, wR_2 = 0.0647
**Function minimized**	Σ w(F_o^2 - Fc^2)^2		I>=2σ (I)	R_1 = 0.0269, wR_2 = 0.0642
**Goodness-of-fit on F^2**	1.059	**Weighting scheme**	$w=1/[\sigma^2(F_o^2+(0.0383P)^2+1.1113P]$	
**Largest diff. peak and hole [e Å⁻³]**	2.08/-0.65		where $P=(F_o^2+2F_c^2)/3$	

Bis(η[5]-cyclopentadienyl)[2-methyl-5-(oxo-κO)-pyran-4-(1H)-thionato-κS] tungsten(IV) hexafluorophosphate, **8.**

Table S17: Sample and crystal data of compound **8**.

**Chemical formula**	$C_{16}H_{15}F_6O_2PSW$	**Crystal system**	monoclinic	
**Formula weight [g/mol]**	600.16	**Space group**	$P2_1/c$	
**Temperature [K]**	100	**Z**	4	
**Measurement method**	\Φ and \ω scans	**Volume [Å^3]**	1730.5(6)	
**Radiation (Wavelength [Å])**	MoKα (λ =0.71073)	**Unit cell dimensions [Å] and [°]**	7.6463(16)	90
**Crystal size / [mm^3]**	0.3 × 0.2 × 0.01		20.383(4)	93.003(7)
**Crystal habit**	clear brown block		11.118(2)	90
**Density calculated / [g/cm^3]**	2.304	**Absorption coefficient / [mm^{-1}]**	6.960	
**Abs. correction type**	multiscan	**F(000) [e$^-$]**	1144.0	

Table S18: Data collection and structure refinement of compound **8**.

**Index ranges**	-8 ≤ h ≤ 9, -24 ≤ k ≤ 24, -13 ≤ l ≤ 13	**Theta range for data collection [°]**	5.334 to 50.698	
**Reflections number**	18772	**Data / restraints / parameters**	3111/60/245	
**Refinement method**	Least squares	**Final R indices**	[all data]	R_1 = 0.0859, wR_2 = 0.1671
**Function minimized**	Σ w(F_o^2 - Fc^2)^2		I>=2σ (I)	R_1 = 0.0671, wR_2 = 0.1546
**Goodness-of-fit on F^2**	1.164	**Weighting scheme**	w=1/[σ^2+(F_o^2+(0.0175P)^2+93.7428P]	
**Largest diff. peak and hole [e Å^{-3}]**	2.13/-2.36		where P=(F_o^2+2F_c^2)/3	

Bis(η^5-cyclopentadienyl)[2-ethyl-3-(oxo-κO)-4-(1*H*)-pyran-4-ato-κO] tungsten(IV) hexafluorophosphate, **9**.

Table S19: Sample and crystal data of compound **9**.

**Chemical formula**	$C_{17}H_{17}F_6O_3PW$	**Crystal system**	orthorhombic	
**Formula weight [g/mol]**	598.12	**Space group**	Pnma	
**Temperature [K]**	100	**Z**	4	
**Measurement method**	\Φ and \ω scans	**Volume [Å^3]**	1782.58(12)	
**Radiation (Wavelength [Å])**	MoKα (λ = 0.71073)	**Unit cell dimensions [Å] and [°]**	13.7943(4)	90
**Crystal size / [mm^3]**	0.12 × 0.11 × 0.03		8.3019(3)	90
**Crystal habit**	clear brown block		15.5658(7)	90
**Density calculated / [g/cm^3]**	2.229	**Absorption coefficient / [mm^{-1}]**	6.647	
**Abs. correction type**	multiscan	**F(000) [e$^-$]**	1144.0	

Table S20: Data collection and structure refinement of compound **9**.

**Index ranges**	-10 ≤ h ≤ 16, -9 ≤ k ≤ 7, -11 ≤ l ≤ 18	**Theta range for data collection [°]**	5.562 to 50.688	
**Reflections number**	4136	**Data / restraints / parameters**	1732/0/149	
**Refinement method**	Least squares	**Final R indices**	[all data]	R_1 = 0.0339, wR_2 =0.0577
**Function minimized**	Σ w(F_o^2 - Fc^2)^2		I>=2σ (I)	R_1 =0.0252, wR_2 =0.0548
**Goodness-of-fit on F^2**	1.048	**Weighting scheme**	w=1/[σ^2+(F_o^2+(0.0228P)^2+5.4016P]	
**Largest diff. peak and hole [e Å^{-3}]**	1.10/-1.11		where P=(F_o^2+2F_c^2)/3	

Bis(η5-cyclopentadienyl)[2-ethyl-3-(oxo-κO)-4-(1H)-pyran-4-ato-κS] tungsten(IV) hexafluorophosphate, **10**.

Table S21: Sample and crystal data of compound **10**.

**Chemical formula**	$C_{17}H_{17}F_6O_2PSW$	**Crystal system**	triclinic	
**Formula weight [g/mol]**	614.19	**Space group**	P-1	
**Temperature [K]**	100	**Z**	2	
**Measurement method**	\Φ and \ω scans	**Volume [Å^3]**	905.88(6)	
**Radiation (Wavelength [Å])**	MoKα (λ = 0.71073)	**Unit cell dimensions [Å] and [°]**	7.6662(3)	82.659(2)
**Crystal size / [mm^3]**	0.14 × 0.1 × 0.04		10.7376(4)	79.687(2)
**Crystal habit**	clear brown block		11.3297(4)	83.341(2)
**Density calculated / [g/cm^3]**	2.252	**Absorption coefficient / [mm^{-1}]**	6.650	
**Abs. correction type**	multiscan	**F(000) [e$^-$]**	588.0	

Table S22: Data collection and structure refinement of compound **10**.

**Index ranges**	-10 ≤ h ≤ 10, -15 ≤ 5, -15 ≤ l ≤ 15	**Theta range for data collection [°]**	5.018 to 60.416	
**Reflections number**	50145	**Data / restraints / parameters**	5295/0/254	
**Refinement method**	Least squares	**Final R indices**	[all data]	R_1 = 0.0181, wR_2 =0.0364
**Function minimized**	Σ w(F_o^2 - Fc^2)^2		I>=2σ (I)	R_1 =0.0165, wR_2 =0.0357
**Goodness-of-fit on**	1.031	**Weighting scheme**	w=1/[$σ^2$+(F_o^2+(0.0132P)^2+1.1500P]	
**Largest diff. peak and hole [e Å^{-3}]**	0.91/-0.88		where P=(F_o^2+2F_c^2)/3	

Cyclic Voltammetry

Due to low water-solubility of the compounds **1–10** MeCN was utilized in order to reach the desired concentration of 2 mM. All complexes exhibit a reversible one-electron process W^{IV} to W^{V}.

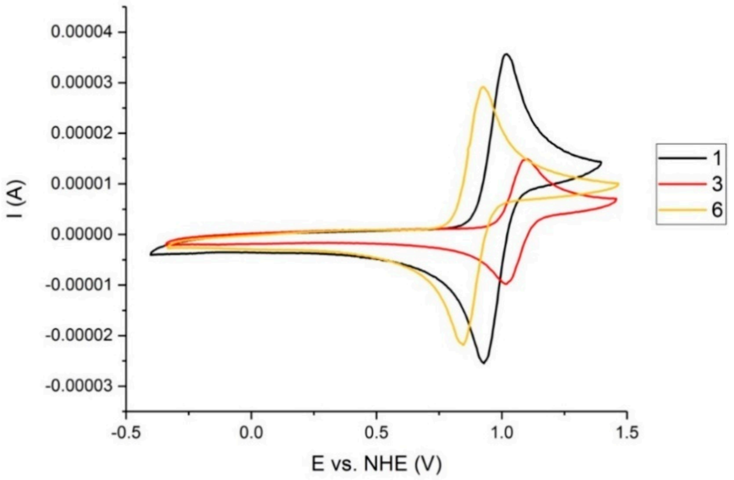

Figure S12: Cyclic Voltammogram of the (*O,O*–) chelates, compounds **1**, **3** and **6** in MeCN referenced to the NHE.

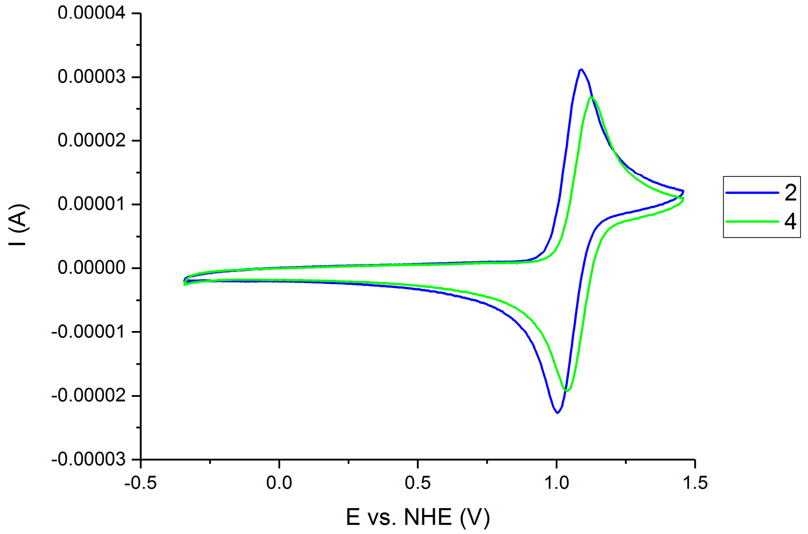

Figure S13: Cyclic Voltammogram of the (*S,O*–) chelates, compounds **2** and **4** in MeCN referenced to the NHE.

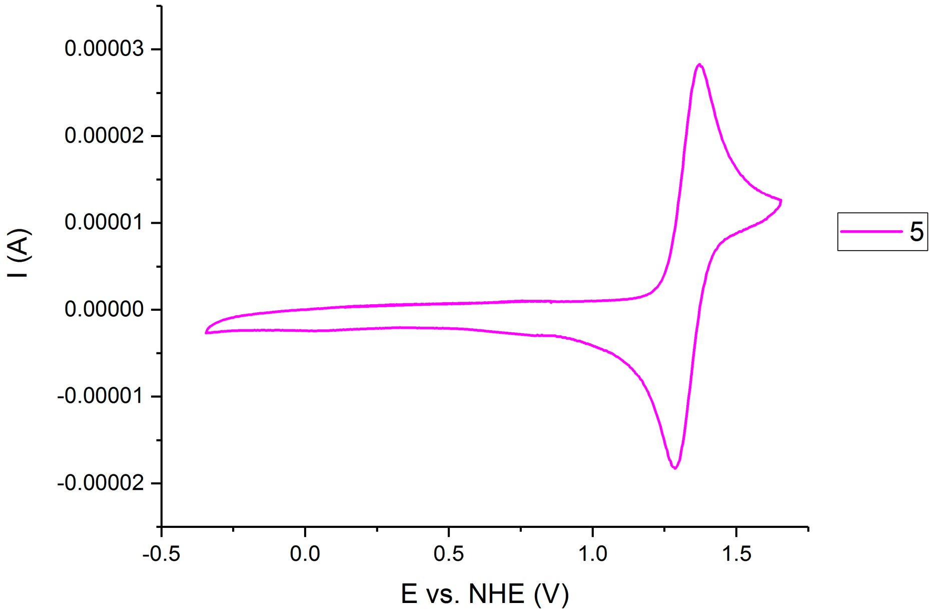

Figure S14: Cyclic Voltammogram of the (*N,O*)–chelate compound **5** in MeCN referenced to the NHE.

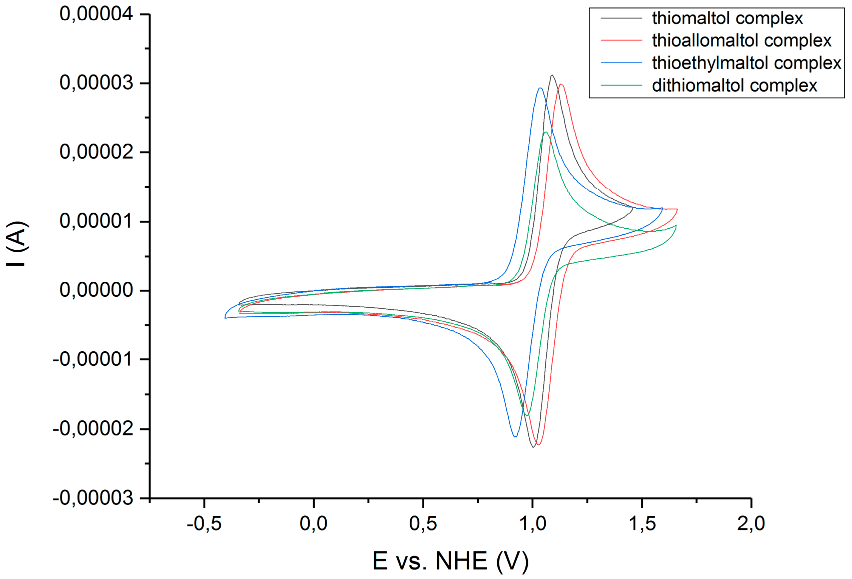

Figure S15: Cyclic Voltammogram of the (*S,O*–) chelates compounds **2**, **8**, **10** and a dithiomaltolato derivative in MeCN referenced to the NHE.

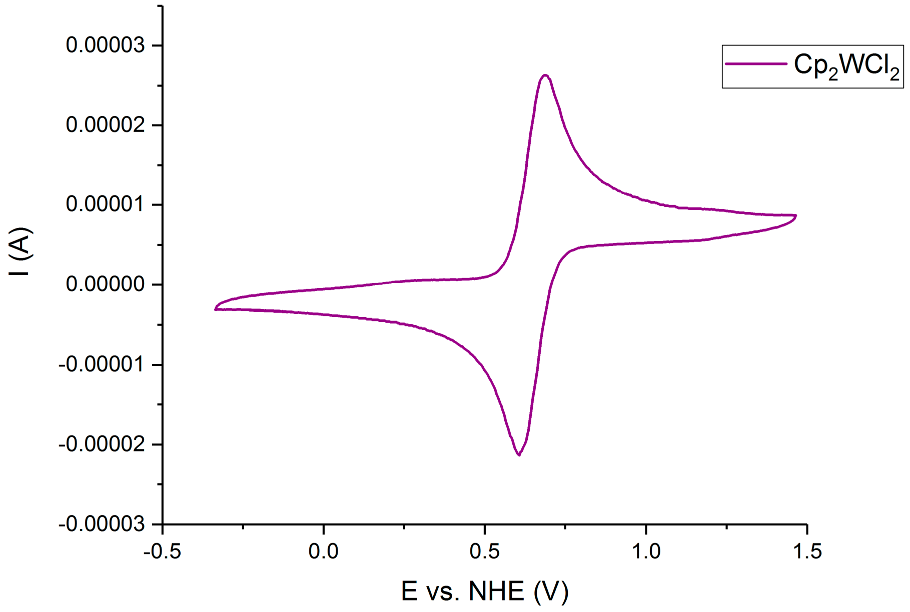

Figure S16: Cyclic Voltammogram of Cp_2WCl_2 in MeCN referenced to the NHE.

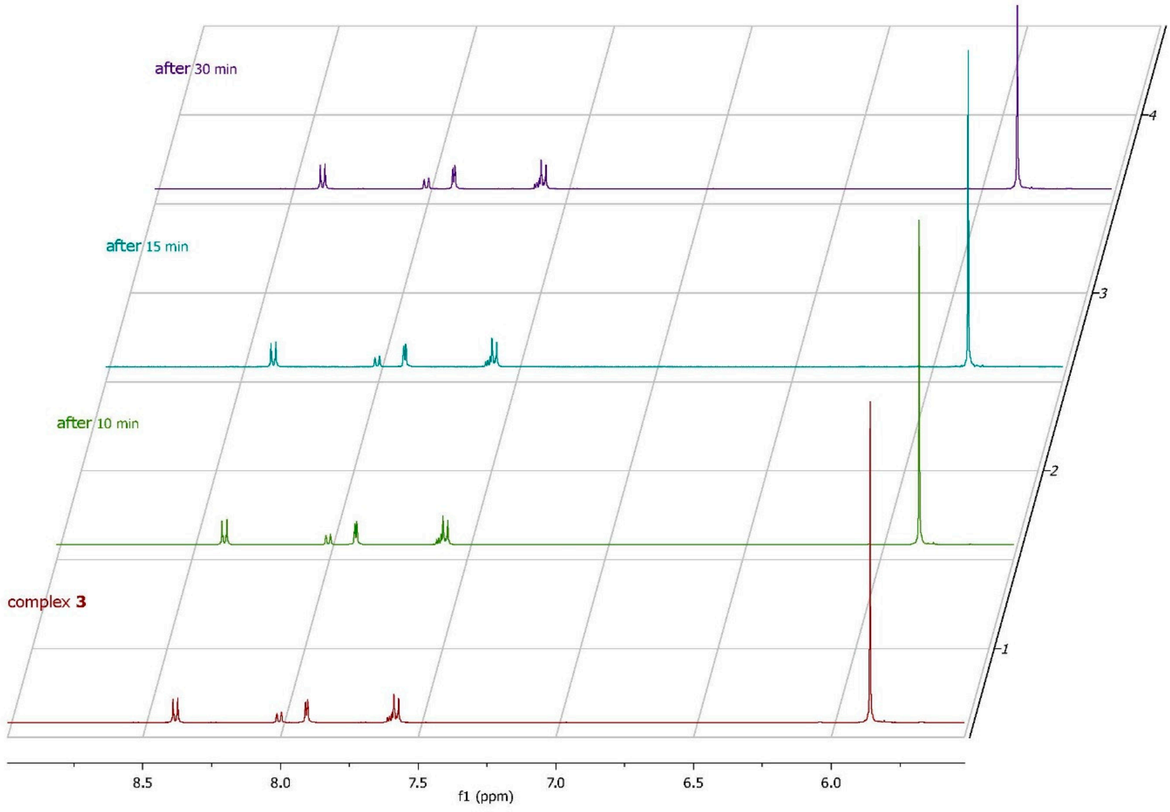

Figure S17:^{1}H NMR in MeCN of complex **3** to gauge the stability over the timespan of a cyclic voltammetry experiment.

UV/Vis spectroscopy

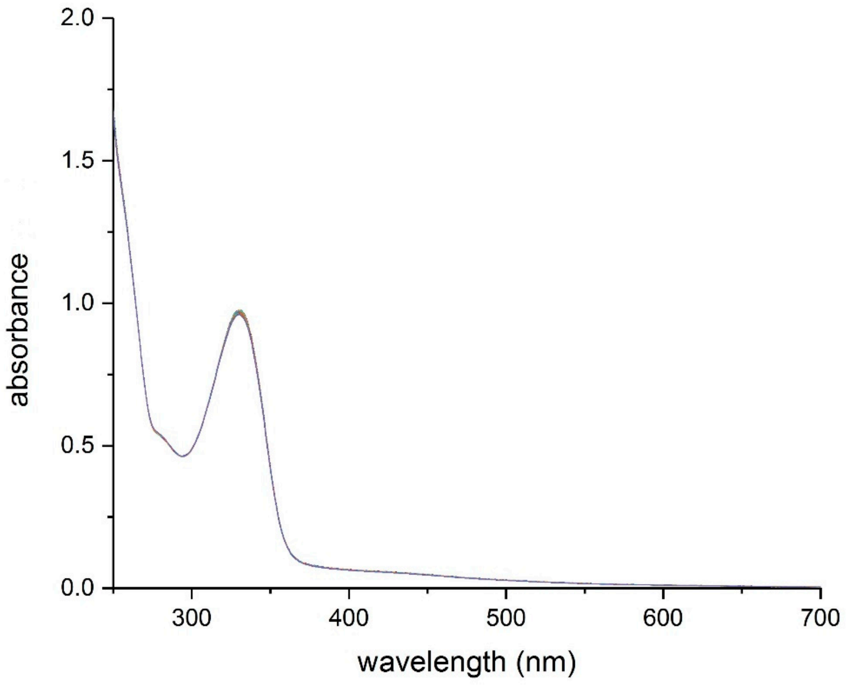

Figure S18: UV/Vis spectra of complex 1 (0.039 mM) over 24 h in PBS (1% DMSO).

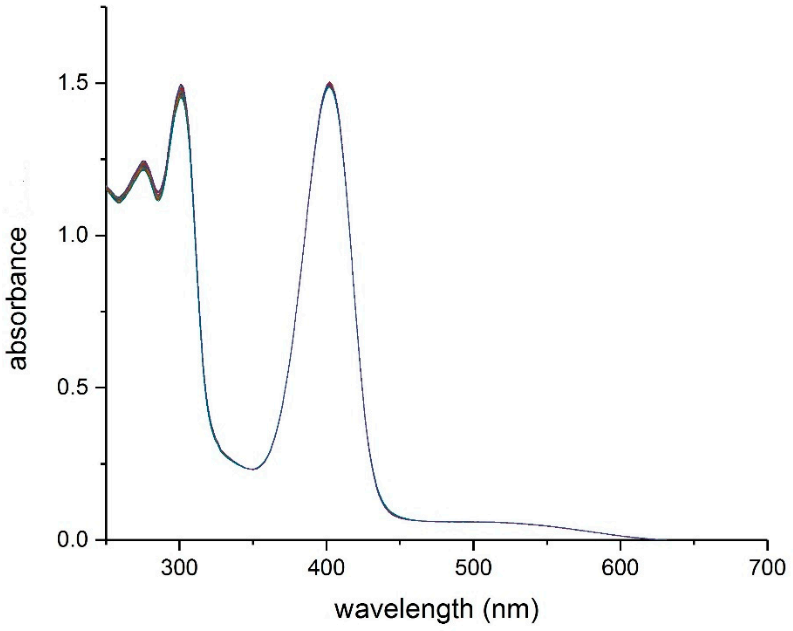

Figure S19: UV/Vis spectra of complex 2 (0.042 mM) over 24 h in PBS (1% DMSO).

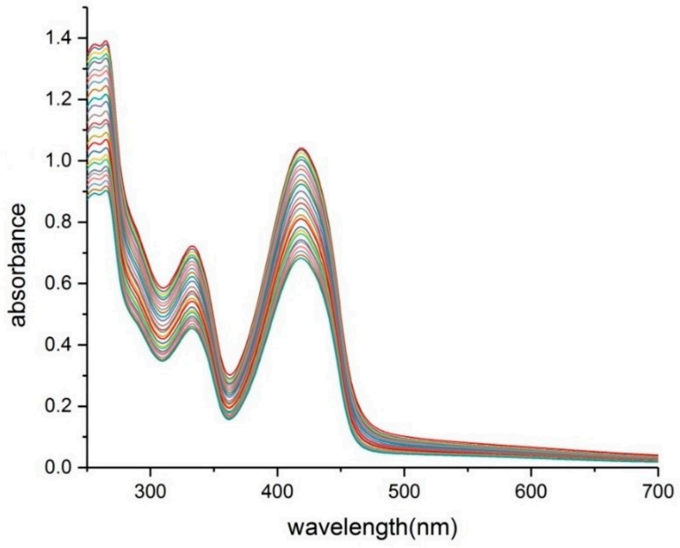

Figure S20: UV/Vis spectra of complex **3** (0.041 mM) over 24 h in PBS (1% DMSO). Precipitation over the time can be observed.

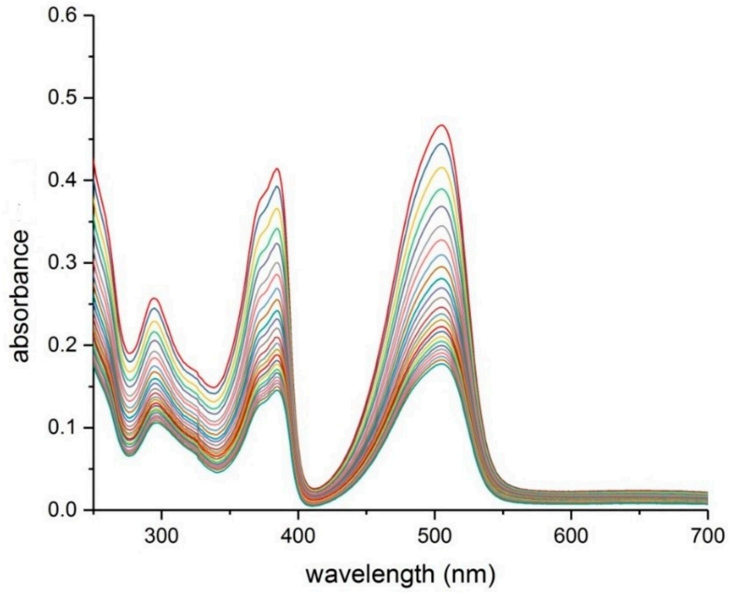

Figure S21: UV/Vis spectra of complex **4** (0.013 mM) over 24 h in PBS (1% DMSO). Precipitation over the time can be observed.

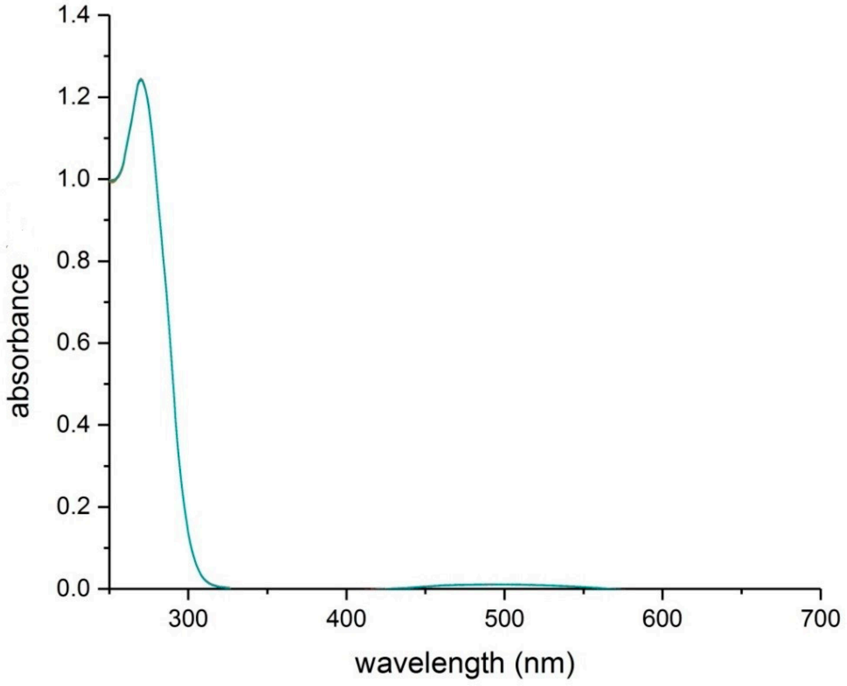

Figure S22: UV/Vis spectra of complex 5 (0.031 mM) over 24 h in PBS (1% DMSO).

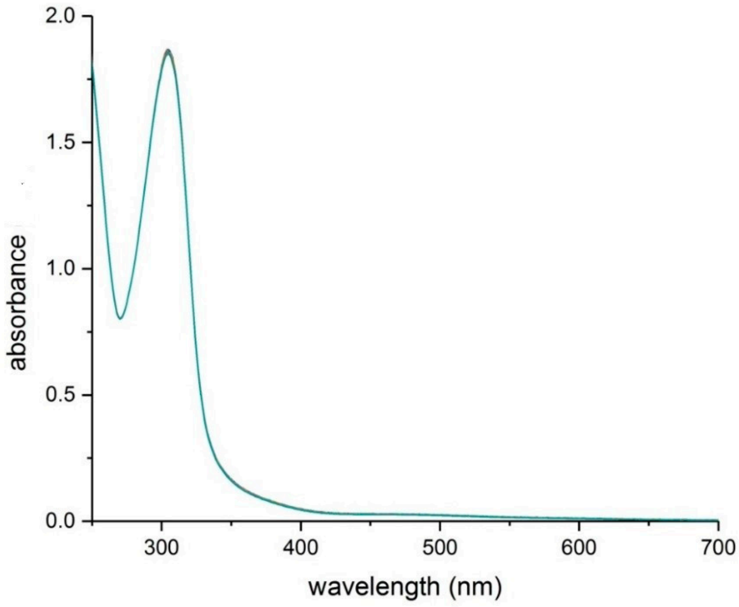

Figure S23: UV/Vis spectra of complex 6 (0.080 mM) over 24 h in PBS (1% DMSO).

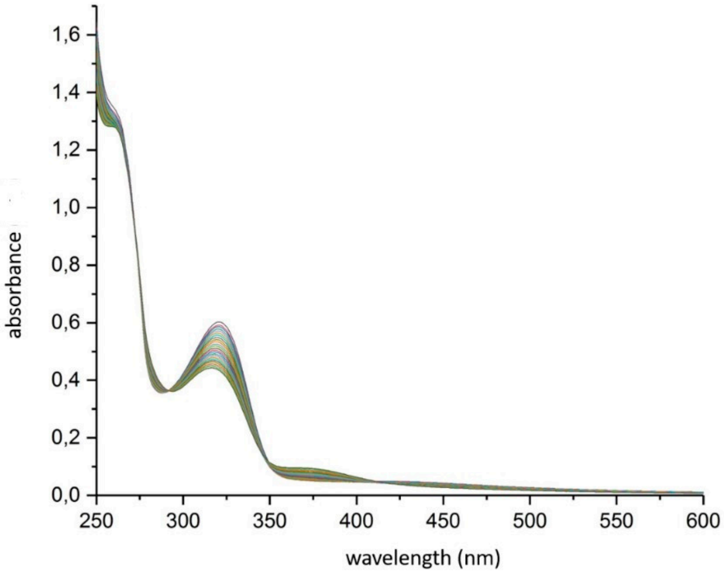

Figure S24: UV/Vis spectra of complex **7** (0.062 mM) over 24 h in PBS (1% DMSO).

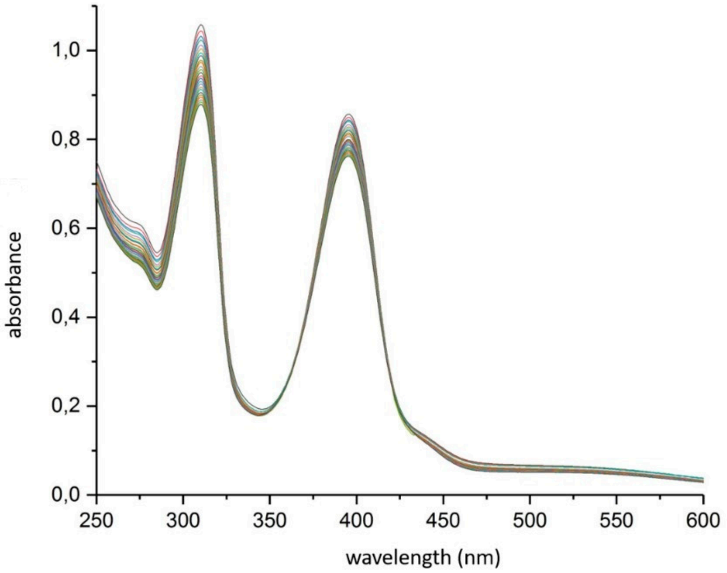

Figure S25: UV/Vis spectra of complex 8 (0.034 mM) over 24 h in PBS (1% DMSO).

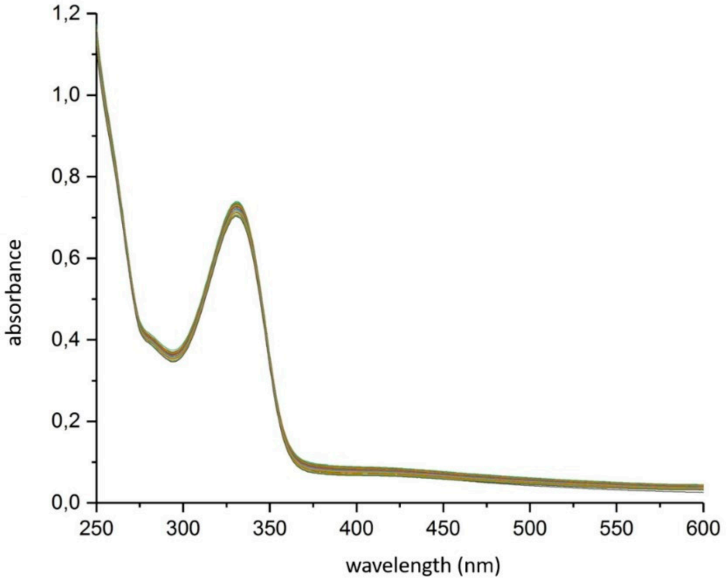

Figure S26: UV/Vis spectra of complex **9** (0.043 mM) over 24 h in PBS (1% DMSO).

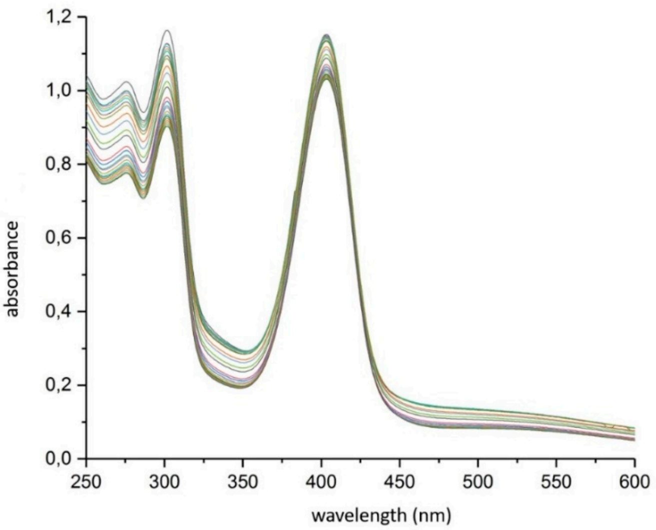

Figure S27: UV/Vis spectra of complex 10 (0.052 mM) over 24 h in PBS (1% DMSO).

Table S23: Wavelength of peak maxima and molar extinction coefficients (ε) of compounds
1–10 in 10% PBS (1% DMSO)

Compound	λ_{max} [nm] (ε [M^{-1} cm^{-1}])
**1**	330 (39049)
**2**	402 (120301), 301 (119818), 276 (99628)
**3**	256 (110446), 265 (111269), 333 (57762), 419 (83286)
**4**	505 (93412), 384 (82852), 295 (51368), 242 (100662)
**5**	305 (74304)
**6**	270 (49723)
**7**	320 (60272)
**8**	395 (85700), 310 (105823)
**9**	331 (73797)
**10**	403 (115229), 302 (116416), 276 (102373)

MTT assay

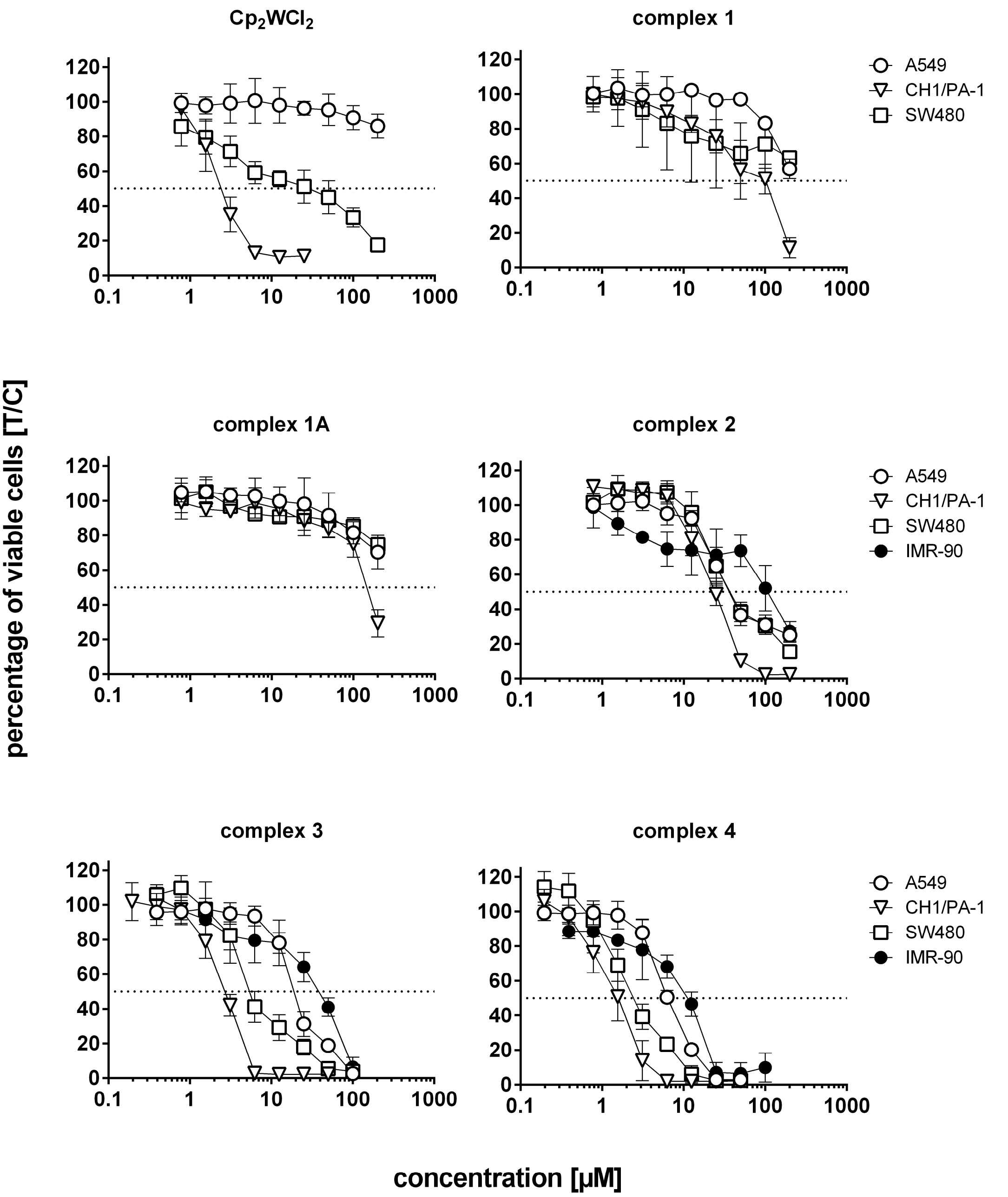

Figure S28: Concentration-effect curves of Cp_2WCl_2 and five other tungstenocenes (**1A**, **1**–**4**) in A549, CH1/PA-1 and SW480 cancer cells as well as (partially) in IMR-90 fibroblasts, obtained by the colorimetric MTT assay after 96 h incubation. Data are means ± standard deviations from at least three experiments (except for compounds that did not yield an IC_{50} value; these were tested only twice). The dashed line refers to the 50% inhibitory level relative to the untreated controls.

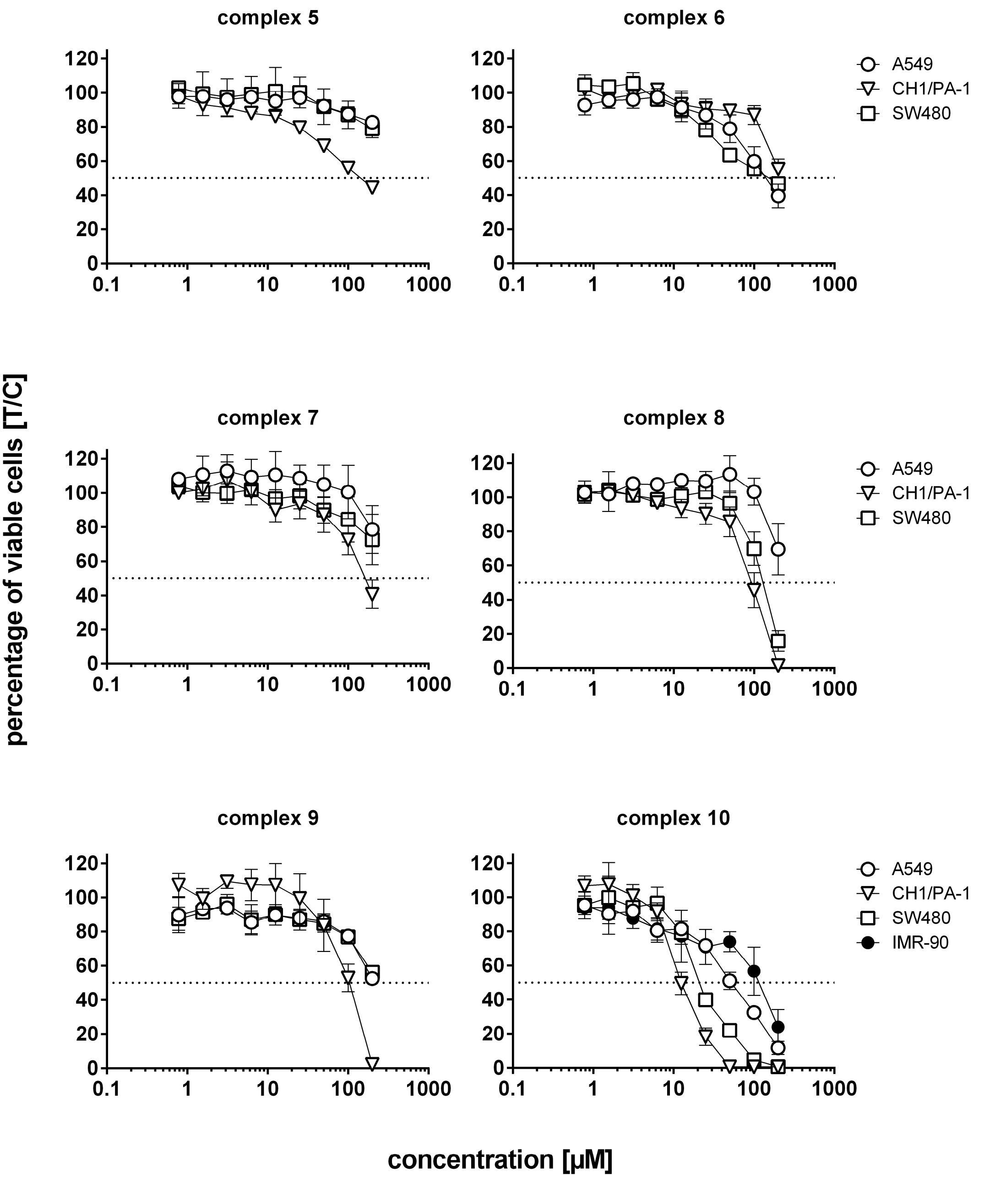

Figure S29: Concentration-effect curves of six tungstenocenes (**5**–**10**) in A549, CH1/PA-1, and SW480 cancer cells as well as (partially) in IMR-90 fibroblasts, obtained by the colorimetric MTT assay after 96 h incubation. Data are means ± standard deviations from at least three experiments (except for compounds that did not yield an IC_{50} value; these were tested only twice). The dashed line refers to the 50% inhibitory level relative to the untreated controls.

Resazurin assay

Table S24: IC_{50} values and calculated ratios of IC_{50} values obtained in 2D and 3D cultures (determined by the resazurin assay) for complexes **2**–**4** and **10** in three cell lines (HCT116, HT29 and MCF-7).

	IC_{50} [µM]								
**compound**	**HCT116**			**HT29**			**MCF-7**		
	**2D**	**3D**	**ratio 3D/2D**	**2D**	**3D**	**ratio 3D/2D**	**2D**	**3D**	**ratio 3D/2D**
**2**	98 ± 12	158 ± 28	1.6	53 ± 9	> 200	> 3.8	66 ± 12	> 200	> 3.0
**3**	9.2 ± 1.4	21 ± 4	2.3	17 ± 4	86 ± 2	5.1	6.5 ± 1.8	44 ± 1	6.8
**4**	6.0 ± 0.9	15 ± 4	2.5	5.5 ± 1.0	31 ± 6	5.6	2.7 ± 0.7	19 ± 2	7.0
**10**	42 ± 3	69 ± 12	1.6	54 ± 2	> 200	> 3.7	29 ± 4	> 200	> 6.9

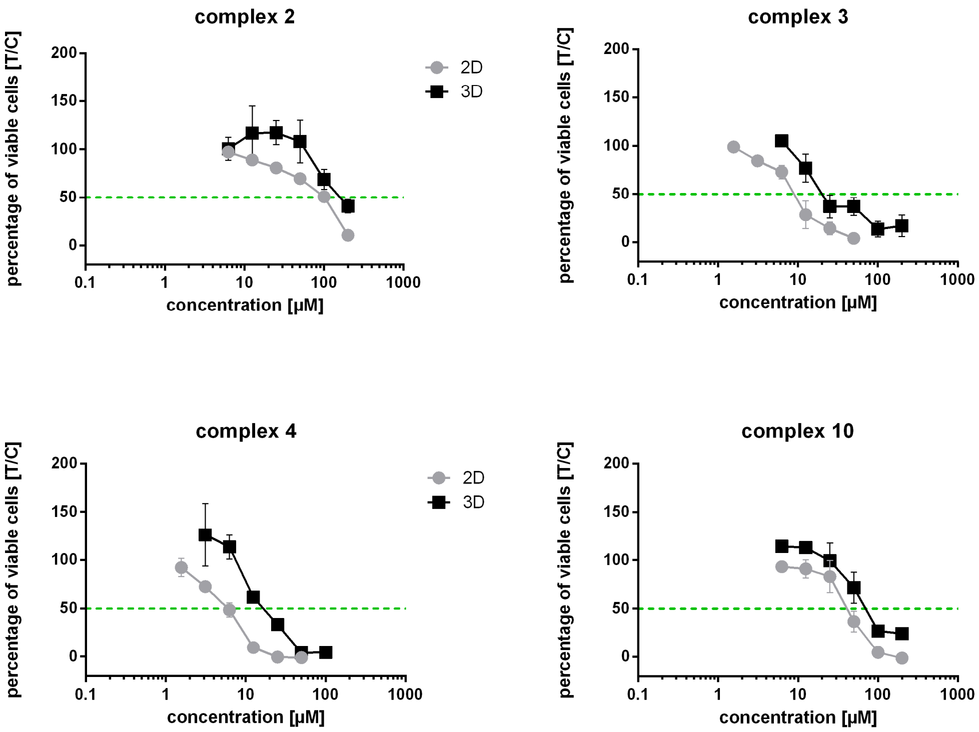

Figure S30: Concentration-effect curves of complexes **2**–**4** and **10** obtained by the resazurin assay (96 hours exposure) in monolayer (2D, grey lines) and multicellular spheroid (3D, black lines) cultures of HCT116 cells. The dashed, green line refers to the 50% inhibitory level relative to untreated controls.

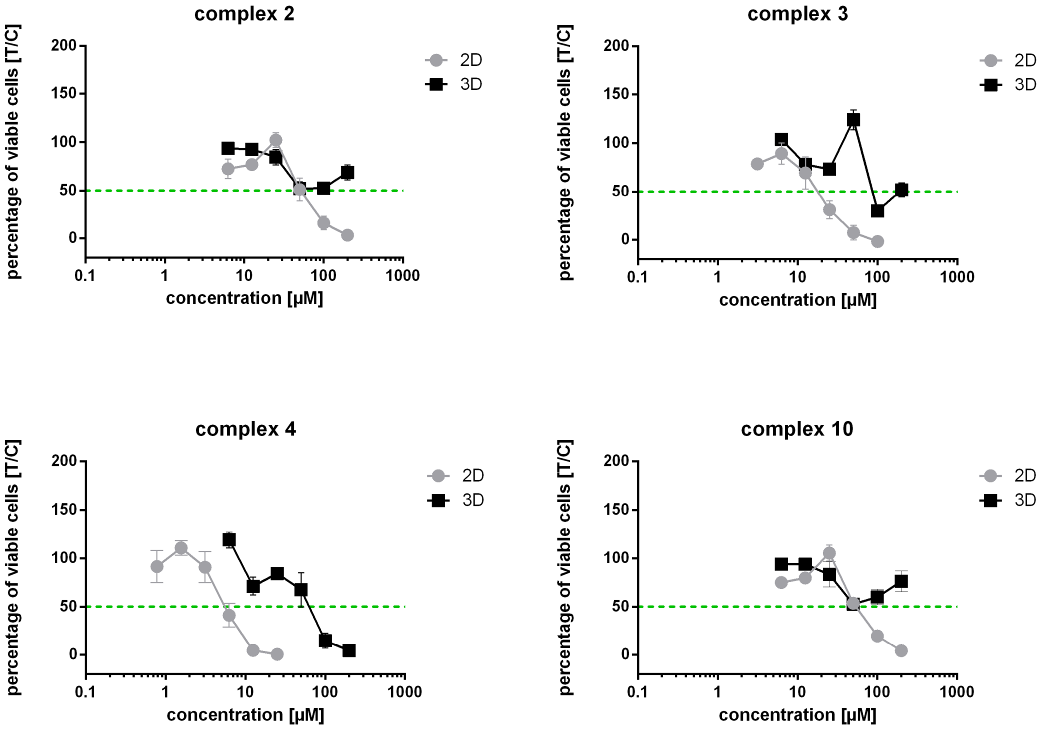

Figure S31: Concentration-effect curves of complexes **2**–**4** and **10** obtained by the resazurin assay (96 hours exposure) in monolayer (2D, grey lines) and multicellular spheroid (3D, black lines) cultures of HT29 cells. The dashed, green line refers to the 50% inhibitory level relative to untreated controls.

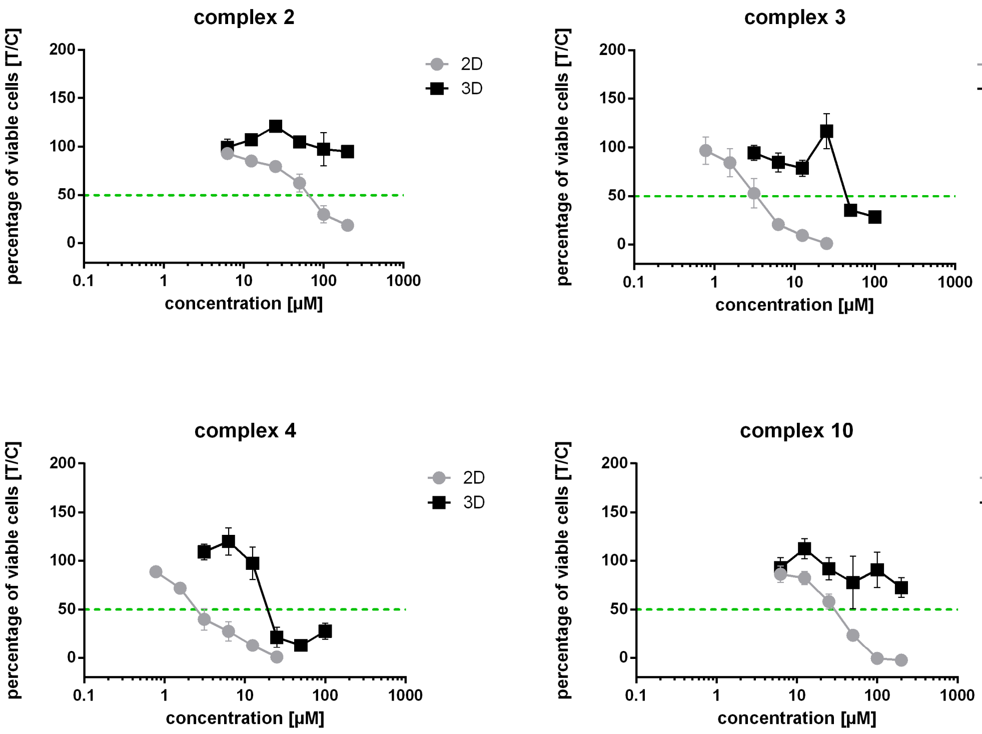

Figure S32: Concentration-effect curves of complexes **2**–**4** and **10** obtained by the resazurin assay (96 hours exposure) in monolayer (2D, grey lines) and multicellular spheroid (3D, black lines) cultures of MCF-7 cells. The dashed, green line refers to the 50% inhibitory level relative to untreated controls.

Growth curve

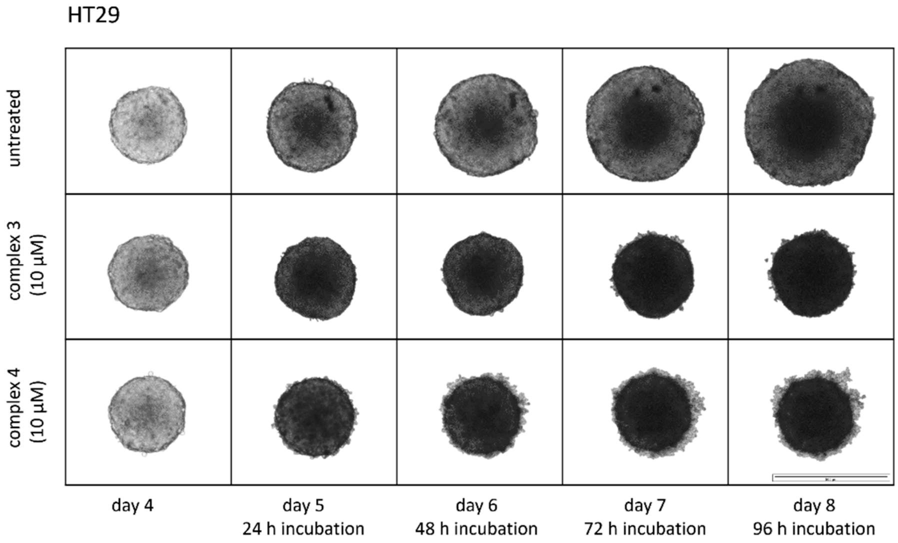

Figure S33: Time-dependent growth of HT29 spheroids upon treatment with complexes **3** and **4** (both 10 µM) and untreated control, detected for 96 hours (treatment started on day 4 after seeding). Images represent one captured spheroid out of three experiments. Scale bar = 500 µm

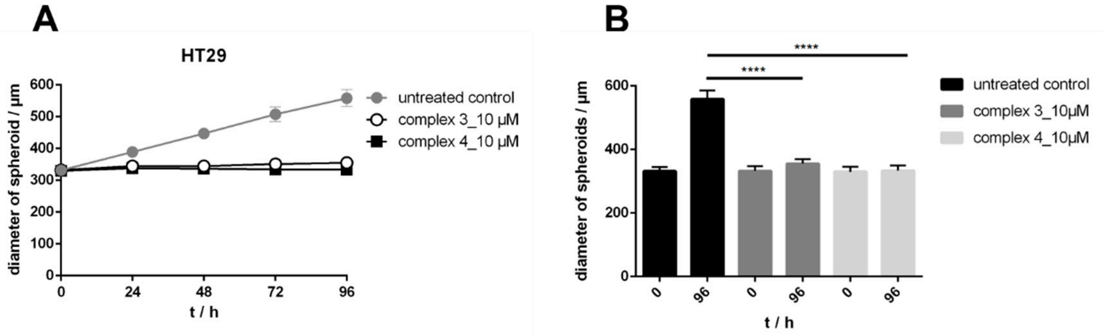

Figure S34: (**A**) Growth curves for spheroids of HT29 cells treated with complexes **3** and **4** (both 10 µM) and untreated control. Measurements started on day 4 (after seeding) with spheroid diameters of 304 ± 1 µm and were continued every 24 hours for additional 4 days. (**B**) Spheroids were initiated on day 0, treated with complexes **3** and **4** (both 10 µM) on day 4, diameter of spheroids were obtained at the beginning of the treatment (0 h) and at the end (96 h). Unpaired *t*-test with Welch's correction (n=72) was performed for statistical evaluation (**** $p < 0.0001$).

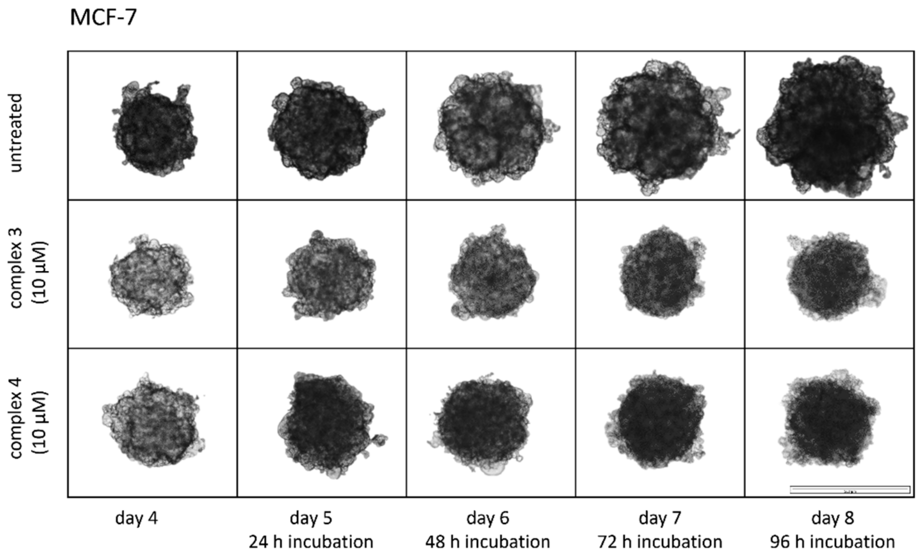

Figure S35: Time-dependent growth of MCF-7 spheroids upon treatment with complexes **3** and **4** (both 10 µM) and untreated control, detected for 96 hours (treatment started on day 4 after seeding). Images represent one captured spheroid out of three experiments. Scale bar = 500 µm

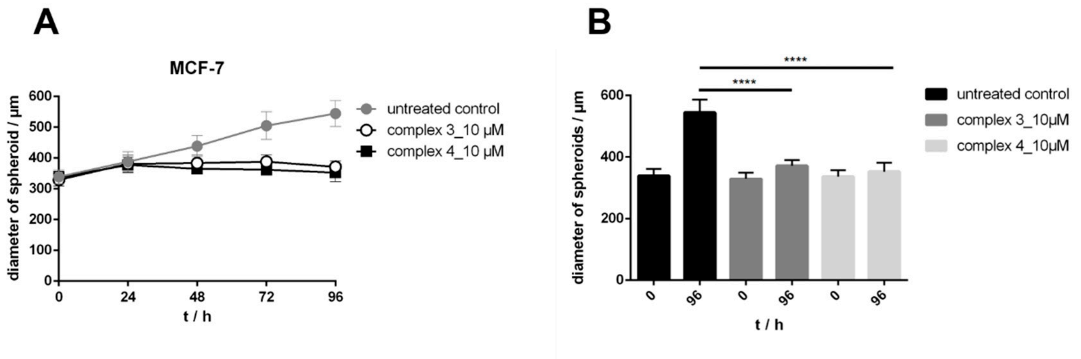

Figure S36: (**A**) Growth curves for spheroids of MCF-7 cells treated with complexes **3** and **4** (both 10 µM) and untreated control. Measurements started on day 4 (after seeding) with spheroid diameters of 304 ± 1 µm and were continued every 24 hours for additional 4 days. (**B**) Spheroids were initiated on day 0, treated with complexes **3** and **4** (both 10 µM) on day 4, diameter of spheroids were obtained at the beginning of the treatment (0 h) and at the end (96 h). Unpaired *t*-test with Welch's correction (n=72) was performed for statistical evaluation (**** $p < 0.0001$).

Immunofluorescence staining

Table S25: Statistical analysis of $KI67^+$ cells in cryo-sectioned HCT116 spheroids of untreated control and treated samples with complex **3** (20 µM) and complex **4** (15 µM) after 72 hours exposure.

	**$KI67^+$ cells**
**untreated control**	38.1 ± 7.3 %
**complex 3 (20 µM)**	15.8 ± 2.8 %
**complex 4 (15 µM)**	19.1 ± 1.3 %

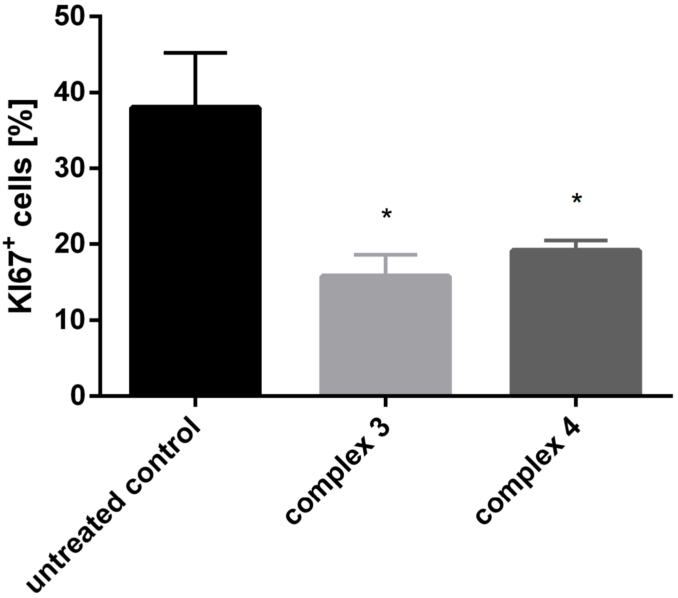

Figure S37: Quantitative analysis of $KI67^+$ cells in cryo-sectioned HCT116 spheroids, untreated or treated with complex **3** (20 µM) and complex **4** (15 µM) for 72 hours. Unpaired *t*-test with Welch's correction (n=3 for untreated control, n=4 for treated samples) was performed for statistical evaluation (* $p < 0.05$). The asterisks mark the difference relative to the untreated control, the difference between the means of complex **3** and complex **4** treated samples was found to be not significant.

Laser ablation

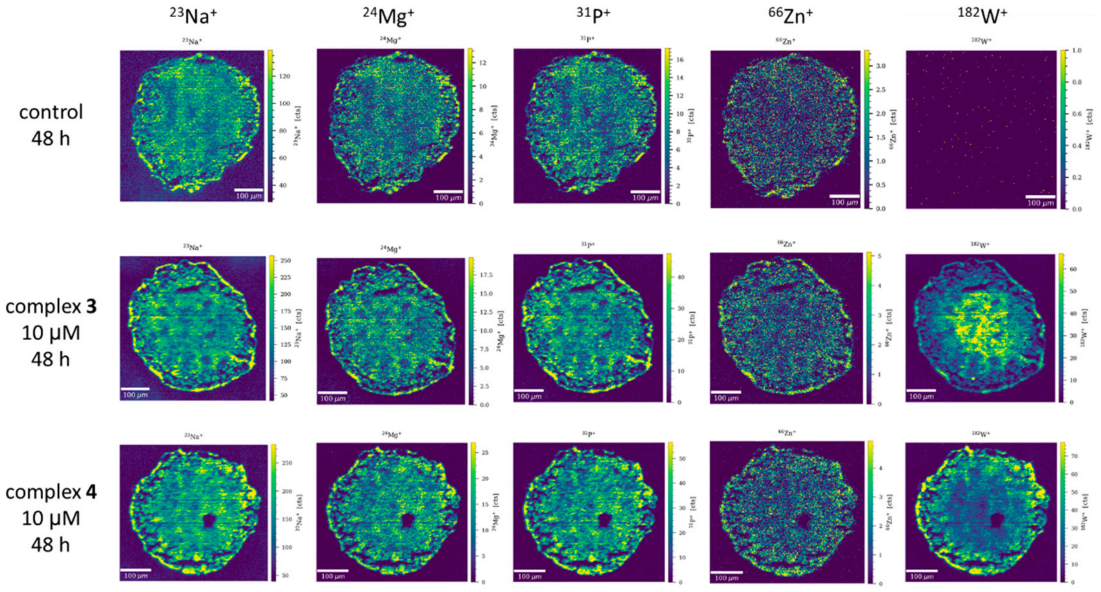

Figure S38: Signal intensity maps of $^{23}Na^+$, $^{24}Mg^+$, $^{31}P^+$, $^{66}Zn^+$ and $^{182}W^+$ obtained by LA-ICP-TOF-MS in selected HCT116 tumor spheroid sections after treatment with 10 µM of complexes **3** and **4** for 48 hours. High-resolution laser ablation images were measured with a 5 µm square mask, double oversampling and a repetition rate of 200 Hz.

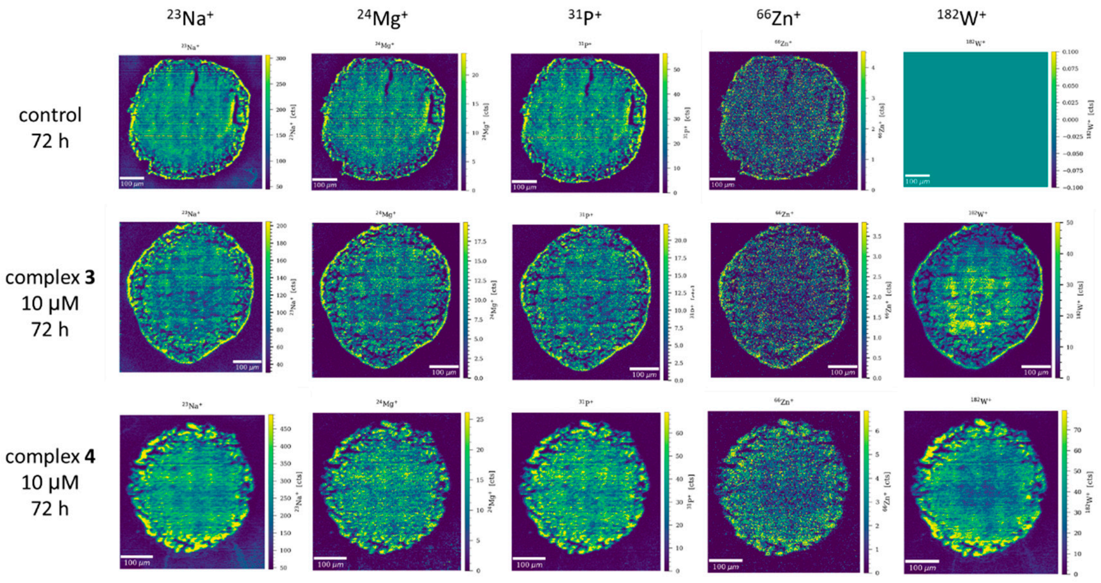

Figure S39: Signal intensity maps of $^{23}Na^+$, $^{24}Mg^+$, $^{31}P^+$, $^{66}Zn^+$ and $^{182}W^+$ obtained by LA-ICP-TOF-MS in selected HCT116 tumor spheroid sections after treatment with 10 µM of complexes **3** and **4** for 72 hours. High-resolution laser ablation images were measured with a 5 µm square mask, double oversampling and a repetition rate of 200 Hz.

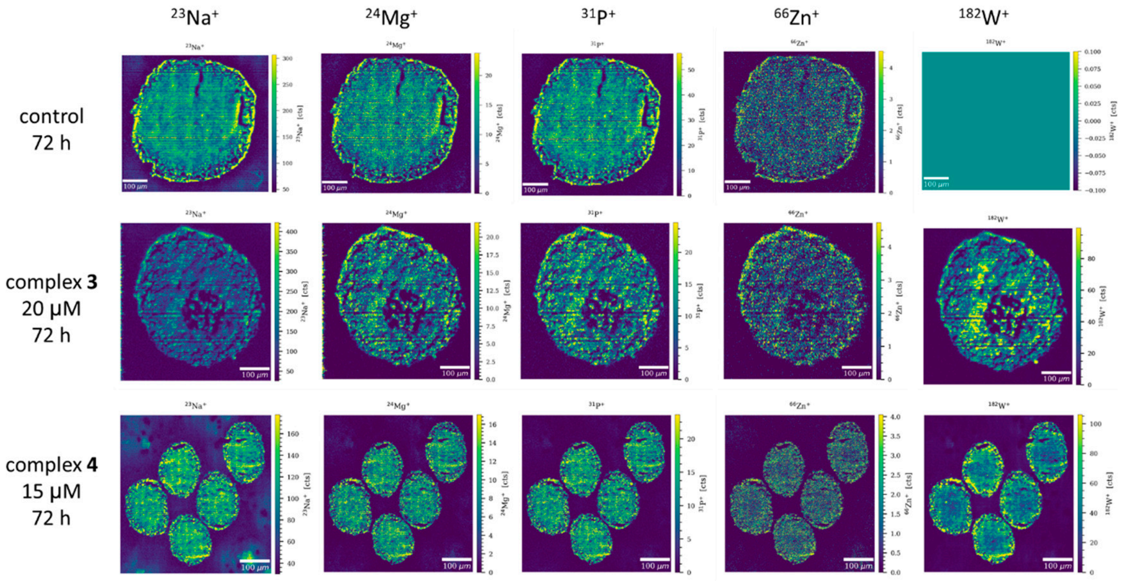

Figure S40: Signal intensity maps of $^{23}Na^{+}$, $^{24}Mg^{+}$, $^{31}P^{+}$, $^{66}Zn^{+}$ and $^{182}W^{+}$ obtained by LA-ICP-TOF-MS in selected HCT116 tumor spheroid sections after treatment with 20 µM of complex **3** and 15 µM of complex **4** for 72 hours. High-resolution laser ablation images were measured with a 5 µm square mask, double oversampling and a repetition rate of 200 Hz.

Reactive oxygen species (ROS) detection

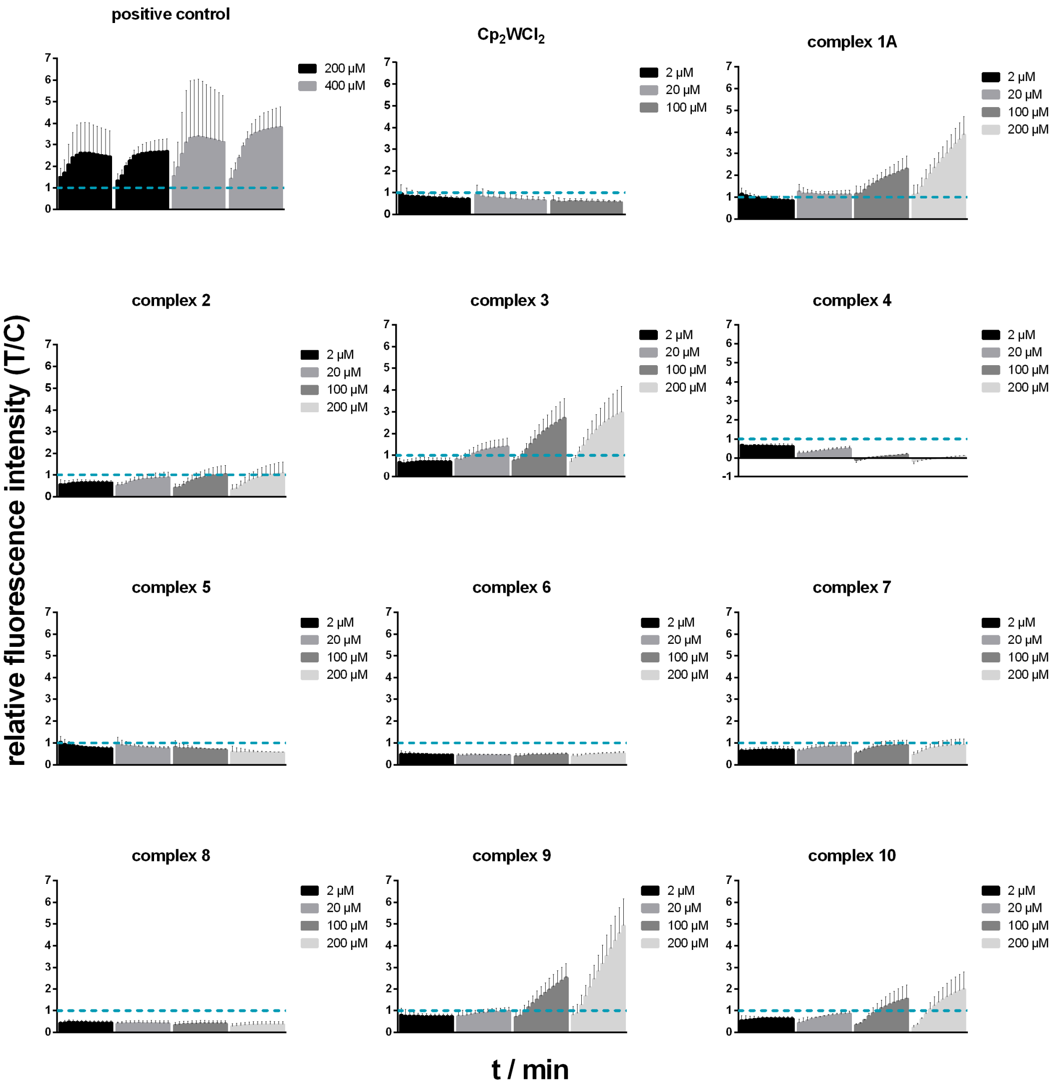

Figure S41: Time- and concentration-dependent intracellular reactive oxygen species (ROS) generation in HT29 cells. Cells were treated with tungstenocenes (2, 20, 100 and 200 µM) and tert-butyl-peroxide (200 and 400 µM) as positive control. Fluorescence intensities of DCF indicating ROS levels (relative to the untreated control) were measured with a spectrophotometer. Data from three independent experiments with n=3 wells per measurement are given as means ± standard deviations. The dashed, blue line indicates the ROS level of untreated controls to which the other values were normalized.

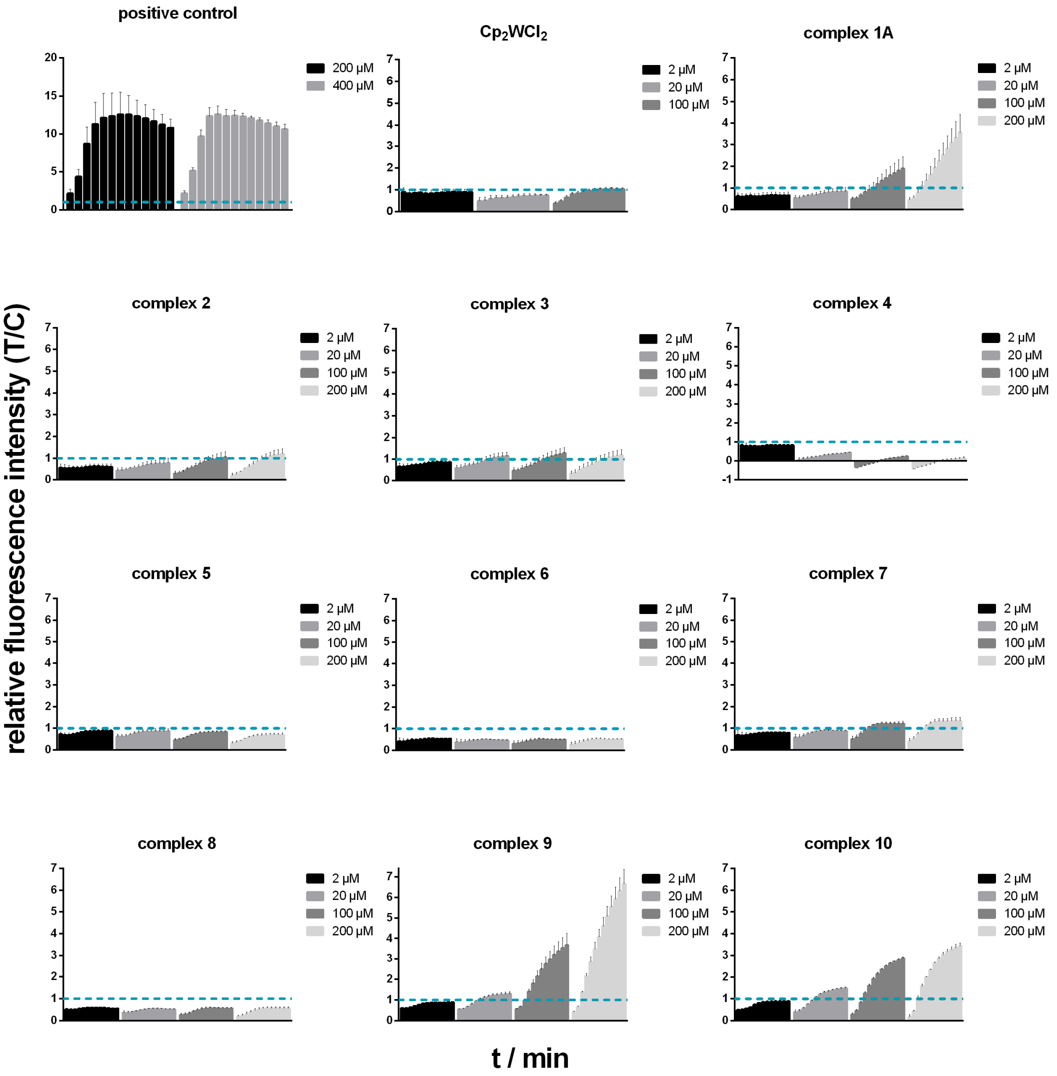

Figure S42: Time- and concentration-dependent intracellular reactive oxygen species (ROS) generation in MCF-7 cells. Cells were treated with tungstenocenes (2, 20, 100 and 200 µM) and tert-butyl-peroxide (200 and 400 µM) as positive control. Fluorescence intensities of DCF indicating ROS levels (relative to the untreated control) were measured with a spectrophotometer. Data from three independent experiments with n=3 wells per measurement are given as means ± standard deviations. The dashed, blue line indicates the ROS level of untreated controls to which the other values were normalized.

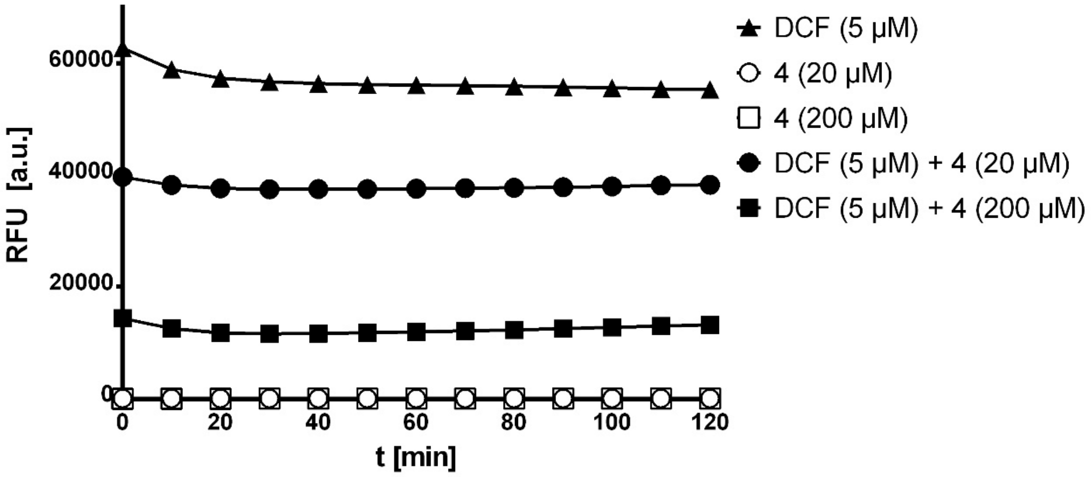

Figure S43: Quenching of DCF (2′,7′-dichlorofluorescein) fluorescence by complex **4** in a cell-free assay. DCF (5 µM) and complex **4** (20 and 200 µM, the highest concentrations used in ROS assays) were applied alone as well as in combination. Fluorescence intensity was measured over 120 min every 10 min in triplicates (standard deviations are too small to be shown on this scale).

Apoptosis studies

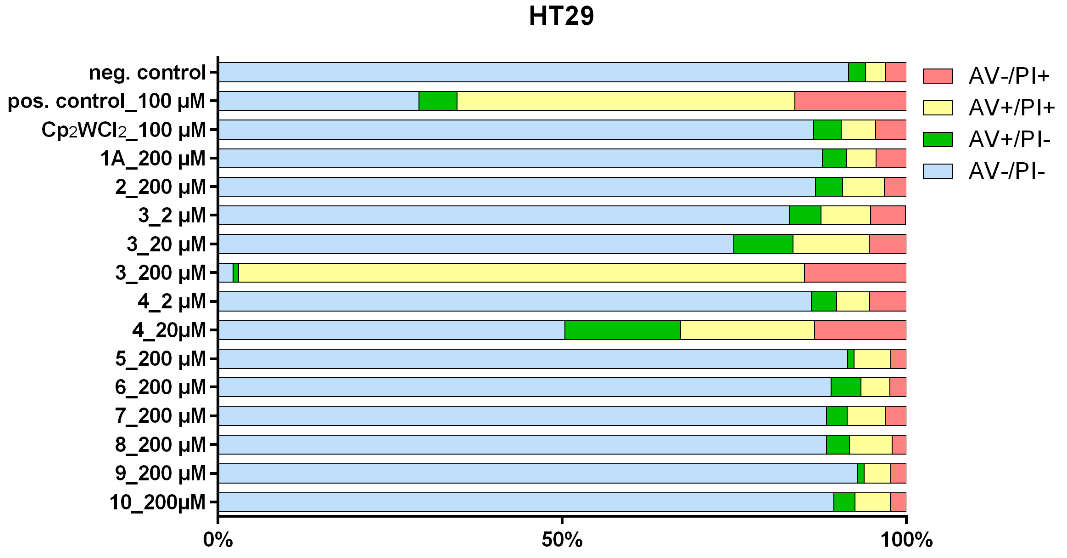

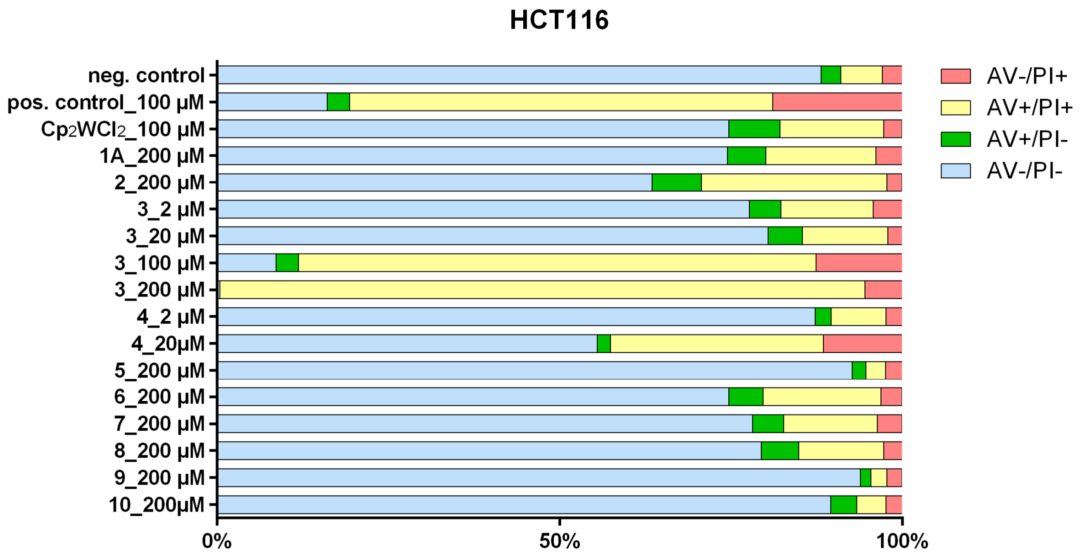

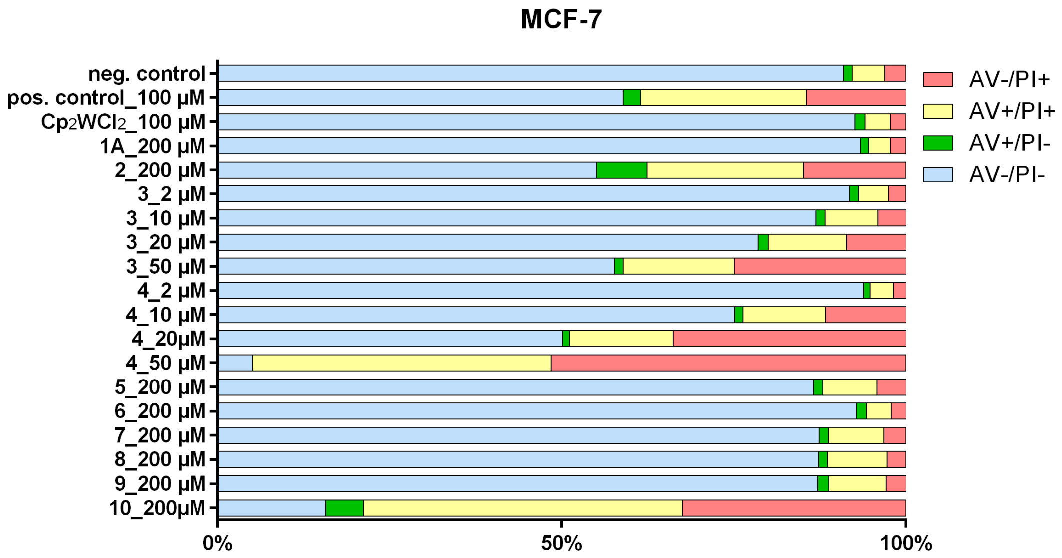

Figure S44: Apoptosis induction by Cp_2WCl_2 and ten other tungstenocenes (**1A**–**10**) after 24 h incubation in HCT116, HT29 and MCF-7 cancer cells. Cells were double-stained with annexin V-FITC and propidium iodide (PI) and analyzed by flow cytometry. Percentages of necrotic (AV-/PI+), late apoptotic (AV+/PI+), early apoptotic (AV+/PI-) and viable (AV-/PI-) cells are given as averages of at least three independent experiments. Positive control was treated with KP1998 (compound 7 in reference [62])

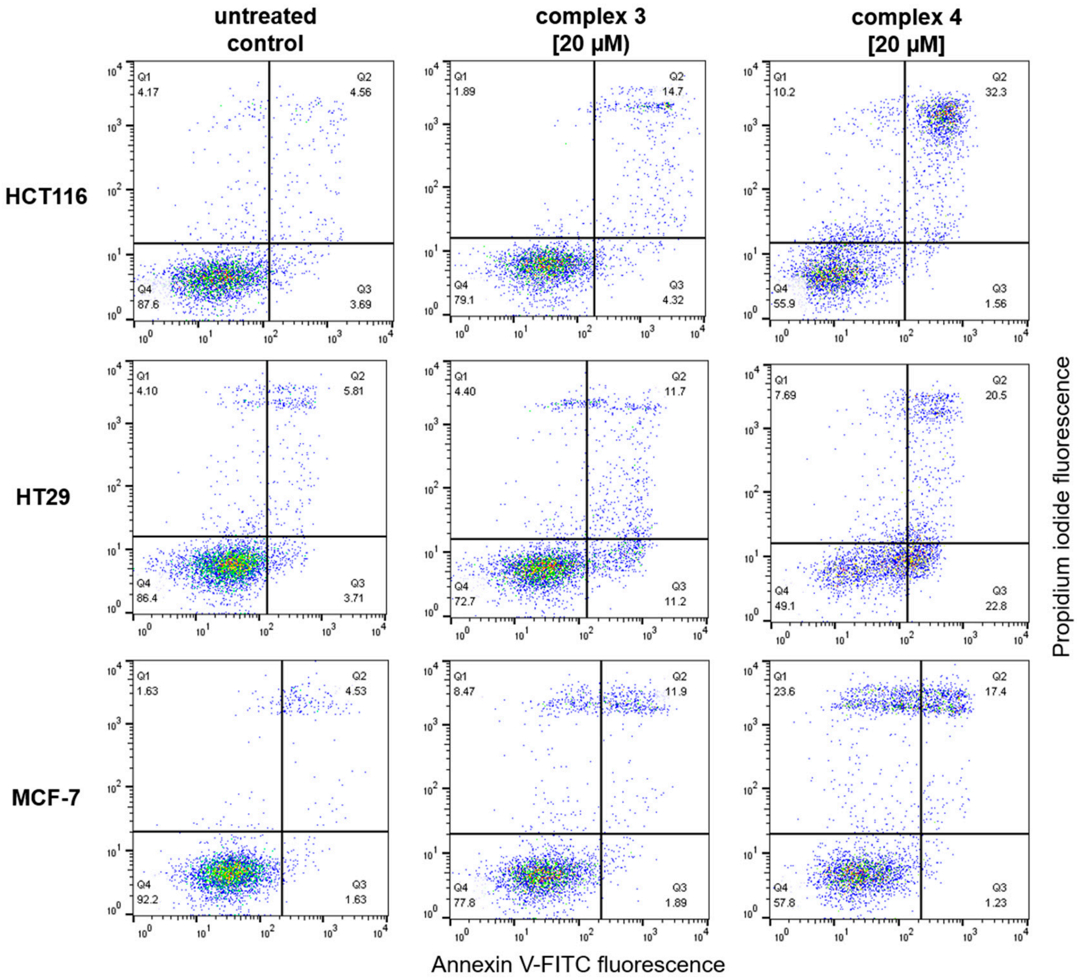

Figure S45: Dot plots of viable (Q4), early apoptotic (Q3), late apoptotic (Q2) and necrotic (Q1) HCT116, HT29 and MCF-7 cells treated with two most cytotoxic complexes **3** and **4** at 20 µM. The dot plots represent the annexin V-FITC (x-axis) and PI (y-axis) staining detected by flow cytometry and represent a single experiment out of at least three repetitions.

Table S26: Apoptosis induction by Cp_2WCl_2 and tungstenocenes containing (*O,O*–), (*S,O*–) and (*N,O*–)chelate in three different cell lines after 24 h exposure to the indicated concentrations. For each setting, the total apoptotic subpopulation is the sum of early and late apoptotic events (Σapoptotic cells [%]). Results are means ± standard derivations from at least three independent experiments. Positive control was treated with KP1998 (compound 7 in reference [63])

cell line	compound	concentration	viable cells [%] AV- / PI-	early apoptotic cells [%] AV+ / PI-	late apoptotic cells [%] AV+ / PI+	necrotic cells [%] AV- / PI+	∑apoptotic cells [%]
**HCT116**	**neg. ctrl**		88.2 ± 8.6	2.8 ± 2.7	6.1 ± 5.5	2.9 ± 1.8	8.9
	**pos. ctrl**	100 µM	16.1 ± 1.9	3.3 ± 1.6	61.7 ± 2.7	19.0 ± 4.3	65
	**Cp_2WCl_2**	100 µM	74.7 ± 4.9	7.5 ± 3.0	15.1 ± 2.1	2.7 ± 0.5	22.6
	**1A**	200 µM	74.5 ± 7.0	5.6 ± 2.1	16.1 ± 4.6	3.8 ± 2.1	21.7
	**2**	200 µM	63.5 ± 18.6	7.2 ± 1.9	27.1 ± 17.6	2.2 ± 0.2	34.3
	**3**	2 µM	77.7 ± 1.6	4.6 ± 1.7	13.5 ± 0.6	4.3 ± 0.7	18.1
		20 µM	80.4 ± 2.6	5.0 ± 0.9	12.1 ± 2.9	2.5 ± 0.6	17.1
		100 µM	8.6 ± 4.3	3.3 ± 1.1	75.5 ± 5.6	12.8 ± 0.2	78.8
		200 µM	0.4 ± 0.3	0.0 ± 0.0	94.1 ± 4.8	5.5 ± 4.9	94.1
	**4**	2 µM	87.3 ± 7.8	2.3 ± 0.0	8.0 ± 6.2	2.4 ± 1.5	10.3
		20 µM	55.5 ± 4.8	1.9 ± 0.4	31.1 ± 4.7	11.5 ± 1.2	33
	**5**	200 µM	92.7 ± 1.3	2.0 ± 1.1	2.9 ± 0.7	2.4 ± 1.2	4.9
	**6**	200 µM	74.7 ± 14.1	5.0 ± 1.3	17.2 ± 122	3.1 ± 0.7	22.2
	**7**	200 µM	78.2 ± 9.3	4.5 ± 1.8	13.7 ± 8.2	3.6 ± 0.9	18.2
	**8**	200 µM	79.4 ± 3.5	5.5 ± 2.0	12.4 ± 2.4	2.8 ± 1.3	17.9
	**9**	200 µM	93.9 ± 0.5	1.5 ± 0.8	2.3 ± 0.3	2.3 ± 0.4	3.8
	**10**	200 µM	89.6 ± 7.5	3.8 ± 3.2	4.3 ± 3.6	2.4 ± 0.7	8.0
**HT29**	**neg. ctrl**		91.6 ± 5.3	2.5 ± 2.3	2.9 ± 2.2	3.1 ± 1.2	4.5
	**pos. ctrl**	100 µM	29.2 ± 9.4	5.5 ± 1.9	49.1 ± 7.8	16.1 ± 9.7	54.6
	**Cp_2WCl_2**	100 µM	86.5 ± 0.3	4.0 ± 2.2	5.0 ± 0.3	4.6 ± 2.5	9.0
	**1A**	200 µM	87.8 ± 4.0	3.5 ± 2.5	4.3 ± 0.9	4.4 ± 4.8	7.8
	**2**	200 µM	86.8 ± 2.4	3.9 ± 1.8	6.1 ± 1.0	3.3 ± 2.4	10
	**3**	2 µM	83.0 ± 3.8	4.6 ± 4.1	7.2 ± 3.8	5.1 ± 4.2	11.8
		20 µM	74.9 ± 3.3	8.6 ± 2.5	11.1 ± 1.0	5.4 ± 1.2	19.7
		200 µM	2.2 ± 1.3	0.8 ± 0.5	82.2 ± 4.1	14.9 ± 2.8	83.0
	**4**	2 µM	86.2 ± 3.1	3.7 ± 3.2	4.8 ± 1.3	5.4 ± 5.0	8.5
		20 µM	50.4 ± 4.1	16.8 ± 5.2	19.5 ± 2.0	13.3 ± 5.1	36.3
	**5**	200 µM	91.5 ± 8.9	0.9 ± 0.7	5.3 ± 6.9	2.3 ± 1.5	6.2
	**6**	200 µM	89.1 ± 0.4	4.3 ± 1.8	4.2 ± 0.6	2.4 ± 0.7	8.5
	**7**	200 µM	88.4 ± 3.8	3.0 ± 1.6	5.5 ± 1.9	3.1 ± 2.3	8.5
	**8**	200 µM	88.4 ± 3.6	3.3 ± 2.1	6.2 ± 4.1	2.1 ± 0.9	9.5
	**9**	200 µM	92.9 ± 5.8	0.9 ± 0.6	3.9 ± 4.5	2.3 ± 1.3	4.8
	**10**	200 µM	89.5 ± 8.5	3.1 ± 2.8	5.1 ± 5.2	2.3 ± 0.9	8.2

**MCF-7**	**neg. ctrl**		90.9 ± 5.1	1.3 ± 0.7	4.7 ± 3.1	3.1 ± 1.7	3.8
	**pos. ctrl**	100 µM	59.0 ± 1.9	2.5 ± 1.1	24.1 ± 2.6	14.5 ± 1.8	26.6
	**Cp_2WCl_2**	100 µM	92.6 ± 2.1	1.5 ± 0.7	3.7 ± 1.2	2.3 ± 0.3	5.1
	**1A**	200 µM	94.3 ± 2.3	1.1 ± 0.6	3.2 ± 1.3	2.3 ± 0.5	4.3
	**2**	200 µM	55.1 ± 10.1	7.4 ± 2.7	22.7 ± 4.5	14.9 ± 8.3	30.1
	**3**	2 µM	91.8 ± 1.9	1.4 ± 0.5	4.3 ± 1.5	2.5 ± 0.6	5.6
		10 µM	86.9 ± 5.9	1.4 ± 0.3	7.7± 4.4	4.1 ± 1.8	9.1
		20 µM	78.5 ± 1.0	1.5 ± 0.6	11.4 ± 0.7	8.7 ± 0.3	12.9
		50 µM	57.7 ± 2.2	1.3 ± 0.5	16.2 ± 2.5	25 ± 0.9	17.5
	**4**	2 µM	93.9 ± 2.1	1.0 ± 0.8	3.4 ± 1.2	1.8 ± 0.0	4.4
		10 µM	75.2 ± 2.2	1.2 ± 0.5	12.0 ± 2.0	11.7 ± 4.7	13.2
		20 µM	50.2 ± 10.8	1.0 ± 0.4	15.1 ± 3.3	33.8 ± 14.5	16.0
		50 µM	5.0 ± 6.6	0.0 ± 0.0	43.4 ± 3.7	51.5 ± 10.3	43.4
	**5**	200 µM	86.6 ± 7.6	1.3 ± 0.7	7.9 ± 5.3	4.2 ±2.2	9.2
	**6**	200 µM	92.8 ± 2.2	1.4 ± 0.8	3.6 ± 1.5	2.1 ± 0.5	5.1
	**7**	200 µM	87.4 ± 6.6	1.3 ± 0.4	8.1 ± 5.5	3.2 ± 1.2	9.4
	**8**	200 µM	87.3 ± 8.3	1.2 ± 0.3	8.7 ± 6.9	2.7 ± 1.4	9.9
	**9**	200 µM	87.2 ± 7.2	1.6 ± 1.0	8.4 ± 5.5	2.9 ± 1.8	7.3
	**10**	200 µM	15.7 ± 6.0	5.5 ± 3.2	46.3 ± 3.2	32.4 ± 12.3	51.8

Electrophoretic dsDNA plasmid assay

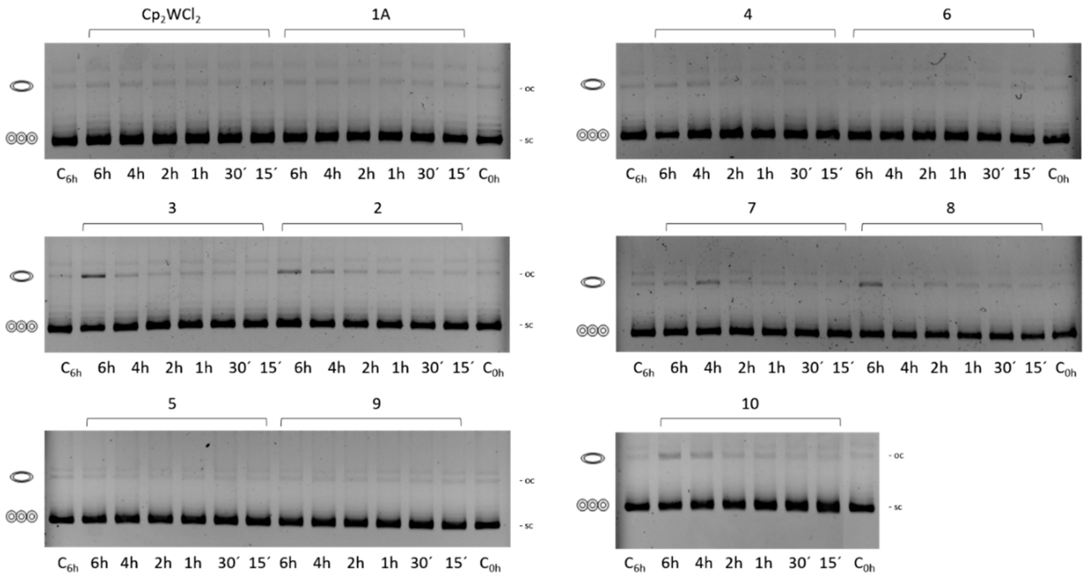

Figure S46: Electropherograms of plasmid DNA interactions with 50 µM of compounds Cp_2WCl_2 and **1A–10.** The pUC19 plasmid was incubated with the complexes for different time periods (from 15 min to 6 h) at 37 °C and the different plasmid forms were separated by agarose gel electrophoresis. ‘C_{0h}’ corresponds to an untreated, non-modified plasmid DNA control at the time point of gel loading (0 h), while ‘C_{6h}’ corresponds to an untreated control for the total duration of the experiment (6 h); oc, open circular; sc, supercoiled.

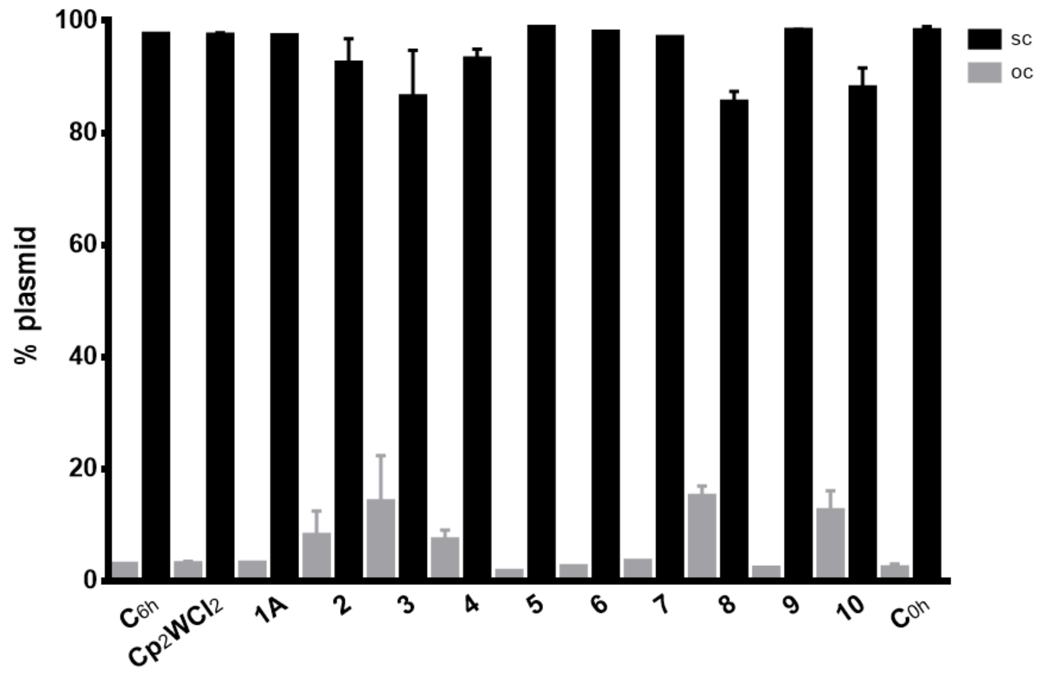

Figure S47: Bands of Figure S46 were quantitatively analyzed by the ImageJ 1.51j8 software. The black columns correspond to the open-circular (oc) form and grey columns to the supercoiled (sc) form of the pUC19 plasmid, presented as percentages of total plasmid DNA content.

References

63. Scaffidi-Domianello, Y. Y.; Legin, A.; Jakupec, M. A.; Arion, V. B.; Kukushkin, V. Y.; Galanski, M. S.; Keppler, B. K., Synthesis, characterization, and cytotoxic activity of novel potentially pH-sensitive nonclassical platinum(II) complexes featuring 1,3-dihydroxyacetone oxime ligands. *Inorg. Chem.* **2011**, *50*(21), 10673-10681.
